# Supplementary material for: Dual-color live imaging unveils stepwise organization of multiple basal body arrays by cytoskeletons
Source: EMBO Rep. 2024 Feb 5;25(3):1176–207. doi: 10.1038/s44319-024-00066-0 (PMC10933483; doi:10.1038/s44319-024-00066-0)
Supplement: Supplementary file 1 — Appendix [file 44319_2024_66_MOESM1_ESM.pdf]

Appendix

Table of Contents

Appendix Figure S1 2

Appendix Figure S1 Figure Legend 3

Appendix Figure S2 4

Appendix Figure S2 Figure Legend 5

Appendix Figure S3 and Figure Legend..........6

Appendix Figure S4 and Figure Legend..........7

Appendix Figure S5 and Figure Legend..........8

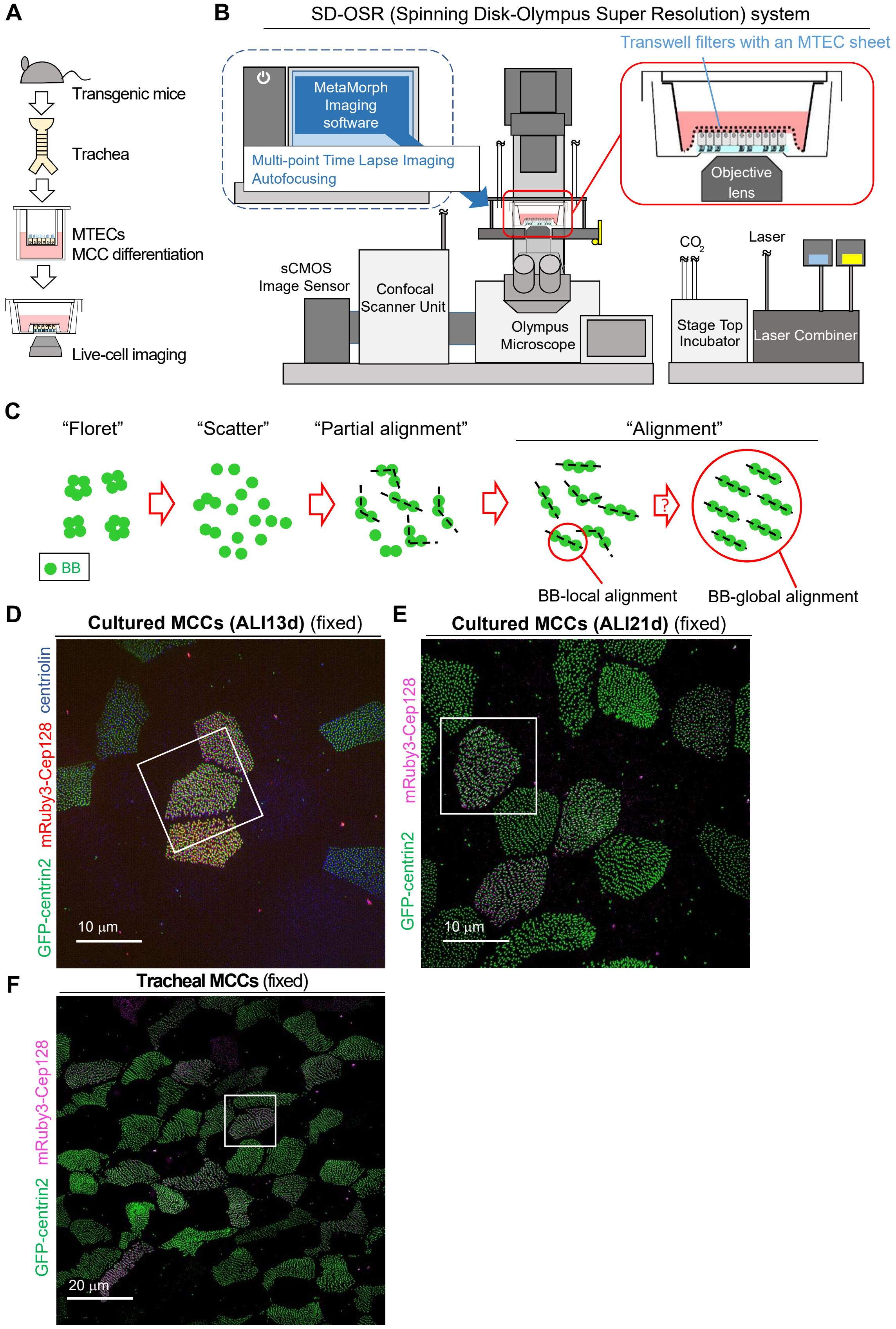
A
Transgenic mice
Trachea
MTECs
MCC differentiation
Live-cell imaging
B
SD-OSR (Spinning Disk-Olympus Super Resolution) system
MetaMorph
Imaging
software
Multi-point Time Lapse Imaging
Autofocusing
Transwell filters with an MTEC sheet
Objective
lens
sCMOS
Image Sensor
Confocal
Scanner Unit
Olympus
Microscope
CO2
Laser
Stage Top
Incubator
Laser Combiner
C
"Floret"
"Scatter"
"Partial alignment"
"Alignment"
BB
?
BB-local alignment
BB-global alignment
D
Cultured MCCs (ALI13d) (fixed)
GFP-centrin2 mRuby3-Cep128 centriolin
10 μm
E
Cultured MCCs (ALI21d) (fixed)
GFP-centrin2 mRuby3-Cep128
10 μm
F
Tracheal MCCs (fixed)
GFP-centrin2 mRuby3-Cep128
20 μm

Appendix Figure S1. Establishment of a high-resolution, dual-color live-cell imaging system for analysis of the BB-array using MTECs.

A, B Schematic diagrams of the preparation procedure for MTECs and our dual-color live-cell imaging system used in this work (see also Materials and Methods). MTECs were cultured on 24-well transwell filters (A), and the filters were removed from the wells and conversely attached to the other culture plate for observation. Dual-color live-cell imaging and data acquisition were performed using a Spinning Disk-Olympus Super-Resolution (SD-OSR) microscope system shown in (B).

C Schematic diagrams of the developmental process of BB-alignment found in our previous study. “Floret,” “Scatter,” “Partial alignment,” and “Alignment” stages were defined from the degree of BB-local alignment. However, the degree of BB-global alignment was not incorporated into this definition of the developmental stages. Black dotted lines represent the local alignment of neighboring BBs.

D Fixed MTECs (ALI13d), which were prepared from transgenic mice expressing GFP-centrin2 (green) and mRuby3-Cep128 (red), were stained with antibodies against centriolin (blue) and analyzed by spinning disk confocal microscopy. Bar, 10 μm. High-magnification images of the boxed regions are shown in Fig 1D.

E, F Spinning disk confocal microscopy of the BB-array in MCCs of fixed MTECs (ALI21d) (E) and tracheal cells (F), prepared from transgenic mice expressing GFP-centrin2 (green) and mRuby3-Cep128 (magenta). Bars, 10 and 20 μm. High-magnification images of the boxed regions are shown in Fig 1E and F.

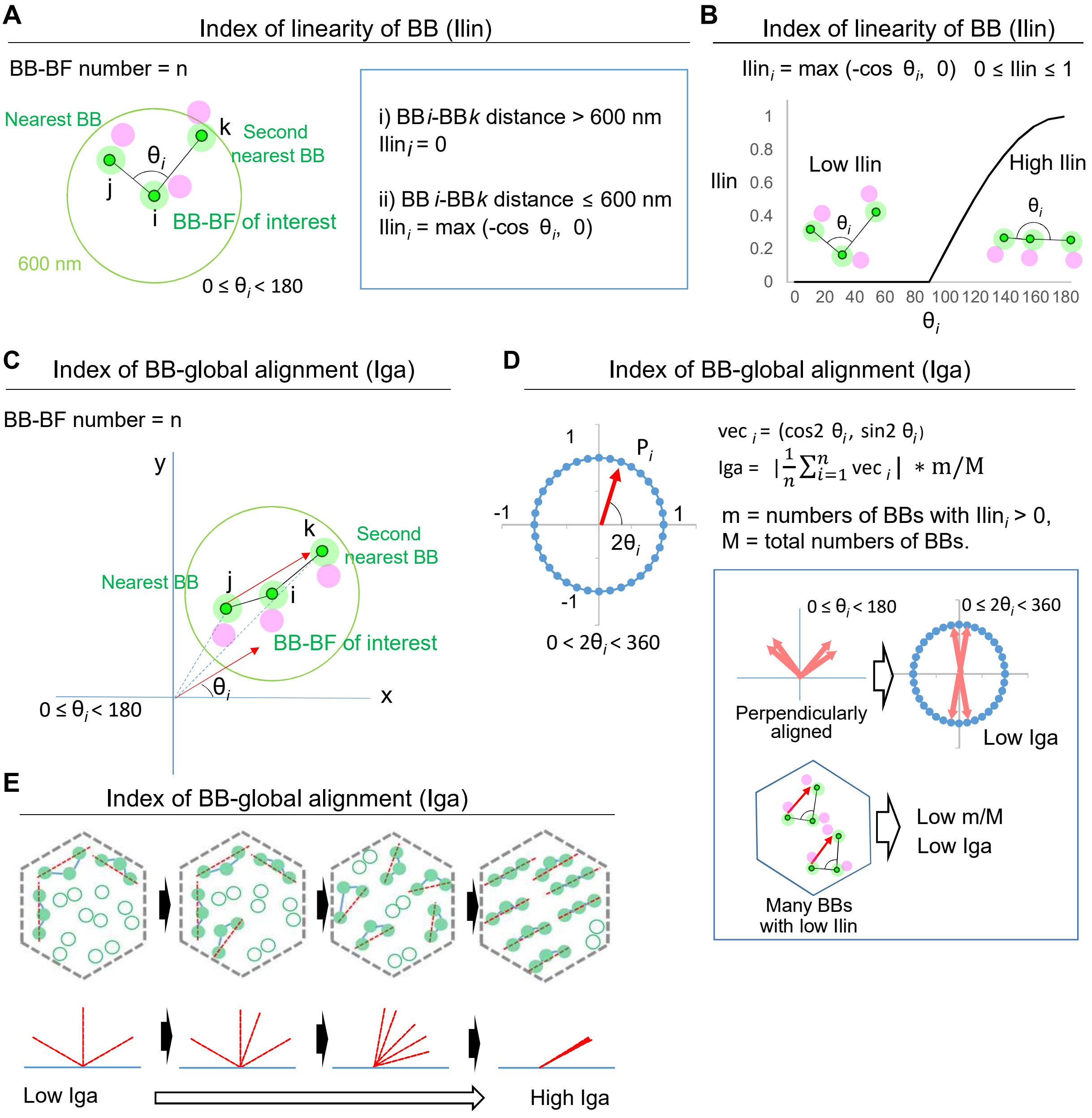

A
Index of linearity of BB (Ilin)
BB-BF number = n
Nearest BB
k
Second nearest BB
θi
j
i
BB-BF of interest
600 nm
0 ≤ θi < 180
i) BBi-BBk distance > 600 nm
Ilinj = 0
ii) BB i-BBk distance ≤ 600 nm
Ilini = max (-cos θi, 0)
B
Index of linearity of BB (Ilin)
Ilini = max (-cos θi, 0) 0 ≤ Ilin ≤ 1
Ilin
Low Ilin
High Ilin
θi
C
Index of BB-global alignment (Iga)
BB-BF number = n
y
x
Nearest BB
Second nearest BB
BB-BF of interest
0 ≤ θi < 180
D
Index of BB-global alignment (Iga)
Pi
2θi
0 < 2θi < 360
vec i = (cos2 θi, sin2 θi)
Iga = |1/n ∑ vec i| * m/M
m = numbers of BBs with Ilini > 0,
M = total numbers of BBs.
0 ≤ θi < 180
0 ≤ 2θi < 360
Perpendicularly aligned
Low Iga
Many BBs with low Ilin
Low m/M
Low Iga
E
Index of BB-global alignment (Iga)
Low Iga
High Iga

Appendix Figure S2. Quantification procedure for BB-global alignment in this work.
A, B Schematic diagrams of the quantification process for the linearity of neighboring BBs. We defined neighboring BBs, composed of three BBs, i.e., BBi (a BB of interest), BBj (the BB closest to the BBi), and BBk (the BB second closest to the BBi), and calculated $\cos\theta_i$ from the inner product and norm of the vectors (i = 1, ..., n; n = the total number of BBs; $0 \leq \theta_i < 180$, between the vectors of BBi-BBj and BBi-BBk) (A). We defined Ilin as follows: I) BBi-BBk distance > 600 nm, $Ilin_i = 0$. II) BBi-BBk distance ≤ 600 nm, $Ilin_i = \max(-\cos\theta_i, 0)$. Note that Ilin is a value between 0 and 1. If all BBs were arranged in a straight line ($\theta_i = 180$), the $Ilin_i$ was 1. If the BBs aligned in a V-shape ($0 \leq \theta_i \leq 90$), the $Ilin_i$ was 0 (B) (see also Materials and Methods).
C–E Schematic diagrams of the quantification of BB-global alignment. We defined neighboring BBs similar to the calculation of Ilin, and we calculated the $\sin 2\theta_i$ and $\cos 2\theta_i$ using the additive theorem (i = 1, ..., n; n = the total number of BBs; $0 \leq \theta_i < 180$). The unit vector vec_i was defined ($vec_i = (\cos 2\theta_i, \sin 2\theta_i)$). Note that θ_i was doubled ($0 \leq 2\theta_i < 360$) to counteract perpendicularly oriented unit vectors (C, D). To reduce the contribution from unaligned V-shaped neighboring BBs, we calculated the value of m/M (m = number of BBs with $Ilin_i > 0$, M = total number of BBs in a single cell). Finally, we calculated the absolute value of the vector vec, the mean of unit vector vec_i (i = 1, ..., n; n = the total number of BBs) and defined Iga ($Iga = |\frac{1}{n}\sum_{i=1}^{n} vec_i| * m/M$). If all neighboring BBs in a single cell showed V-shape alignment, we did not find any BB-global alignment in the cell. Using the values of Iga, we expected that the process of BB-global alignment in a single cell could be quantified (E) (see also Materials and Methods).

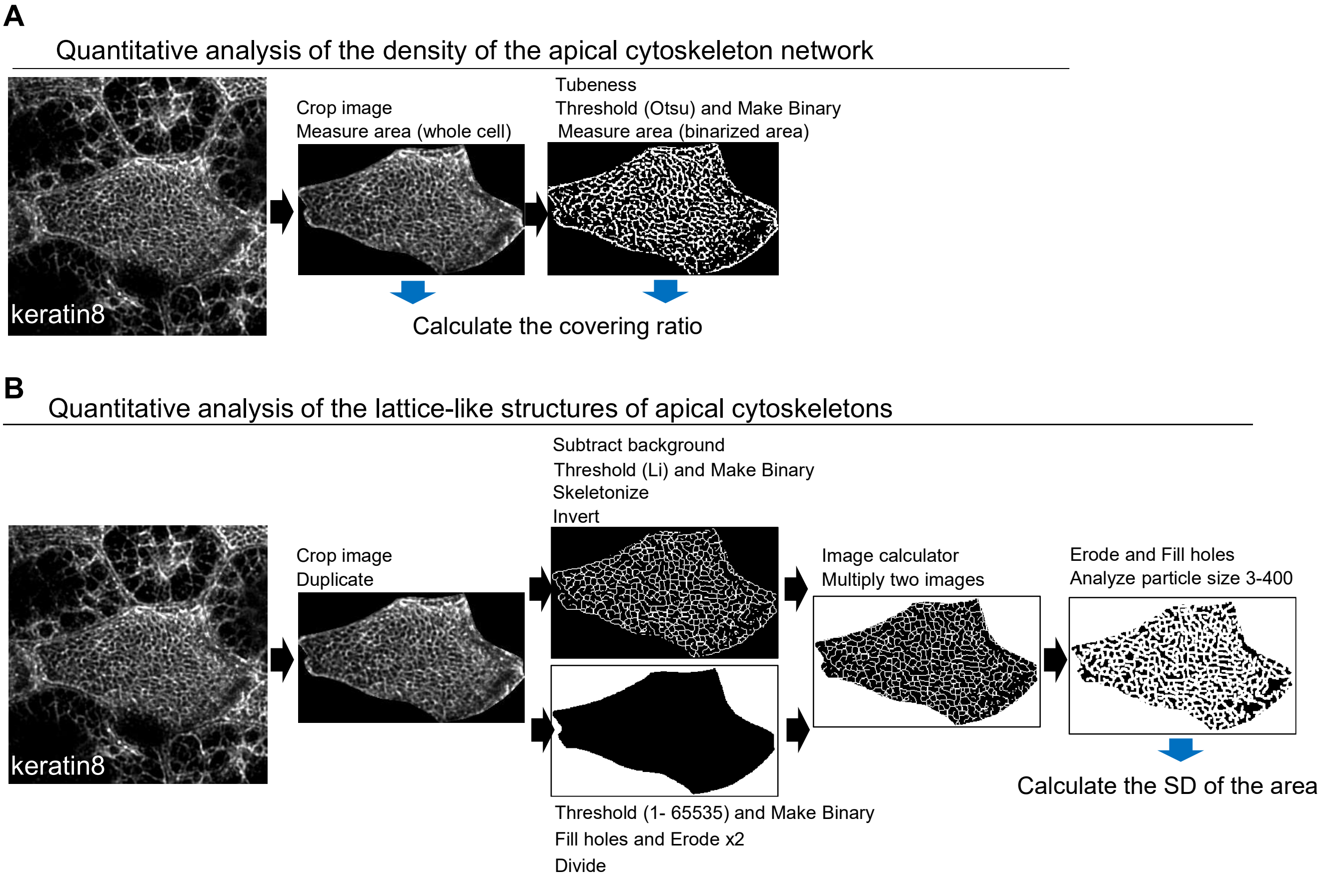

Appendix Figure S3. Quantitative analysis of the density and lattice-like structures of apical cytoskeletons. A, B Schematic diagrams of the quantification process for the density (A) and lattice-like structures (B) of apical cytoskeletons shown in Fig. 5. The keratin8-staining image of the MCC of MTEC at the late TP was shown as an example for our quantification method. See also Materials and Methods.

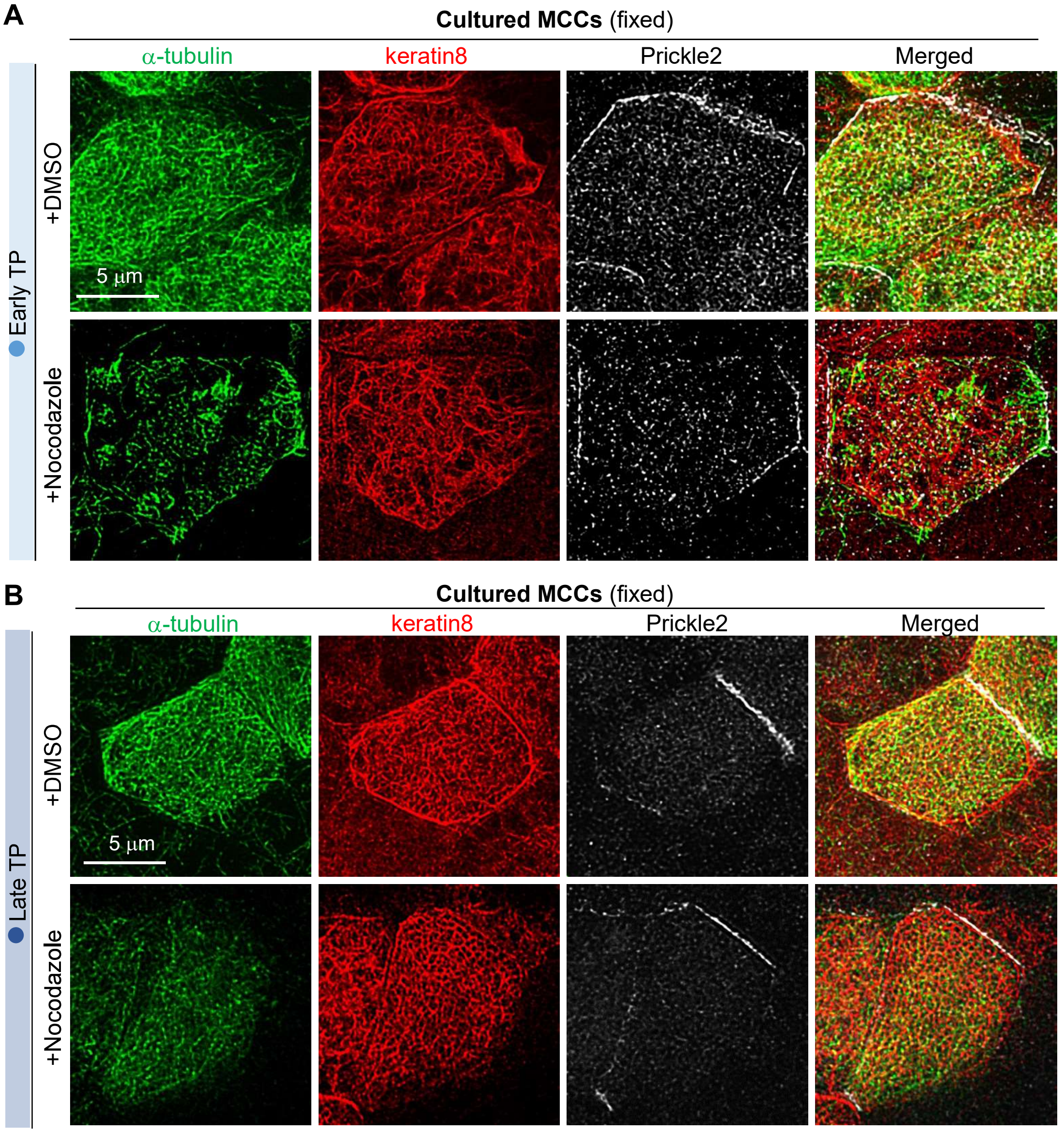

Appendix Figure S4. Confirmation of the effects of nocodazole treatment on MCCs of MTECs at different TPs.

A, B Spinning disk confocal microscopy of MCCs of fixed MTECs prepared from transgenic mice expressing GFP-centrin2 and mRuby3-Cep128 at early (A) and late (B) TPs after treatment with DMSO (upper panels) or 6.6 μM nocodazole (lower panels) for 2 h. MCCs of MTECs were fixed and stained using anti-α-tubulin (green), anti-keratin8 (red), and anti-Prickle2 (gray) antibodies. Bar, 5 μm.

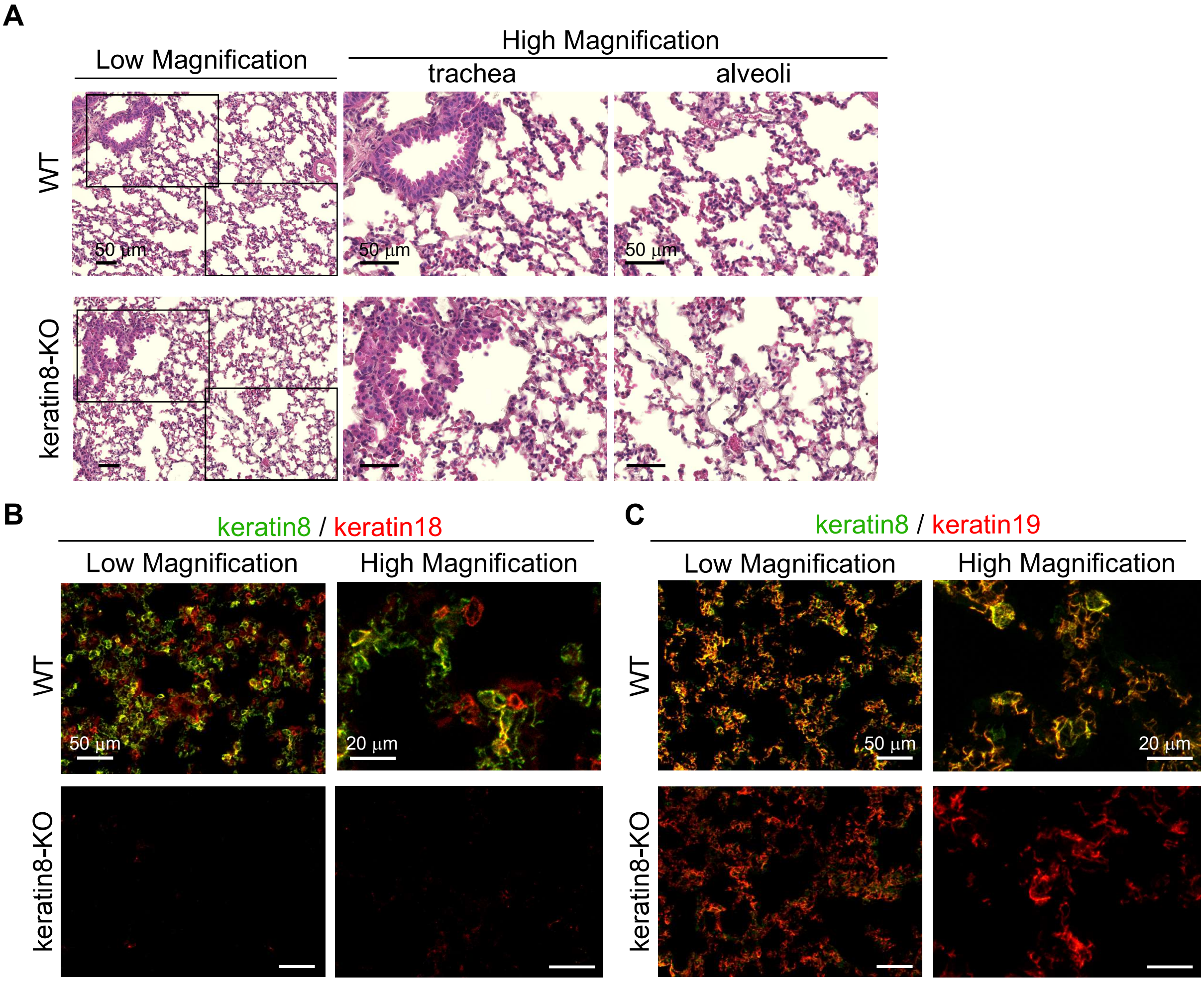

Appendix Figure S5. Observations of the lung tissues in keratin8-KO mice.
A Hematoxylin- and eosin-stained alveolar tissue sections of wildtype (upper panels) and keratin8-KO mice expressing GFP-centrin2 and mRuby3-Cep128 (lower panels). Bar, 50 µm.
B–C Microscopy of fixed alveolar tissue sections prepared from wild-type and keratin8-KO mice expressing GFP-centrin2 and mRuby3-Cep128. Tissues were stained using anti-keratin8 antibody (green) and keratin18 (red in B), keratin19 (red in C). Bar, 50 and 20 µm.
