# Supplementary figures and images for: Dual-color live imaging unveils stepwise organization of multiple basal body arrays by cytoskeletons (part 1 of 2)
Source: EMBO Rep. 2024 Feb 5;25(3):1176–207. doi: 10.1038/s44319-024-00066-0 (PMC10933483; doi:10.1038/s44319-024-00066-0)

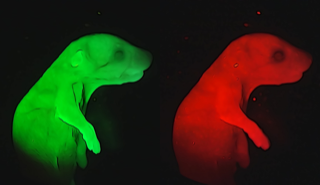

Supplement: Supplementary file 13 — Source Data Fig. 1 [file 44319_2024_66_MOESM13_ESM.zip › Source_Data_Figure_1/1A_transgenic_mice/1A_transgenic_mice.tif]

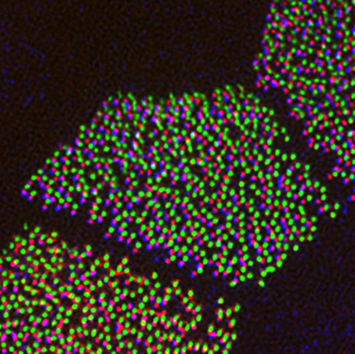

Supplement: Supplementary file 13 — Source Data Fig. 1 [file 44319_2024_66_MOESM13_ESM.zip › Source_Data_Figure_1/1D(S1D)_MTEC13d/1D_MTEC13d.tif]

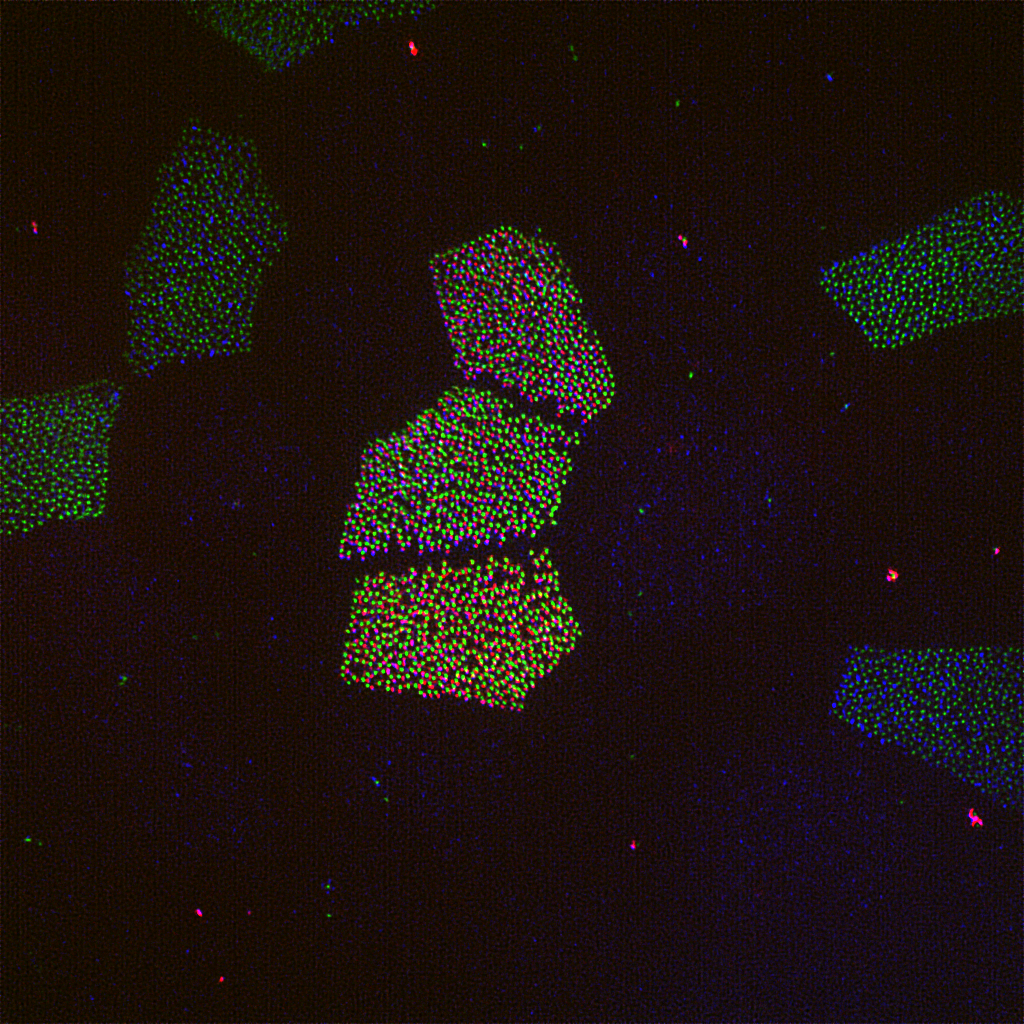

Supplement: Supplementary file 13 — Source Data Fig. 1 [file 44319_2024_66_MOESM13_ESM.zip › Source_Data_Figure_1/1D(S1D)_MTEC13d/S1D_MTEC13d_Large.tif]

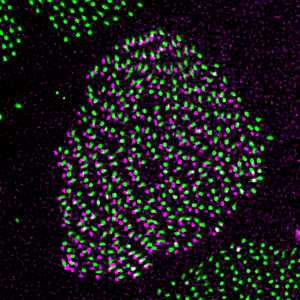

Supplement: Supplementary file 13 — Source Data Fig. 1 [file 44319_2024_66_MOESM13_ESM.zip › Source_Data_Figure_1/1E(S1E)_MTEC21d/1E_MTEC21d.tif]

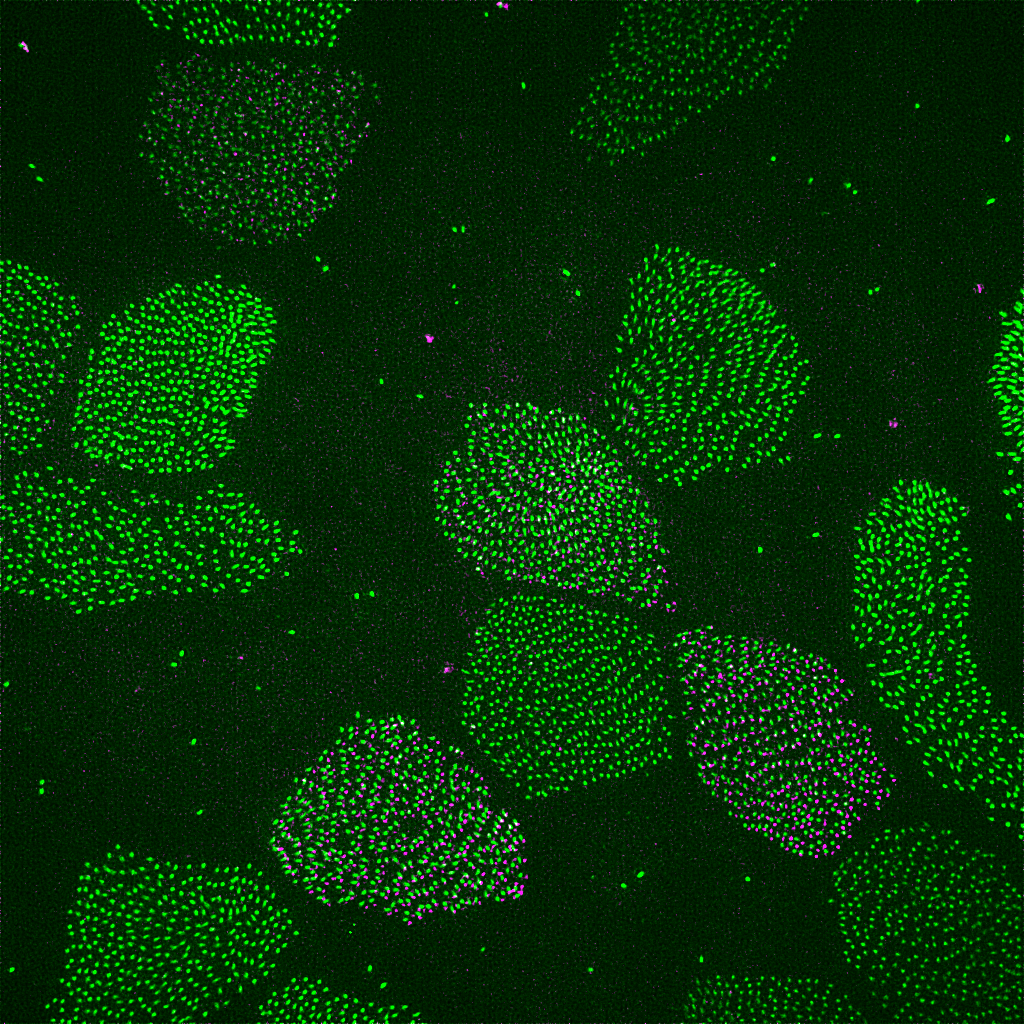

Supplement: Supplementary file 13 — Source Data Fig. 1 [file 44319_2024_66_MOESM13_ESM.zip › Source_Data_Figure_1/1E(S1E)_MTEC21d/S1E_MTEC21d_Large.tif]

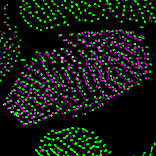

Supplement: Supplementary file 13 — Source Data Fig. 1 [file 44319_2024_66_MOESM13_ESM.zip › Source_Data_Figure_1/1F(S1F)_Trachea/1F_Trachea.tif]

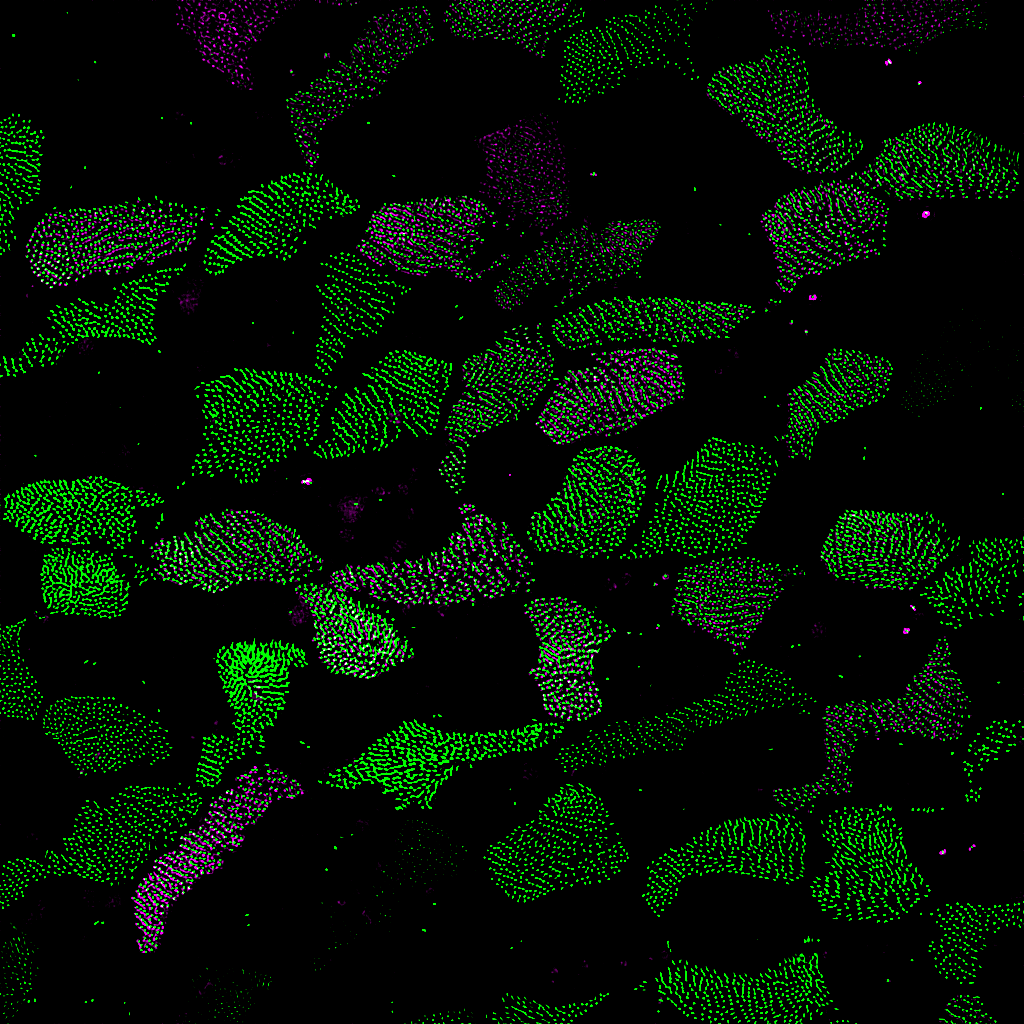

Supplement: Supplementary file 13 — Source Data Fig. 1 [file 44319_2024_66_MOESM13_ESM.zip › Source_Data_Figure_1/1F(S1F)_Trachea/S1F_Trachea_Large.tif]

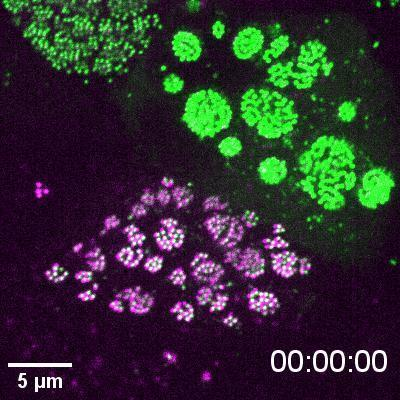

Supplement: Supplementary file 14 — Source Data Fig. 2 [file 44319_2024_66_MOESM14_ESM.zip › Source_Data_Figure_2/2B_MTEC_Initial_TP_1/2B_Initial_TP_1_Movie_EV2(23_images).tif]

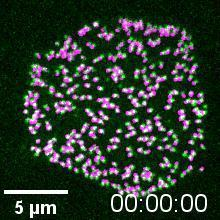

Supplement: Supplementary file 14 — Source Data Fig. 2 [file 44319_2024_66_MOESM14_ESM.zip › Source_Data_Figure_2/2C_MTEC_Early_TP_1/2C_Early_TP_1_Movie_EV3(70_images).tif]

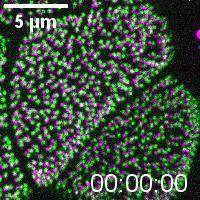

Supplement: Supplementary file 14 — Source Data Fig. 2 [file 44319_2024_66_MOESM14_ESM.zip › Source_Data_Figure_2/2D_MTEC_Early_TP_2/2D_Early_TP_2_Movie_EV4(38_images).tif]

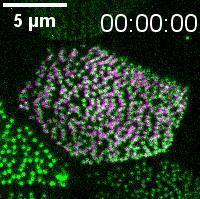

Supplement: Supplementary file 14 — Source Data Fig. 2 [file 44319_2024_66_MOESM14_ESM.zip › Source_Data_Figure_2/2E_MTEC_Late_TP_1/2E_Late_TP_1_Movie_EV5(20_images).tif]

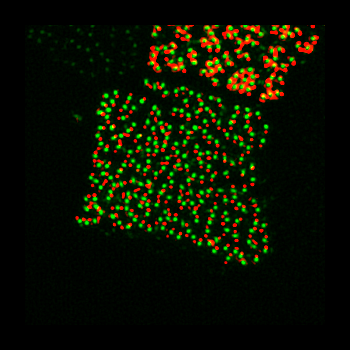

Supplement: Supplementary file 15 — Source Data Fig. 3 [file 44319_2024_66_MOESM15_ESM.zip › Source_Data_Figure_3/3B-F(EV2A-D)_Analysis/3_and_EV2_Early_TP_Analysis(Cell_1-30).tif]

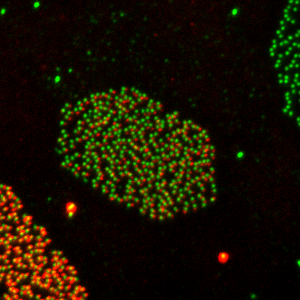

Supplement: Supplementary file 15 — Source Data Fig. 3 [file 44319_2024_66_MOESM15_ESM.zip › Source_Data_Figure_3/3B-F(EV2A-D)_Analysis/3_and_EV2_Early_TP_Analysis(Cell_31-41).tif]

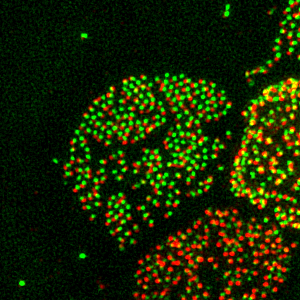

Supplement: Supplementary file 15 — Source Data Fig. 3 [file 44319_2024_66_MOESM15_ESM.zip › Source_Data_Figure_3/3B-F(EV2A-D)_Analysis/3_and_EV2_Initial_TP_Analysis(Cell_1-16).tif]

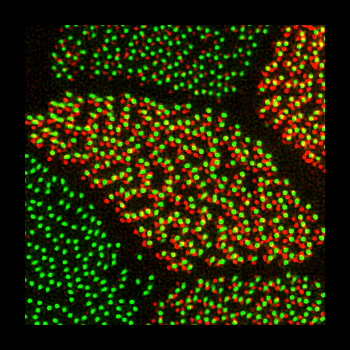

Supplement: Supplementary file 15 — Source Data Fig. 3 [file 44319_2024_66_MOESM15_ESM.zip › Source_Data_Figure_3/3B-F(EV2A-D)_Analysis/3_and_EV2_Late_TP_Analysis(Cell_1-56).tif]

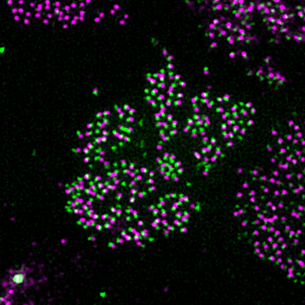

Supplement: Supplementary file 15 — Source Data Fig. 3 [file 44319_2024_66_MOESM15_ESM.zip › Source_Data_Figure_3/3F_MTEC_Stage1-5/3F_Stage1.tif]

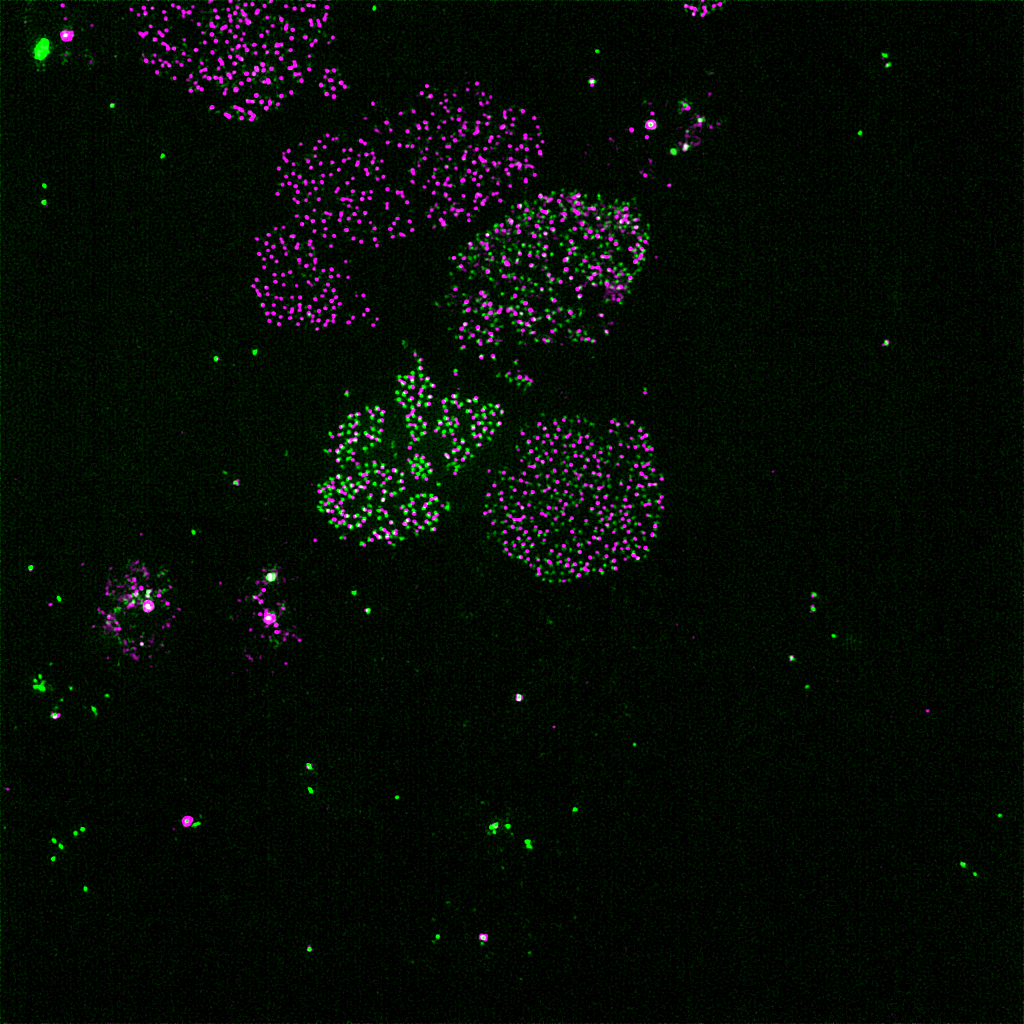

Supplement: Supplementary file 15 — Source Data Fig. 3 [file 44319_2024_66_MOESM15_ESM.zip › Source_Data_Figure_3/3F_MTEC_Stage1-5/3F_Stage1_Large.tif]

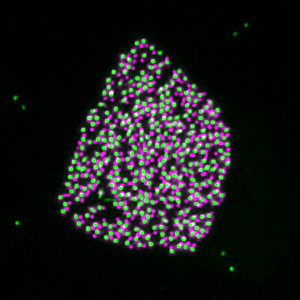

Supplement: Supplementary file 15 — Source Data Fig. 3 [file 44319_2024_66_MOESM15_ESM.zip › Source_Data_Figure_3/3F_MTEC_Stage1-5/3F_Stage2.tif]

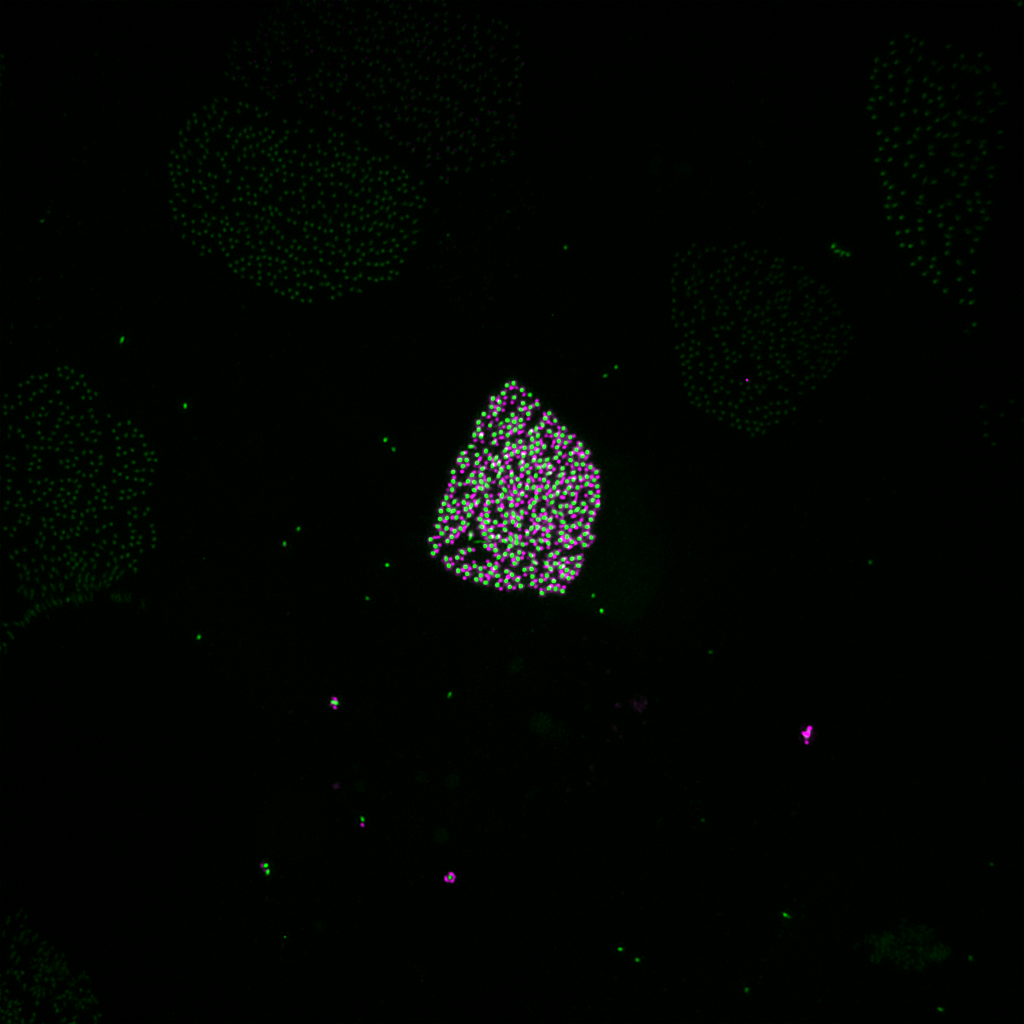

Supplement: Supplementary file 15 — Source Data Fig. 3 [file 44319_2024_66_MOESM15_ESM.zip › Source_Data_Figure_3/3F_MTEC_Stage1-5/3F_Stage2_Large.tif]

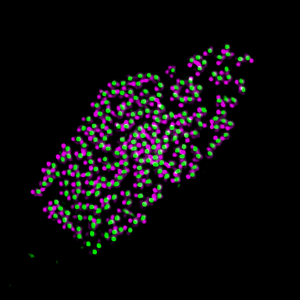

Supplement: Supplementary file 15 — Source Data Fig. 3 [file 44319_2024_66_MOESM15_ESM.zip › Source_Data_Figure_3/3F_MTEC_Stage1-5/3F_Stage3.tif]

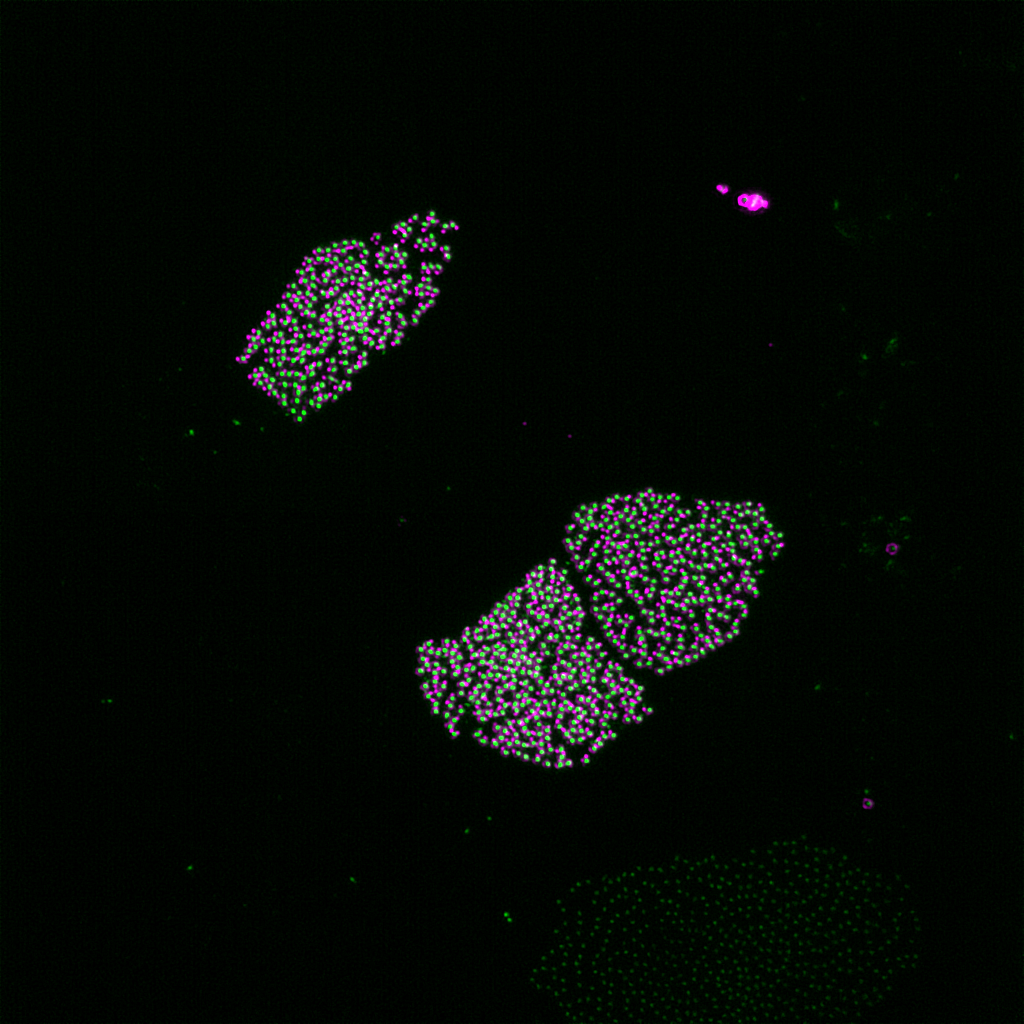

Supplement: Supplementary file 15 — Source Data Fig. 3 [file 44319_2024_66_MOESM15_ESM.zip › Source_Data_Figure_3/3F_MTEC_Stage1-5/3F_Stage3_Large.tif]

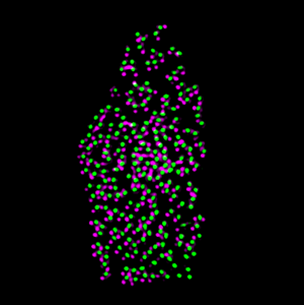

Supplement: Supplementary file 15 — Source Data Fig. 3 [file 44319_2024_66_MOESM15_ESM.zip › Source_Data_Figure_3/3F_MTEC_Stage1-5/3F_Stage3_rotated.tif]

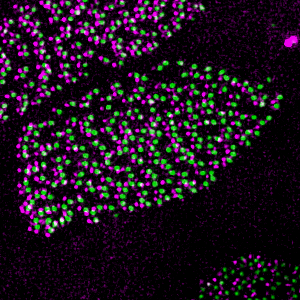

Supplement: Supplementary file 15 — Source Data Fig. 3 [file 44319_2024_66_MOESM15_ESM.zip › Source_Data_Figure_3/3F_MTEC_Stage1-5/3F_Stage4.tif]

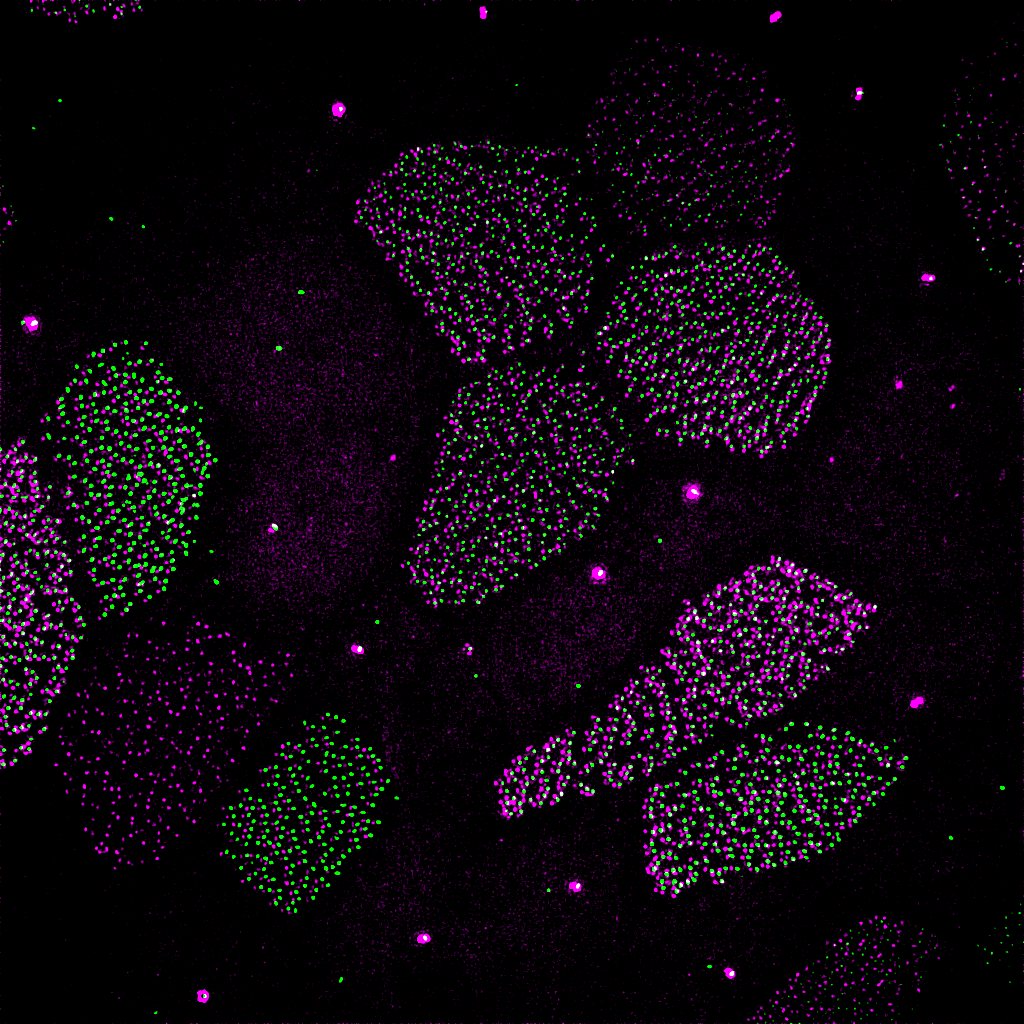

Supplement: Supplementary file 15 — Source Data Fig. 3 [file 44319_2024_66_MOESM15_ESM.zip › Source_Data_Figure_3/3F_MTEC_Stage1-5/3F_Stage4_Large.tif]

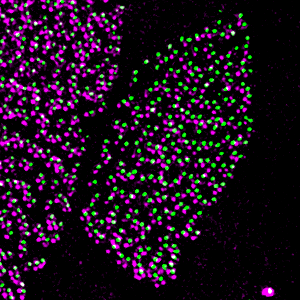

Supplement: Supplementary file 15 — Source Data Fig. 3 [file 44319_2024_66_MOESM15_ESM.zip › Source_Data_Figure_3/3F_MTEC_Stage1-5/3F_Stage4_rotated.tif]

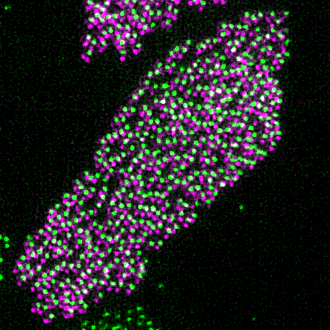

Supplement: Supplementary file 15 — Source Data Fig. 3 [file 44319_2024_66_MOESM15_ESM.zip › Source_Data_Figure_3/3F_MTEC_Stage1-5/3F_Stage5.tif]

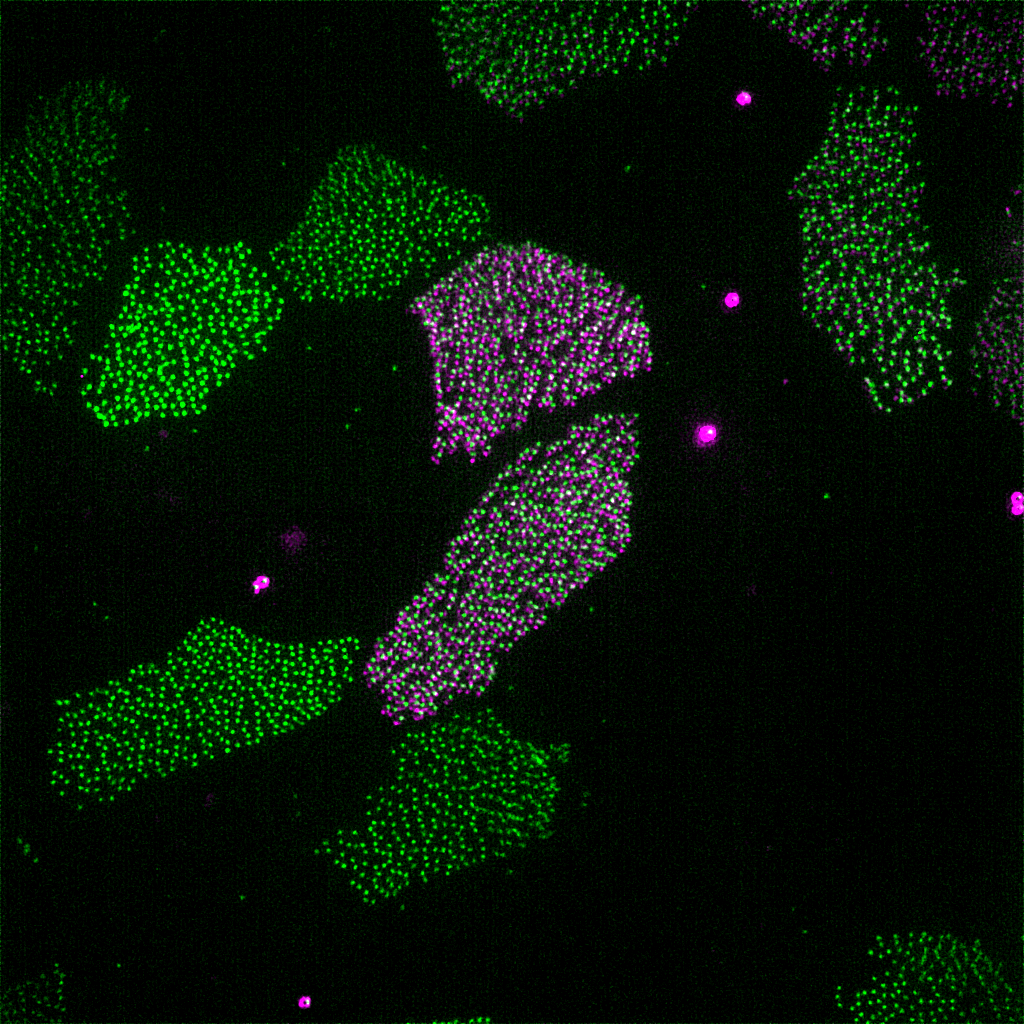

Supplement: Supplementary file 15 — Source Data Fig. 3 [file 44319_2024_66_MOESM15_ESM.zip › Source_Data_Figure_3/3F_MTEC_Stage1-5/3F_Stage5_Large.tif]

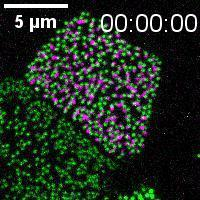

Supplement: Supplementary file 16 — Source Data Fig. 4 [file 44319_2024_66_MOESM16_ESM.zip › Source_Data_Figure_4/4B_MTEC_Live/4B_Early_TP_Stage2_Movie_EV6(35_images).tif]

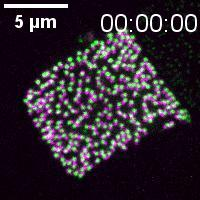

Supplement: Supplementary file 16 — Source Data Fig. 4 [file 44319_2024_66_MOESM16_ESM.zip › Source_Data_Figure_4/4B_MTEC_Live/4B_Early_TP_Stage3_Movie_EV7(48_images).tif]

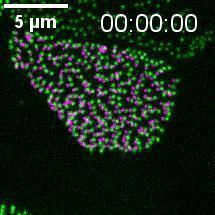

Supplement: Supplementary file 16 — Source Data Fig. 4 [file 44319_2024_66_MOESM16_ESM.zip › Source_Data_Figure_4/4C_MTEC_Live/4C_Early_TP_Stage4_Movie_EV8(37_images).tif]

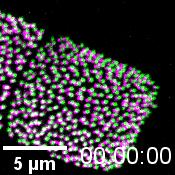

Supplement: Supplementary file 16 — Source Data Fig. 4 [file 44319_2024_66_MOESM16_ESM.zip › Source_Data_Figure_4/4C_MTEC_Live/4C_Late_TP_Stage4_Movie_EV9(50_images).tif]

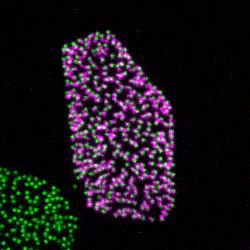

Supplement: Supplementary file 16 — Source Data Fig. 4 [file 44319_2024_66_MOESM16_ESM.zip › Source_Data_Figure_4/4D_MTEC_Live/4D_Early_TP_1(50_images).tif]

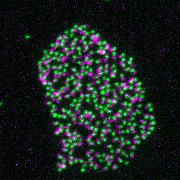

Supplement: Supplementary file 16 — Source Data Fig. 4 [file 44319_2024_66_MOESM16_ESM.zip › Source_Data_Figure_4/4D_MTEC_Live/4D_Early_TP_5(30_images).tif]

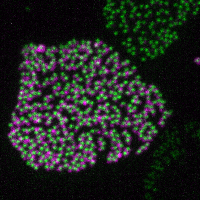

Supplement: Supplementary file 16 — Source Data Fig. 4 [file 44319_2024_66_MOESM16_ESM.zip › Source_Data_Figure_4/4D_MTEC_Live/4D_Early_TP_6(50_images).tif]

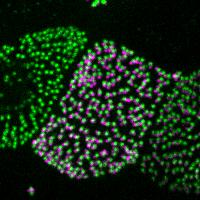

Supplement: Supplementary file 16 — Source Data Fig. 4 [file 44319_2024_66_MOESM16_ESM.zip › Source_Data_Figure_4/4D_MTEC_Live/4D_Early_TP_7(30_images).tif]

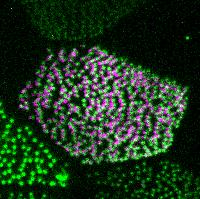

Supplement: Supplementary file 16 — Source Data Fig. 4 [file 44319_2024_66_MOESM16_ESM.zip › Source_Data_Figure_4/4D_MTEC_Live/4D_Late_TP_10(20_images)(MovieEV5).tif]

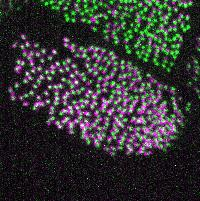

Supplement: Supplementary file 16 — Source Data Fig. 4 [file 44319_2024_66_MOESM16_ESM.zip › Source_Data_Figure_4/4D_MTEC_Live/4D_Late_TP_11(45_images).tif]

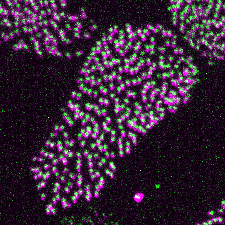

Supplement: Supplementary file 16 — Source Data Fig. 4 [file 44319_2024_66_MOESM16_ESM.zip › Source_Data_Figure_4/4D_MTEC_Live/4D_Late_TP_12(50_images).tif]

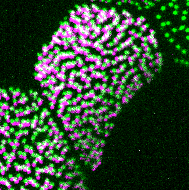

Supplement: Supplementary file 16 — Source Data Fig. 4 [file 44319_2024_66_MOESM16_ESM.zip › Source_Data_Figure_4/4D_MTEC_Live/4D_Late_TP_9(50_images).tif]

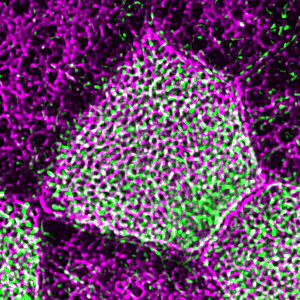

Supplement: Supplementary file 17 — Source Data Fig. 5 [file 44319_2024_66_MOESM17_ESM.zip › Source_Data_Figure_5/5A_MTEC_tubulin_keratin8/5A_tubulin_keratin8_Late_TP.tif]

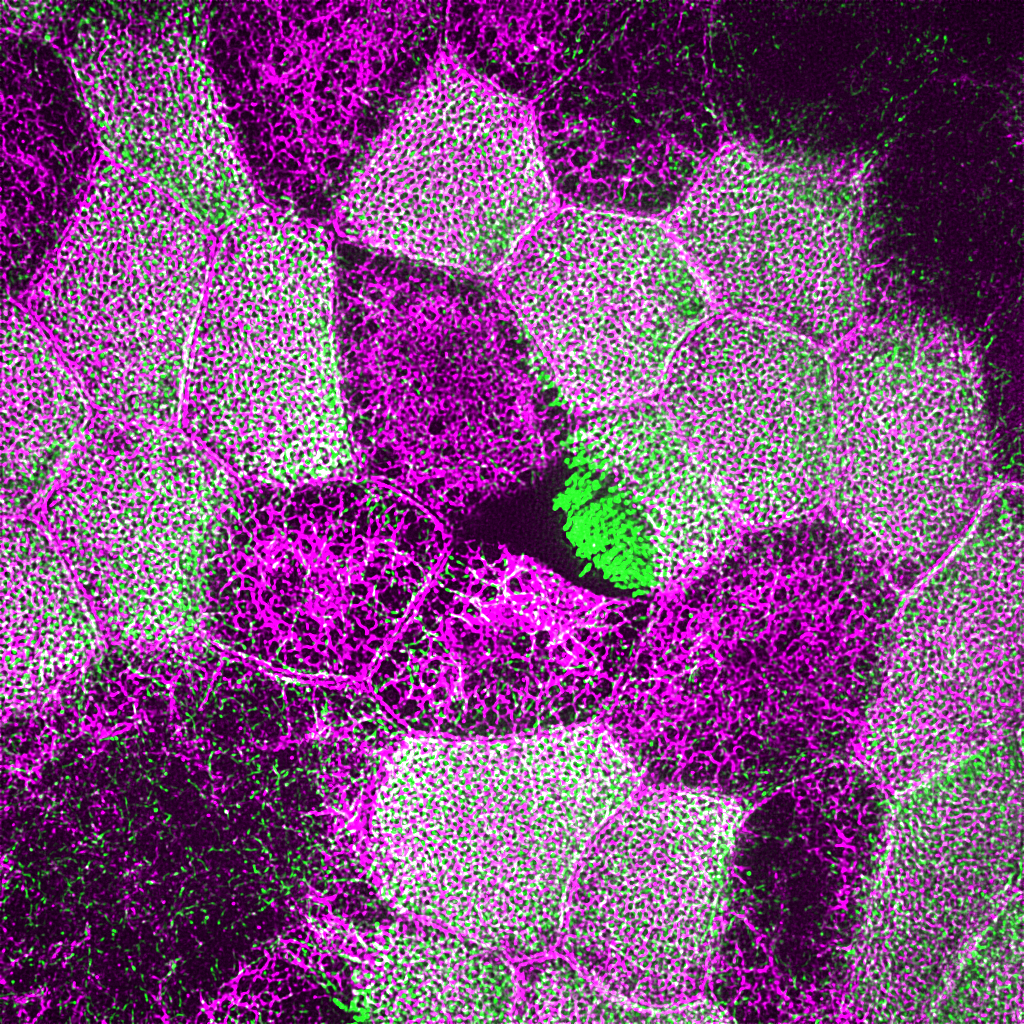

Supplement: Supplementary file 17 — Source Data Fig. 5 [file 44319_2024_66_MOESM17_ESM.zip › Source_Data_Figure_5/5A_MTEC_tubulin_keratin8/5A_tubulin_keratin8_Late_TP_Large.tif]

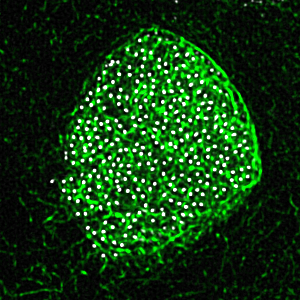

Supplement: Supplementary file 17 — Source Data Fig. 5 [file 44319_2024_66_MOESM17_ESM.zip › Source_Data_Figure_5/5B_MTEC_tubulin/5B_tubulin_Early_TP_1.tif]

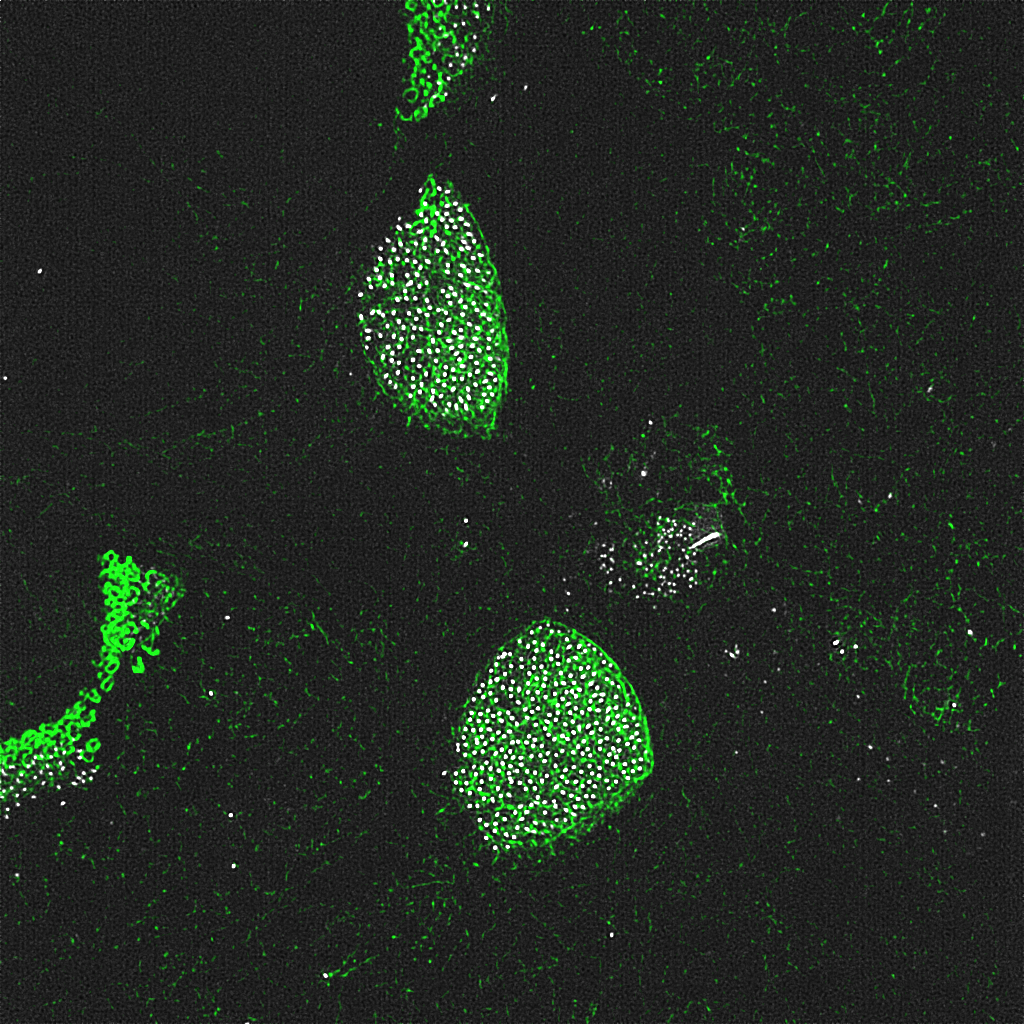

Supplement: Supplementary file 17 — Source Data Fig. 5 [file 44319_2024_66_MOESM17_ESM.zip › Source_Data_Figure_5/5B_MTEC_tubulin/5B_tubulin_Early_TP_1_Large.tif]

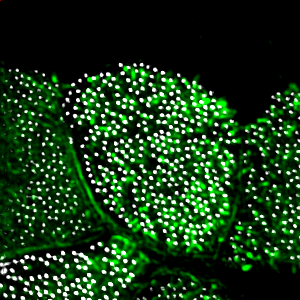

Supplement: Supplementary file 17 — Source Data Fig. 5 [file 44319_2024_66_MOESM17_ESM.zip › Source_Data_Figure_5/5B_MTEC_tubulin/5B_tubulin_Initial_TP_1.tif]

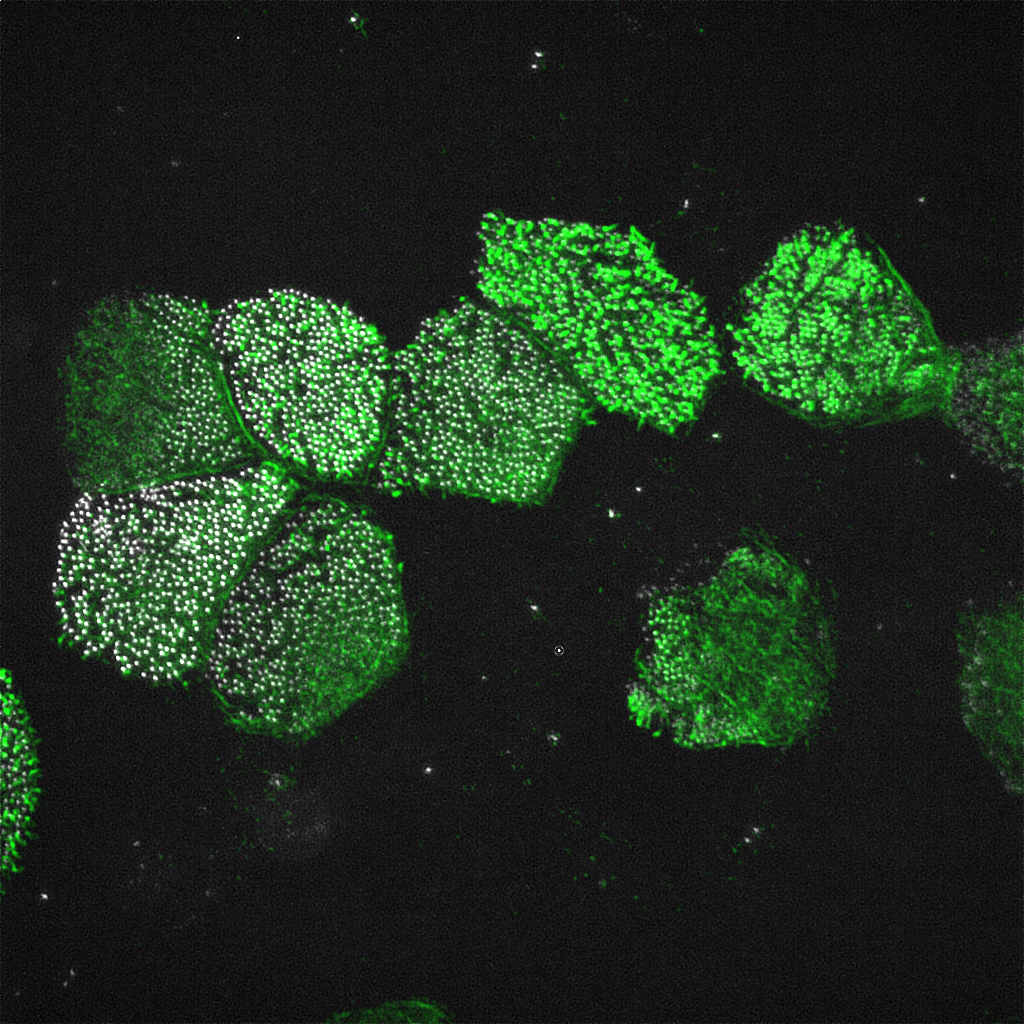

Supplement: Supplementary file 17 — Source Data Fig. 5 [file 44319_2024_66_MOESM17_ESM.zip › Source_Data_Figure_5/5B_MTEC_tubulin/5B_tubulin_Initial_TP_1_Large.tif]

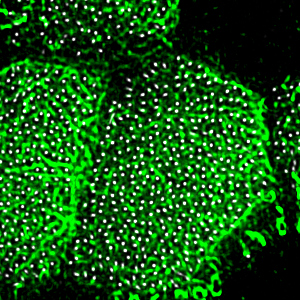

Supplement: Supplementary file 17 — Source Data Fig. 5 [file 44319_2024_66_MOESM17_ESM.zip › Source_Data_Figure_5/5B_MTEC_tubulin/5B_tubulin_Late_TP_1.tif]

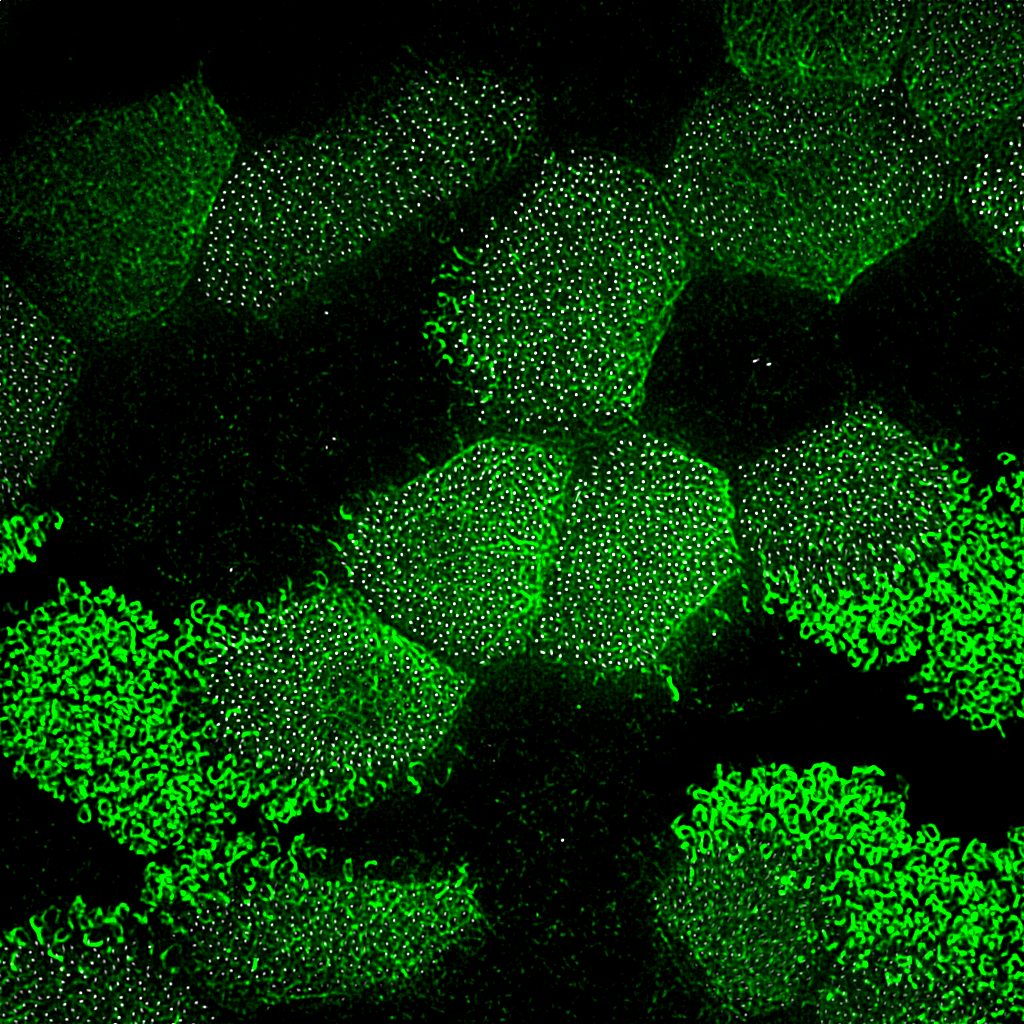

Supplement: Supplementary file 17 — Source Data Fig. 5 [file 44319_2024_66_MOESM17_ESM.zip › Source_Data_Figure_5/5B_MTEC_tubulin/5B_tubulin_Late_TP_1_Large.tif]

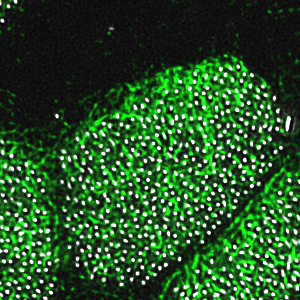

Supplement: Supplementary file 17 — Source Data Fig. 5 [file 44319_2024_66_MOESM17_ESM.zip › Source_Data_Figure_5/5B_MTEC_tubulin/5B_tubulin_Late_TP_2.tif]

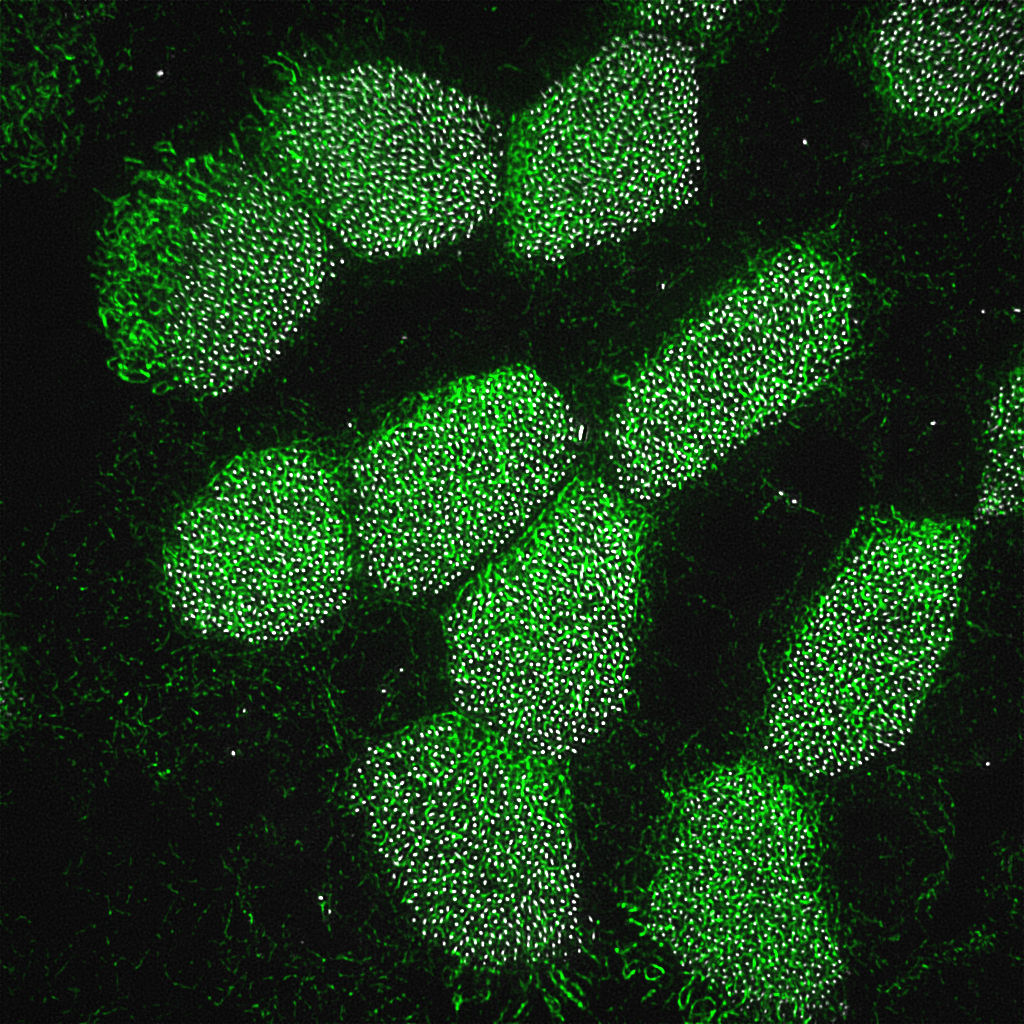

Supplement: Supplementary file 17 — Source Data Fig. 5 [file 44319_2024_66_MOESM17_ESM.zip › Source_Data_Figure_5/5B_MTEC_tubulin/5B_tubulin_Late_TP_2_Large.tif]

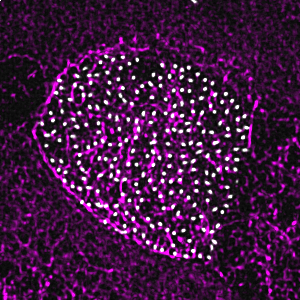

Supplement: Supplementary file 17 — Source Data Fig. 5 [file 44319_2024_66_MOESM17_ESM.zip › Source_Data_Figure_5/5C_MTEC_keratin8/5C_keratin8_Early_TP_1.tif]

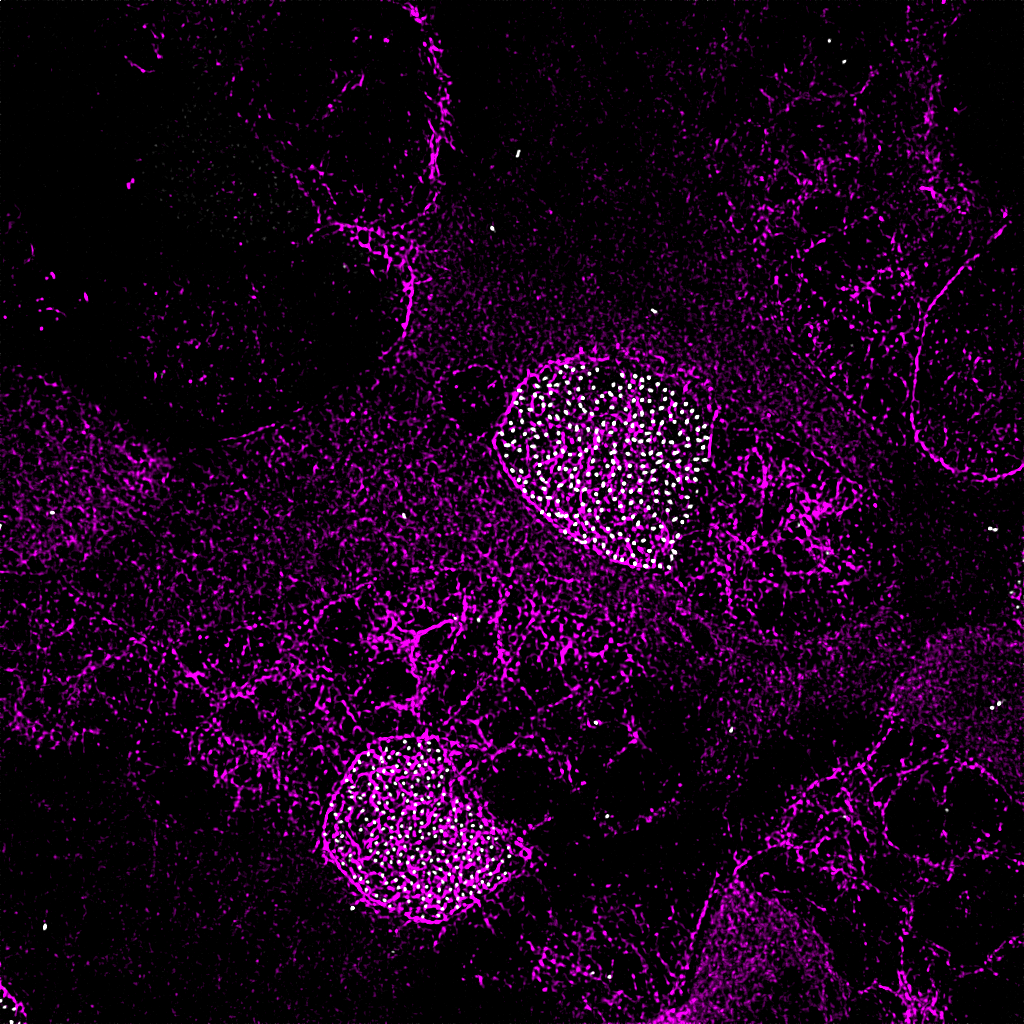

Supplement: Supplementary file 17 — Source Data Fig. 5 [file 44319_2024_66_MOESM17_ESM.zip › Source_Data_Figure_5/5C_MTEC_keratin8/5C_keratin8_Early_TP_1_Large.tif]

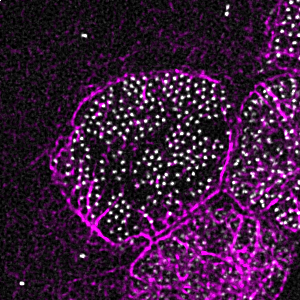

Supplement: Supplementary file 17 — Source Data Fig. 5 [file 44319_2024_66_MOESM17_ESM.zip › Source_Data_Figure_5/5C_MTEC_keratin8/5C_keratin8_Initial_TP_1.tif]

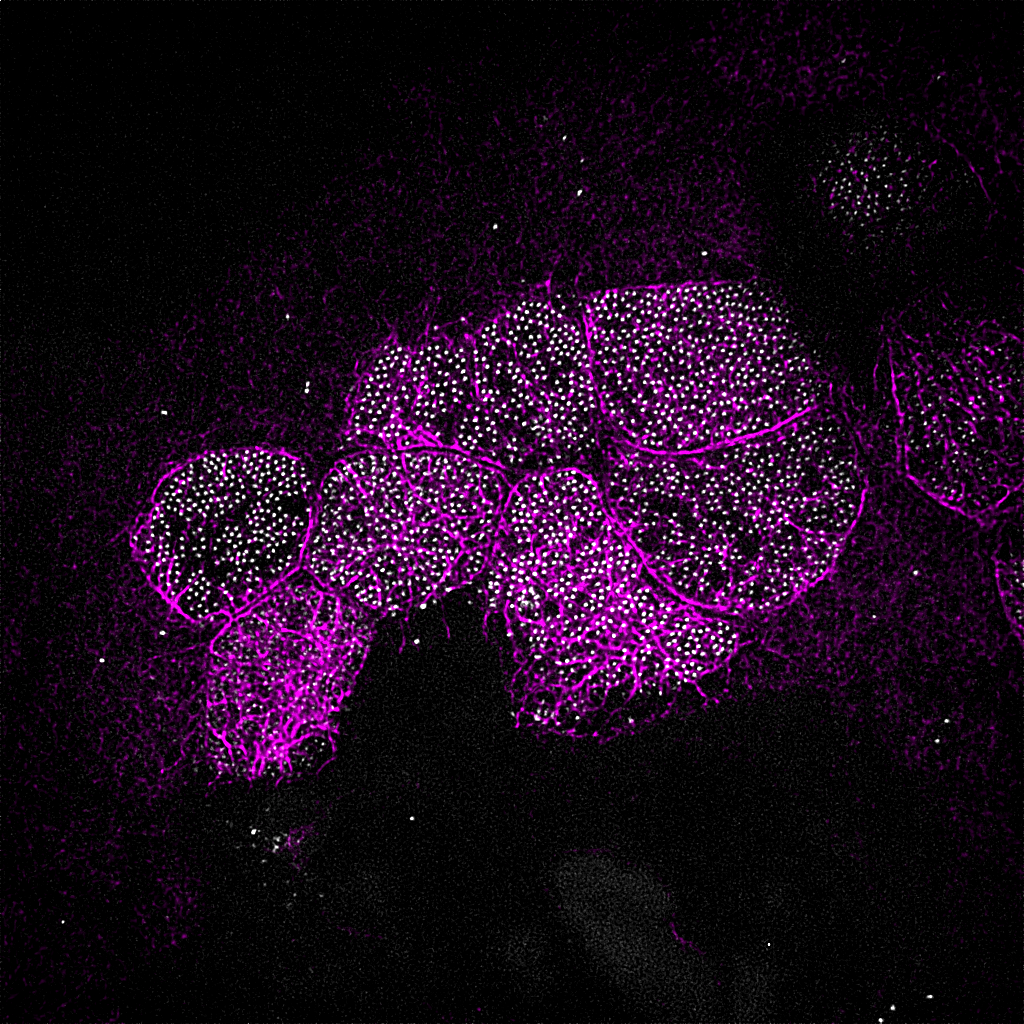

Supplement: Supplementary file 17 — Source Data Fig. 5 [file 44319_2024_66_MOESM17_ESM.zip › Source_Data_Figure_5/5C_MTEC_keratin8/5C_keratin8_Initial_TP_1_Large.tif]

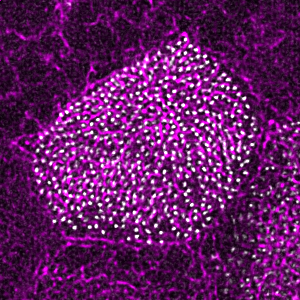

Supplement: Supplementary file 17 — Source Data Fig. 5 [file 44319_2024_66_MOESM17_ESM.zip › Source_Data_Figure_5/5C_MTEC_keratin8/5C_keratin8_Late_TP_1.tif]

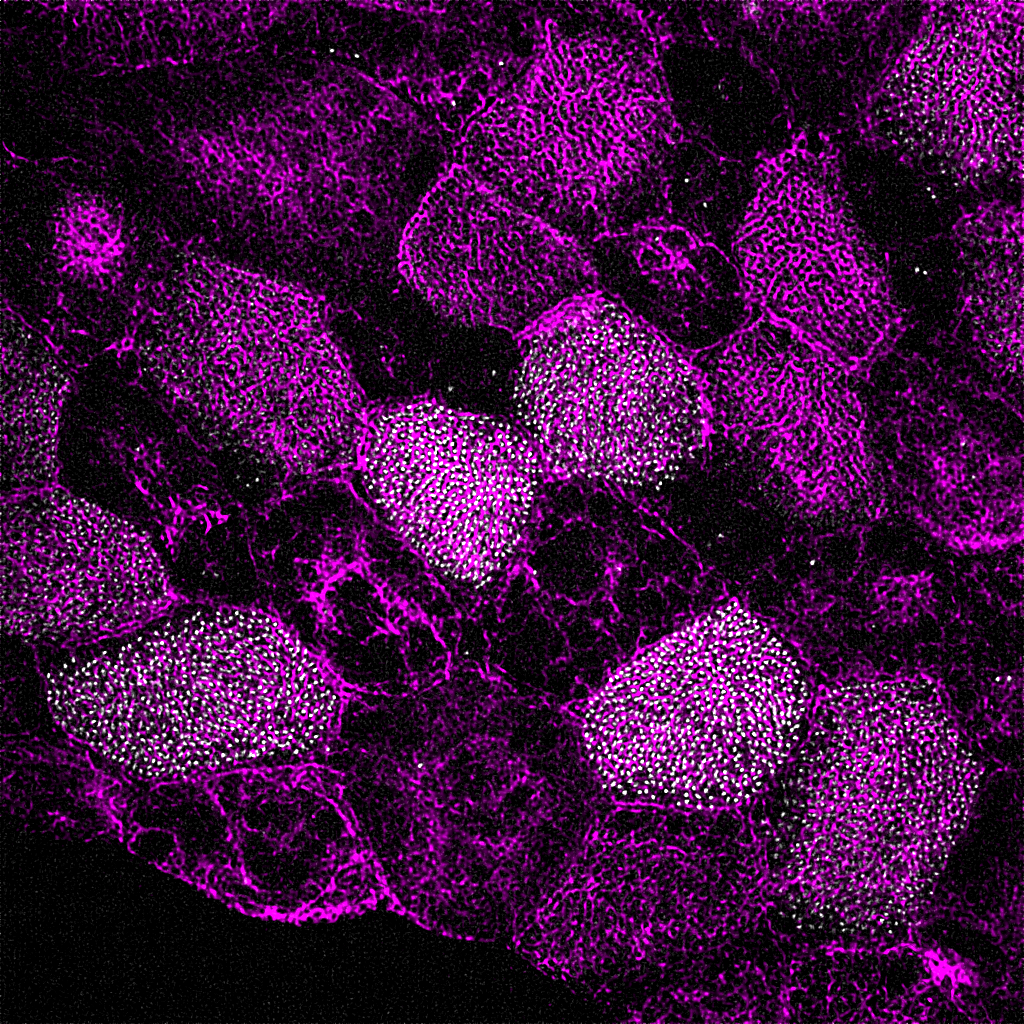

Supplement: Supplementary file 17 — Source Data Fig. 5 [file 44319_2024_66_MOESM17_ESM.zip › Source_Data_Figure_5/5C_MTEC_keratin8/5C_keratin8_Late_TP_1_Large.tif]

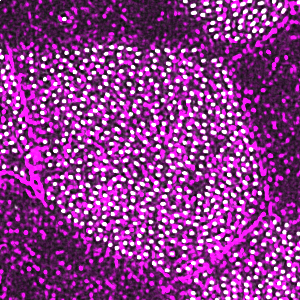

Supplement: Supplementary file 17 — Source Data Fig. 5 [file 44319_2024_66_MOESM17_ESM.zip › Source_Data_Figure_5/5C_MTEC_keratin8/5C_keratin8_Late_TP_2.tif]

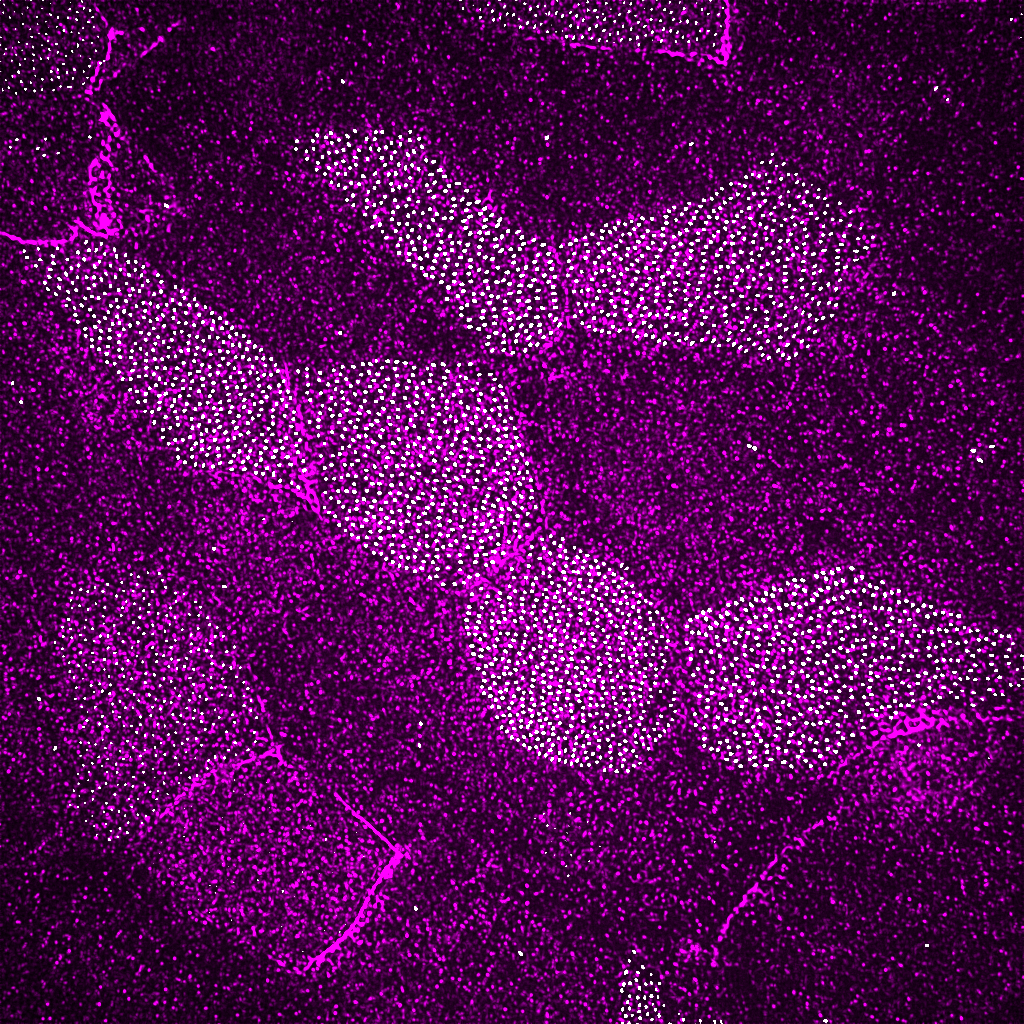

Supplement: Supplementary file 17 — Source Data Fig. 5 [file 44319_2024_66_MOESM17_ESM.zip › Source_Data_Figure_5/5C_MTEC_keratin8/5C_keratin8_Late_TP_2_Large.tif]

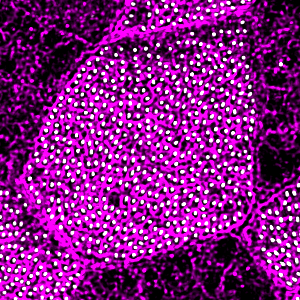

Supplement: Supplementary file 17 — Source Data Fig. 5 [file 44319_2024_66_MOESM17_ESM.zip › Source_Data_Figure_5/5C_MTEC_keratin8/5C_keratin8_Late_TP_2_rotated.tif]

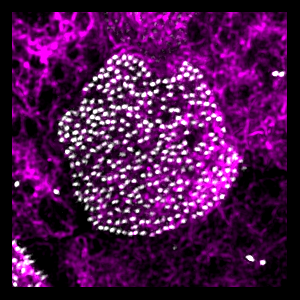

Supplement: Supplementary file 17 — Source Data Fig. 5 [file 44319_2024_66_MOESM17_ESM.zip › Source_Data_Figure_5/5E-F(S3A-B)_keratin8_Analysis/5E-F_keratin8_Early_TP_Analysis(Cell_1-18).tif]

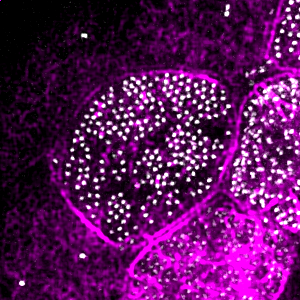

Supplement: Supplementary file 17 — Source Data Fig. 5 [file 44319_2024_66_MOESM17_ESM.zip › Source_Data_Figure_5/5E-F(S3A-B)_keratin8_Analysis/5E-F_keratin8_Initial_TP_Analysis(Cell_1-11).tif]

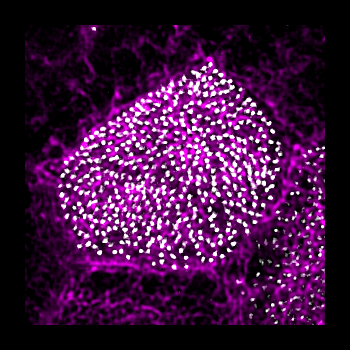

Supplement: Supplementary file 17 — Source Data Fig. 5 [file 44319_2024_66_MOESM17_ESM.zip › Source_Data_Figure_5/5E-F(S3A-B)_keratin8_Analysis/5E-F_keratin8_Late_TP_Analysis(Cell_1-21).tif]

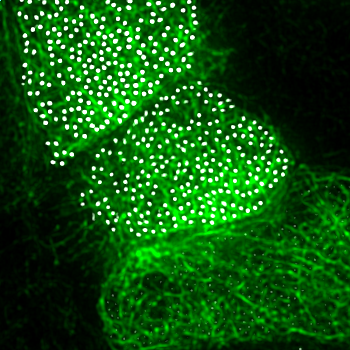

Supplement: Supplementary file 17 — Source Data Fig. 5 [file 44319_2024_66_MOESM17_ESM.zip › Source_Data_Figure_5/5E-F_tubulin_Analysis/5E-F_tubulin_Early_TP_Analysis(Cell_1-15).tif]

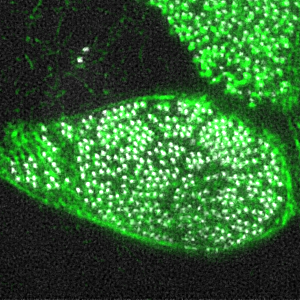

Supplement: Supplementary file 17 — Source Data Fig. 5 [file 44319_2024_66_MOESM17_ESM.zip › Source_Data_Figure_5/5E-F_tubulin_Analysis/5E-F_tubulin_Initial_TP_Analysis(Cell_1-15).tif]

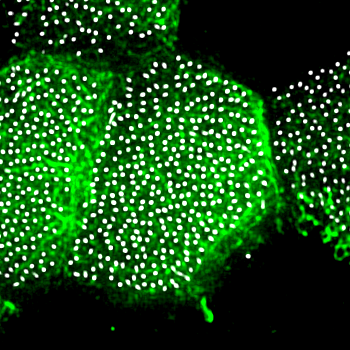

Supplement: Supplementary file 17 — Source Data Fig. 5 [file 44319_2024_66_MOESM17_ESM.zip › Source_Data_Figure_5/5E-F_tubulin_Analysis/5E-F_tubulin_Late_TP_Analysis(Cell_1-23).tif]

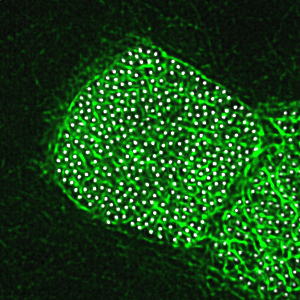

Supplement: Supplementary file 18 — Source Data Fig. 6 [file 44319_2024_66_MOESM18_ESM.zip › Source_Data_Figure_6/6A_MTEC_tubulin/6A_tubulin_Early_TP_DMSO.tif]

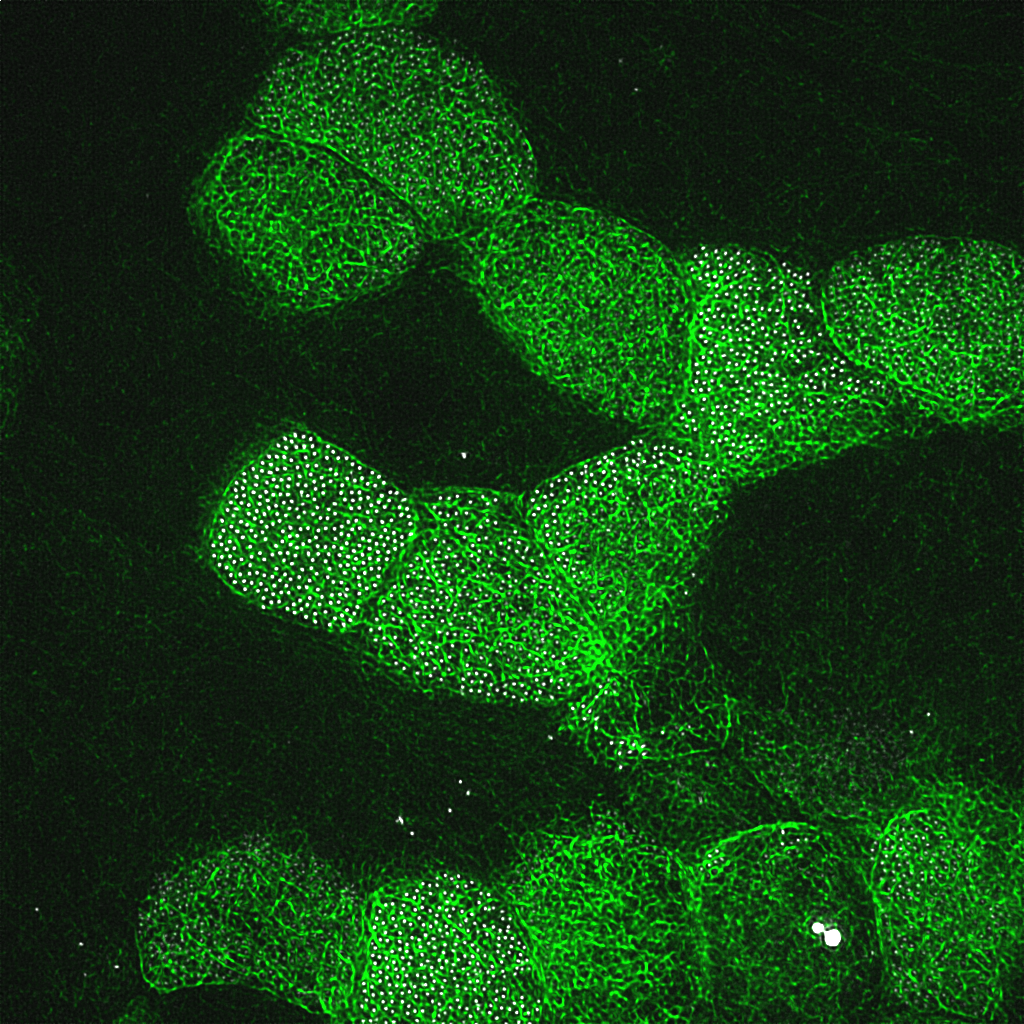

Supplement: Supplementary file 18 — Source Data Fig. 6 [file 44319_2024_66_MOESM18_ESM.zip › Source_Data_Figure_6/6A_MTEC_tubulin/6A_tubulin_Early_TP_DMSO_Large.tif]

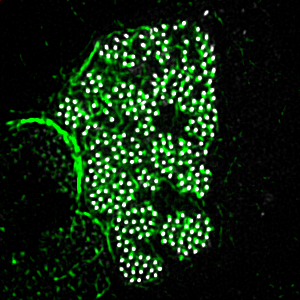

Supplement: Supplementary file 18 — Source Data Fig. 6 [file 44319_2024_66_MOESM18_ESM.zip › Source_Data_Figure_6/6A_MTEC_tubulin/6A_tubulin_Early_TP_Noc.tif]

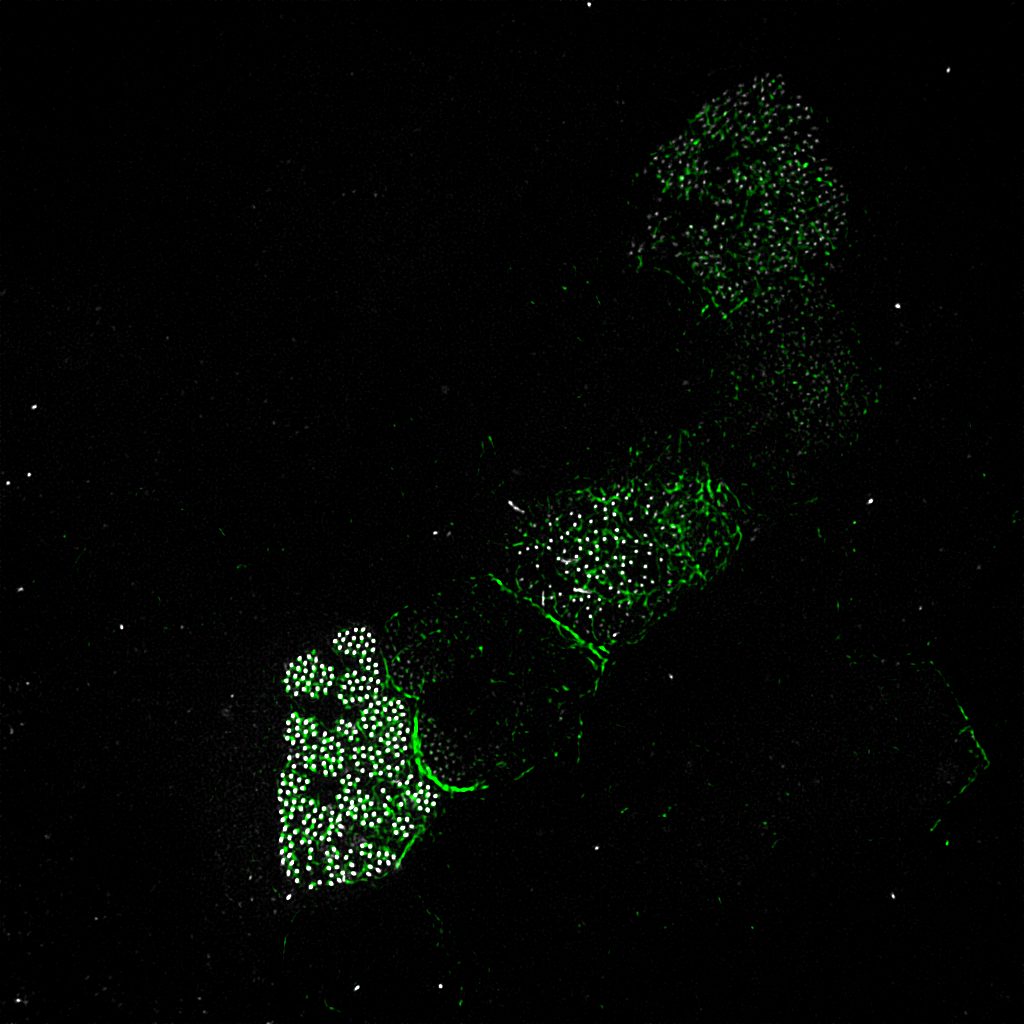

Supplement: Supplementary file 18 — Source Data Fig. 6 [file 44319_2024_66_MOESM18_ESM.zip › Source_Data_Figure_6/6A_MTEC_tubulin/6A_tubulin_Early_TP_Noc_Large.tif]

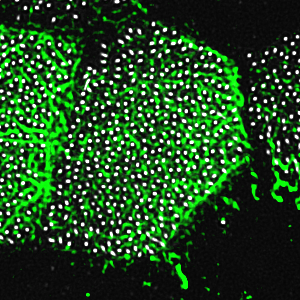

Supplement: Supplementary file 18 — Source Data Fig. 6 [file 44319_2024_66_MOESM18_ESM.zip › Source_Data_Figure_6/6A_MTEC_tubulin/6A_tubulin_Late_TP_DMSO.tif]

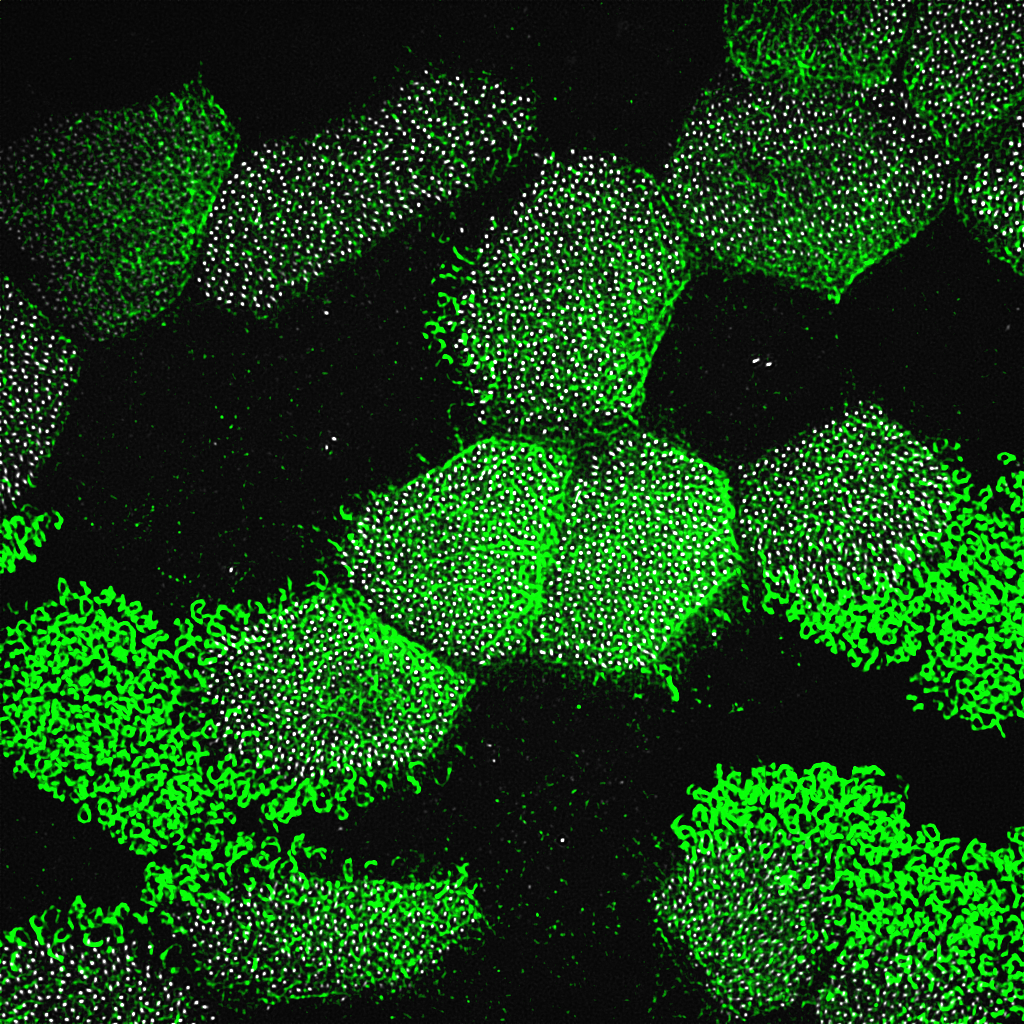

Supplement: Supplementary file 18 — Source Data Fig. 6 [file 44319_2024_66_MOESM18_ESM.zip › Source_Data_Figure_6/6A_MTEC_tubulin/6A_tubulin_Late_TP_DMSO_Large.tif]

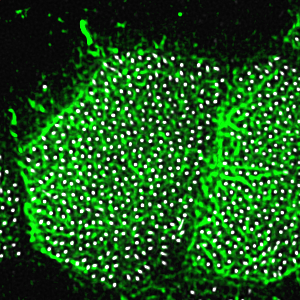

Supplement: Supplementary file 18 — Source Data Fig. 6 [file 44319_2024_66_MOESM18_ESM.zip › Source_Data_Figure_6/6A_MTEC_tubulin/6A_tubulin_Late_TP_DMSO_rotated.tif]

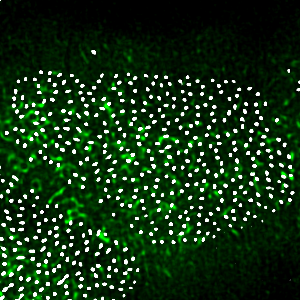

Supplement: Supplementary file 18 — Source Data Fig. 6 [file 44319_2024_66_MOESM18_ESM.zip › Source_Data_Figure_6/6A_MTEC_tubulin/6A_tubulin_Late_TP_Noc.tif]

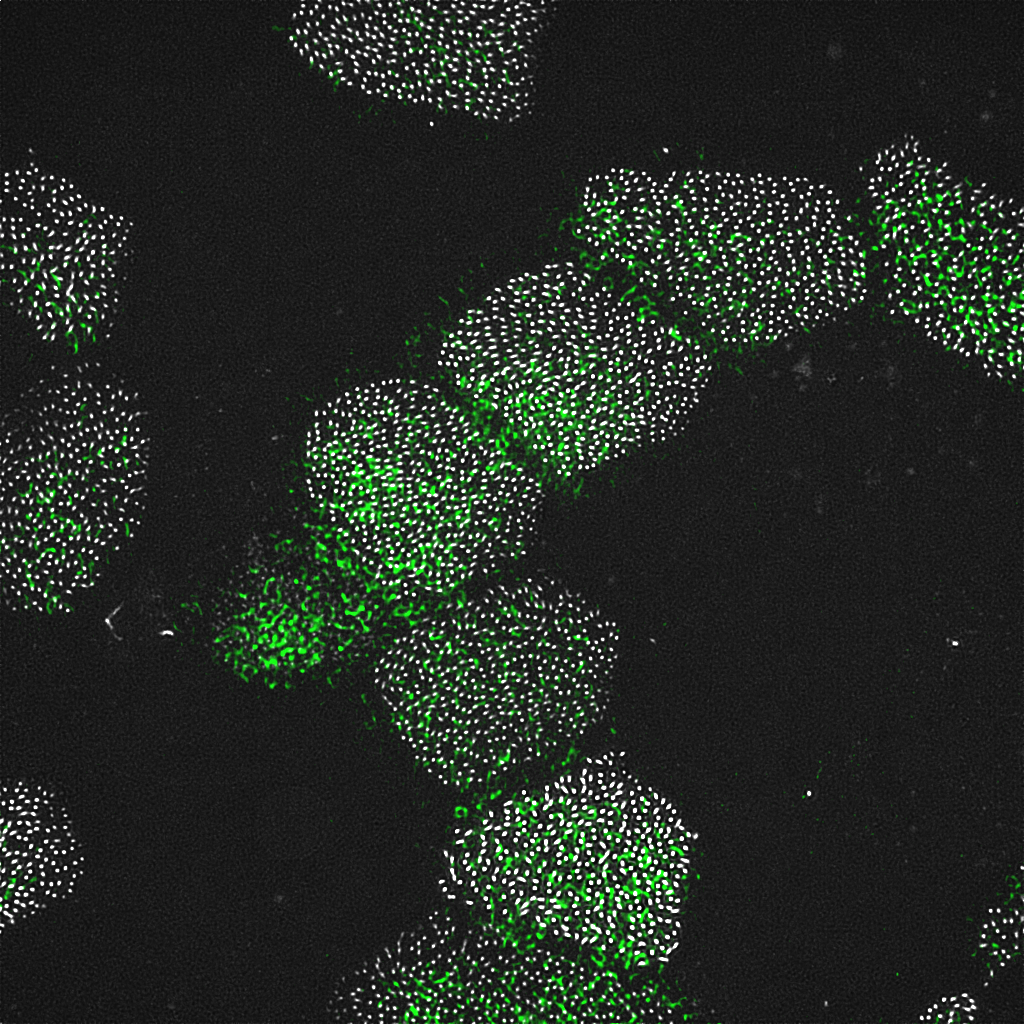

Supplement: Supplementary file 18 — Source Data Fig. 6 [file 44319_2024_66_MOESM18_ESM.zip › Source_Data_Figure_6/6A_MTEC_tubulin/6A_tubulin_Late_TP_Noc_Large.tif]

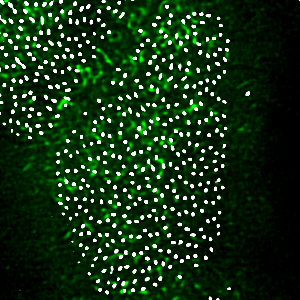

Supplement: Supplementary file 18 — Source Data Fig. 6 [file 44319_2024_66_MOESM18_ESM.zip › Source_Data_Figure_6/6A_MTEC_tubulin/6A_tubulin_Late_TP_Noc_rotated.tif]

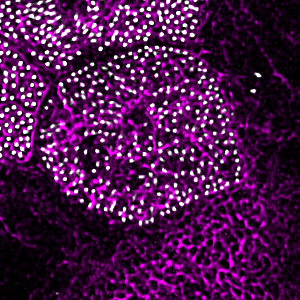

Supplement: Supplementary file 18 — Source Data Fig. 6 [file 44319_2024_66_MOESM18_ESM.zip › Source_Data_Figure_6/6B_MTEC_keratin8/6B_keratin8_Early_TP_DMSO.tif]

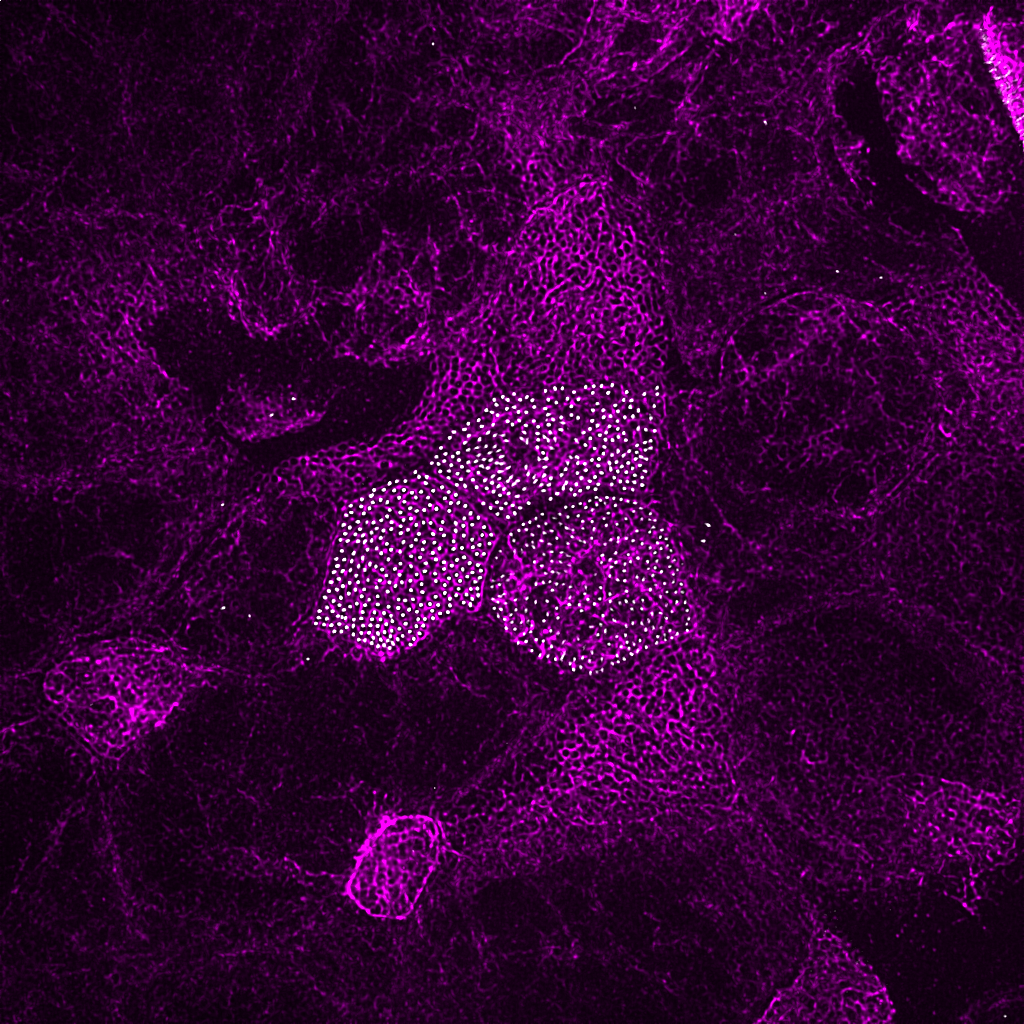

Supplement: Supplementary file 18 — Source Data Fig. 6 [file 44319_2024_66_MOESM18_ESM.zip › Source_Data_Figure_6/6B_MTEC_keratin8/6B_keratin8_Early_TP_DMSO_Large.tif]

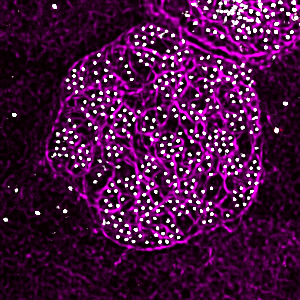

Supplement: Supplementary file 18 — Source Data Fig. 6 [file 44319_2024_66_MOESM18_ESM.zip › Source_Data_Figure_6/6B_MTEC_keratin8/6B_keratin8_Early_TP_Noc.tif]

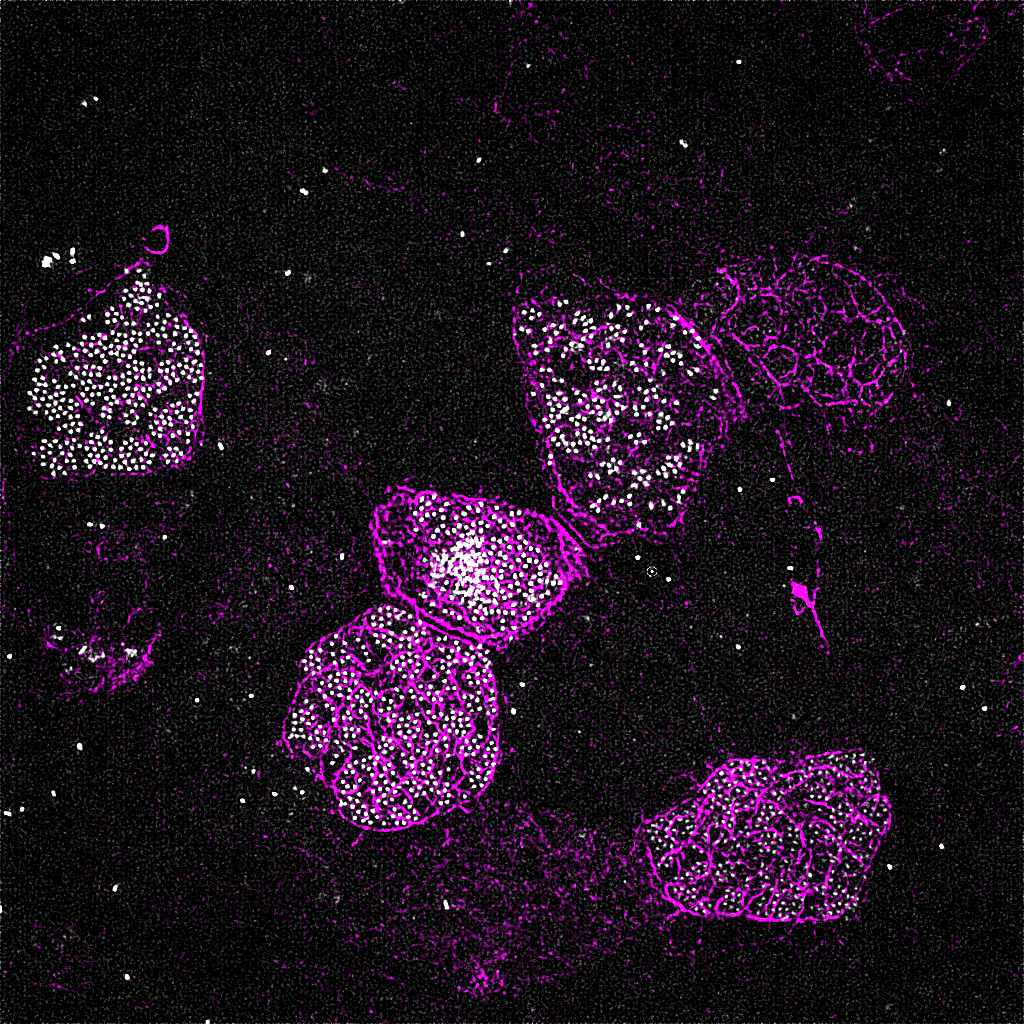

Supplement: Supplementary file 18 — Source Data Fig. 6 [file 44319_2024_66_MOESM18_ESM.zip › Source_Data_Figure_6/6B_MTEC_keratin8/6B_keratin8_Early_TP_Noc_Large.tif]

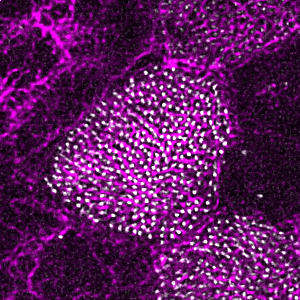

Supplement: Supplementary file 18 — Source Data Fig. 6 [file 44319_2024_66_MOESM18_ESM.zip › Source_Data_Figure_6/6B_MTEC_keratin8/6B_keratin8_Late_TP_DMSO.tif]

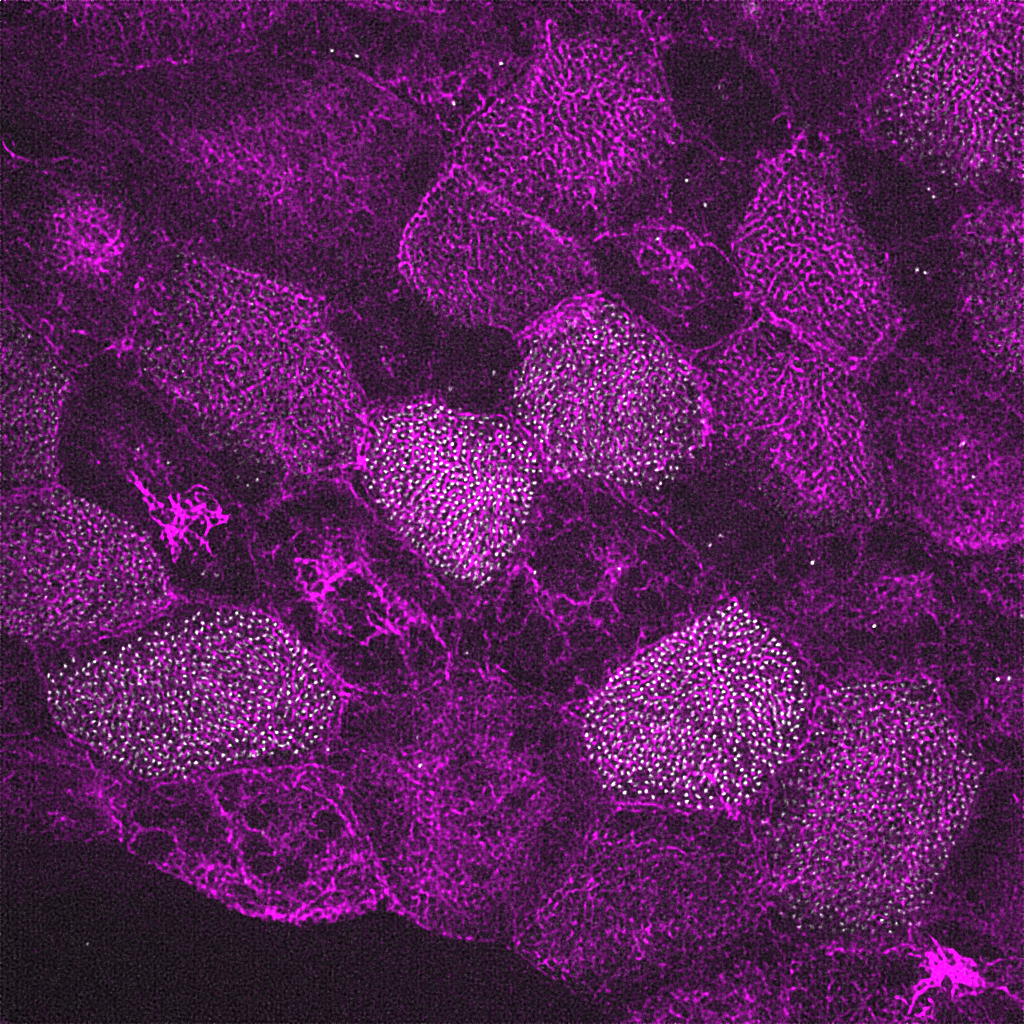

Supplement: Supplementary file 18 — Source Data Fig. 6 [file 44319_2024_66_MOESM18_ESM.zip › Source_Data_Figure_6/6B_MTEC_keratin8/6B_keratin8_Late_TP_DMSO_Large.tif]

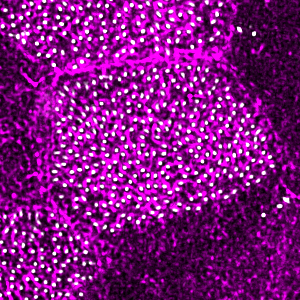

Supplement: Supplementary file 18 — Source Data Fig. 6 [file 44319_2024_66_MOESM18_ESM.zip › Source_Data_Figure_6/6B_MTEC_keratin8/6B_keratin8_Late_TP_Noc.tif]

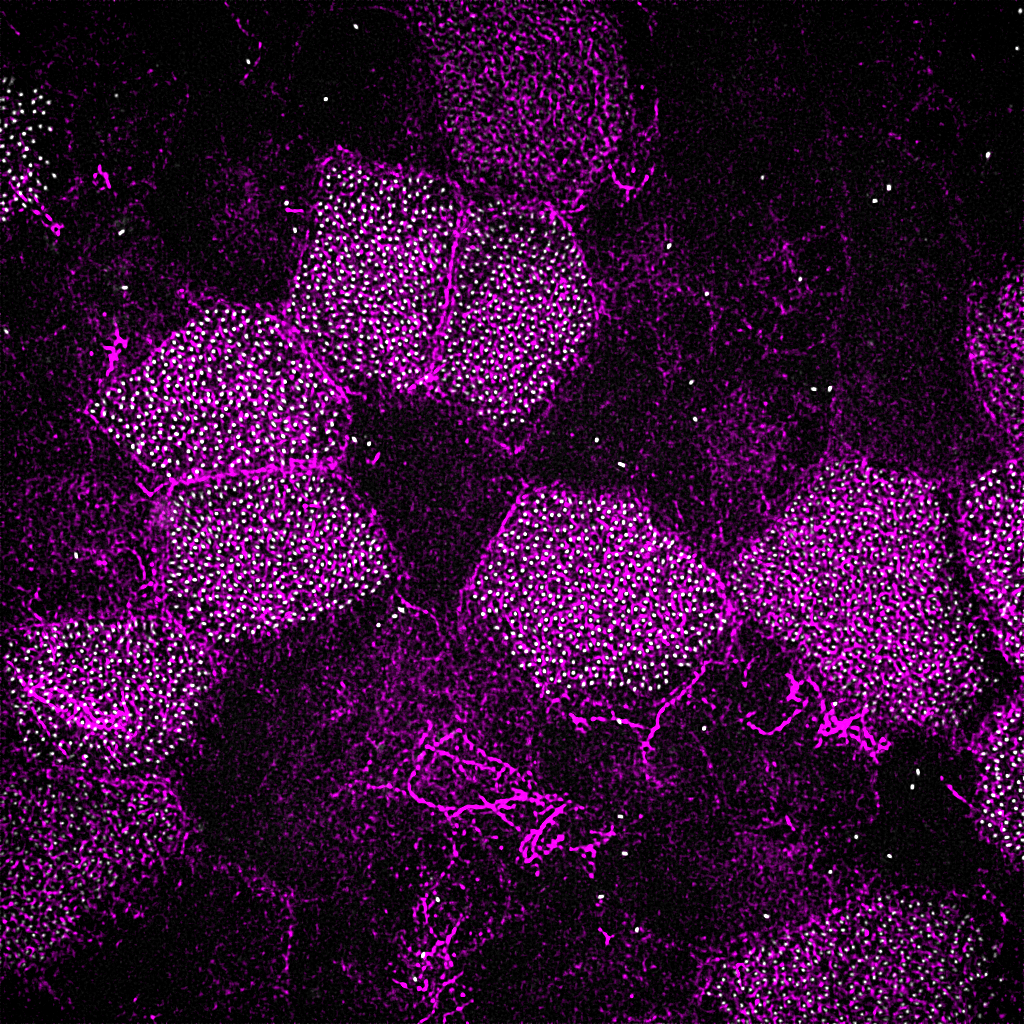

Supplement: Supplementary file 18 — Source Data Fig. 6 [file 44319_2024_66_MOESM18_ESM.zip › Source_Data_Figure_6/6B_MTEC_keratin8/6B_keratin8_Late_TP_Noc_Large.tif]

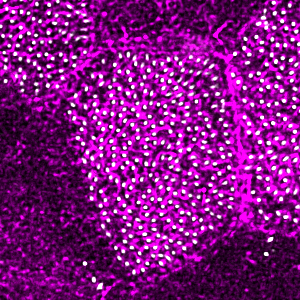

Supplement: Supplementary file 18 — Source Data Fig. 6 [file 44319_2024_66_MOESM18_ESM.zip › Source_Data_Figure_6/6B_MTEC_keratin8/6B_keratin8_Late_TP_Noc_rotated.tif]

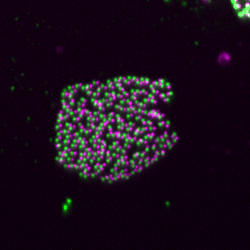

Supplement: Supplementary file 18 — Source Data Fig. 6 [file 44319_2024_66_MOESM18_ESM.zip › Source_Data_Figure_6/6C_MTEC_BBBF/6C_BBBF_Early_TP_DMSO.tif]

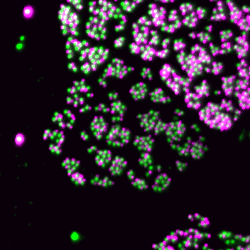

Supplement: Supplementary file 18 — Source Data Fig. 6 [file 44319_2024_66_MOESM18_ESM.zip › Source_Data_Figure_6/6C_MTEC_BBBF/6C_BBBF_Early_TP_Noc.tif]

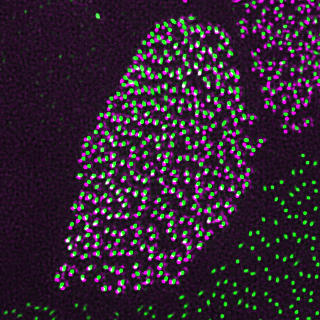

Supplement: Supplementary file 18 — Source Data Fig. 6 [file 44319_2024_66_MOESM18_ESM.zip › Source_Data_Figure_6/6C_MTEC_BBBF/6C_BBBF_Late_TP_DMSO.tif]

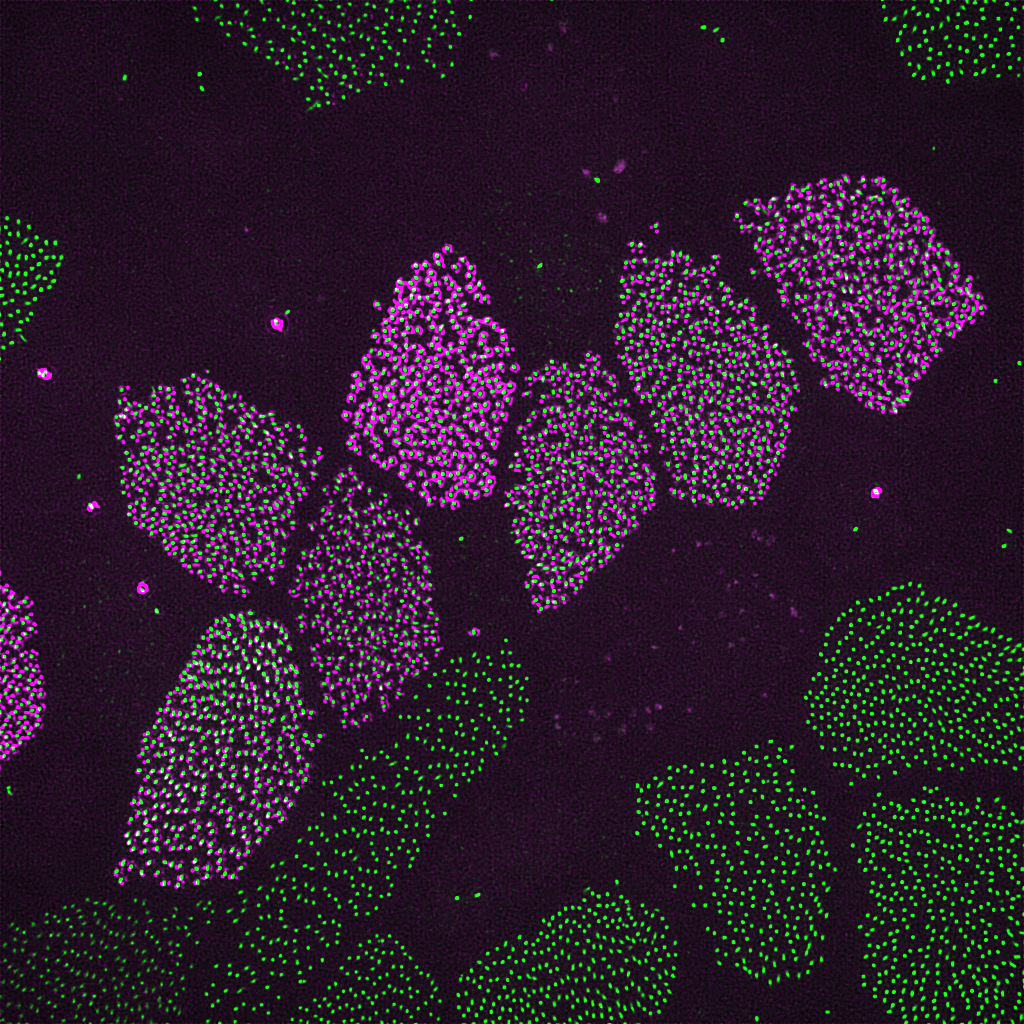

Supplement: Supplementary file 18 — Source Data Fig. 6 [file 44319_2024_66_MOESM18_ESM.zip › Source_Data_Figure_6/6C_MTEC_BBBF/6C_BBBF_Late_TP_DMSO_Large.tif]

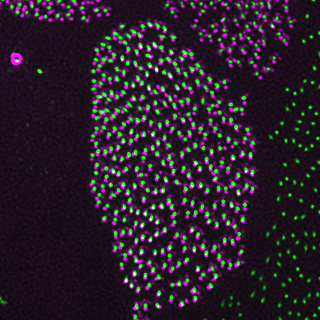

Supplement: Supplementary file 18 — Source Data Fig. 6 [file 44319_2024_66_MOESM18_ESM.zip › Source_Data_Figure_6/6C_MTEC_BBBF/6C_BBBF_Late_TP_DMSO_rotated.tif]

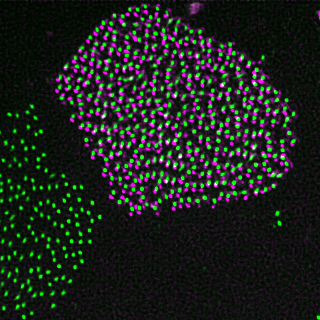

Supplement: Supplementary file 18 — Source Data Fig. 6 [file 44319_2024_66_MOESM18_ESM.zip › Source_Data_Figure_6/6C_MTEC_BBBF/6C_BBBF_Late_TP_Noc.tif]

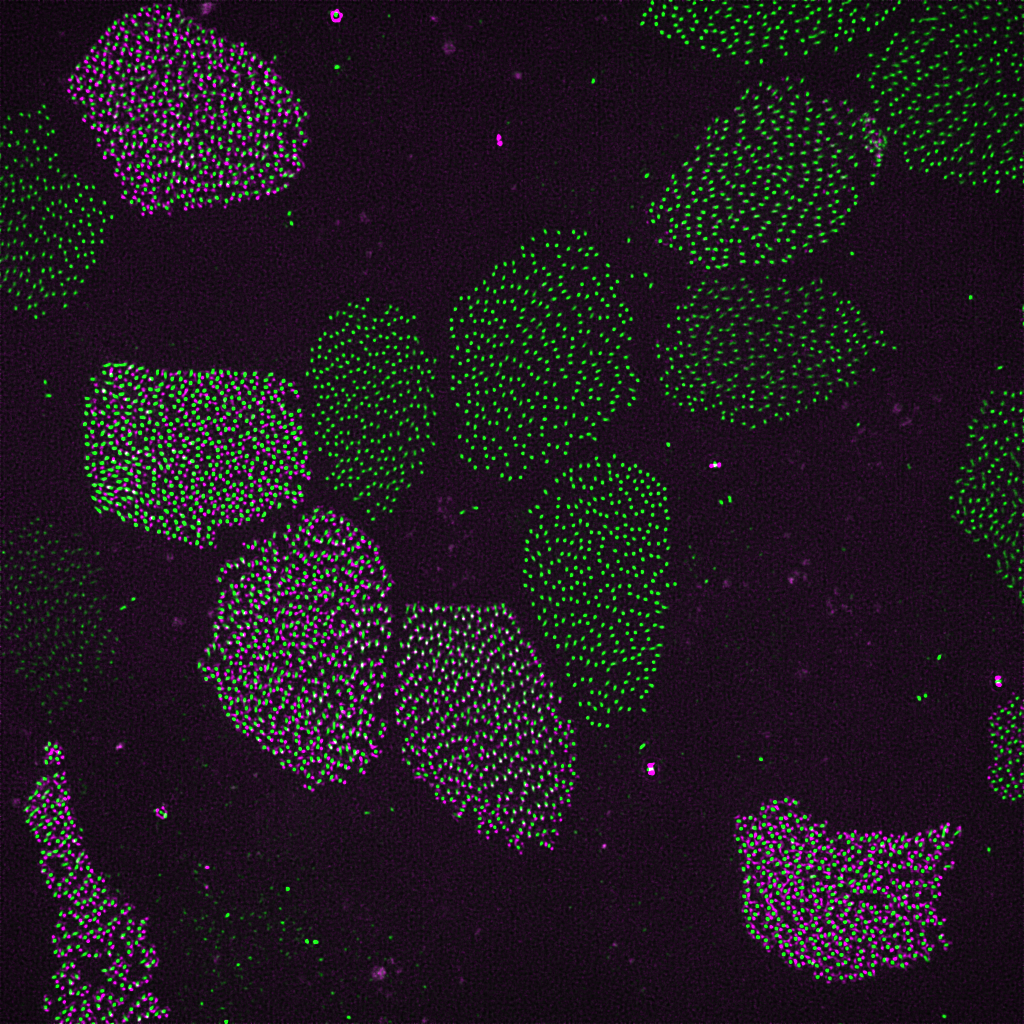

Supplement: Supplementary file 18 — Source Data Fig. 6 [file 44319_2024_66_MOESM18_ESM.zip › Source_Data_Figure_6/6C_MTEC_BBBF/6C_BBBF_Late_TP_Noc_Large.tif]

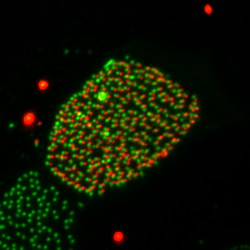

Supplement: Supplementary file 18 — Source Data Fig. 6 [file 44319_2024_66_MOESM18_ESM.zip › Source_Data_Figure_6/6C-E(EV3D)_Early_TP_Analysis/Early_TP_DMSO_Analysis(Cell_1-22).tif]

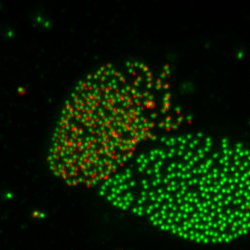

Supplement: Supplementary file 18 — Source Data Fig. 6 [file 44319_2024_66_MOESM18_ESM.zip › Source_Data_Figure_6/6C-E(EV3D)_Early_TP_Analysis/Early_TP_Noc_Analysis(Cell_1-24).tif]

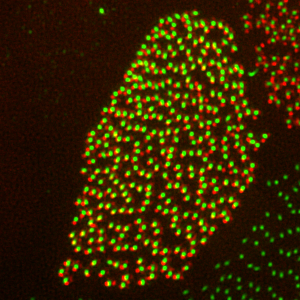

Supplement: Supplementary file 18 — Source Data Fig. 6 [file 44319_2024_66_MOESM18_ESM.zip › Source_Data_Figure_6/6C-E(EV3D)_Late_TP_Analysis/Late_TP_DMSO_Analysis(Cell_1-18).tif]

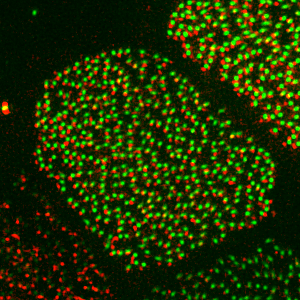

Supplement: Supplementary file 18 — Source Data Fig. 6 [file 44319_2024_66_MOESM18_ESM.zip › Source_Data_Figure_6/6C-E(EV3D)_Late_TP_Analysis/Late_TP_Noc_Analysis(Cell_1-17).tif]

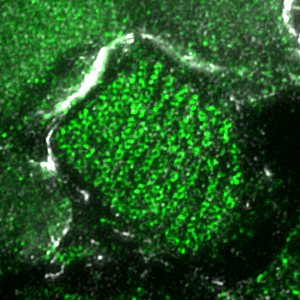

Supplement: Supplementary file 19 — Source Data Fig. 7 [file 44319_2024_66_MOESM19_ESM.zip › Source_Data_Figure_7/7B_Trachea/7B_KRT8-KO_trachea.tif]

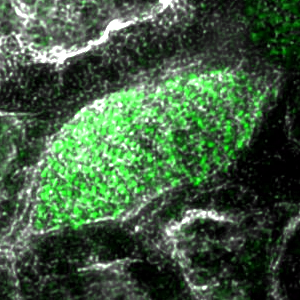

Supplement: Supplementary file 19 — Source Data Fig. 7 [file 44319_2024_66_MOESM19_ESM.zip › Source_Data_Figure_7/7B_Trachea/7B_WT_trachea.tif]

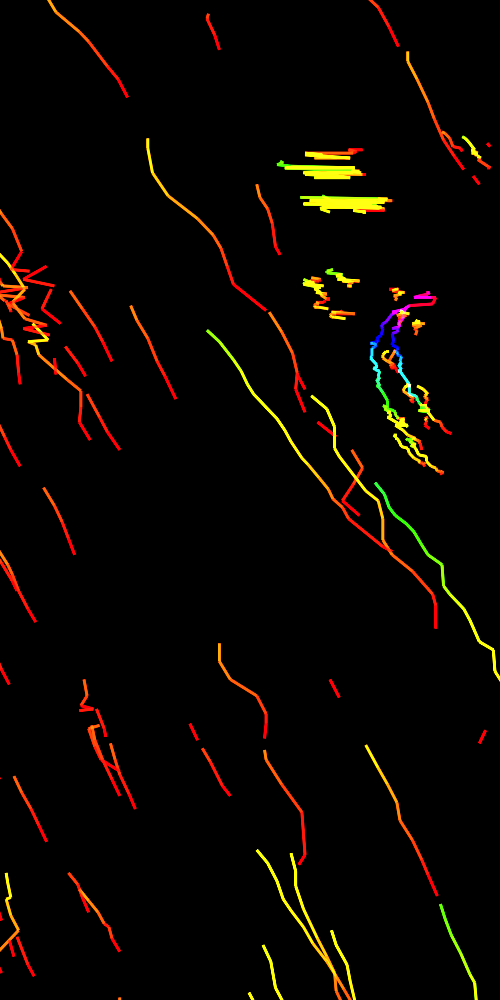

Supplement: Supplementary file 19 — Source Data Fig. 7 [file 44319_2024_66_MOESM19_ESM.zip › Source_Data_Figure_7/7C(EV4A)_mucociliary_clearance/7C_KRT8-KO_mucociliary_clearance.tif]

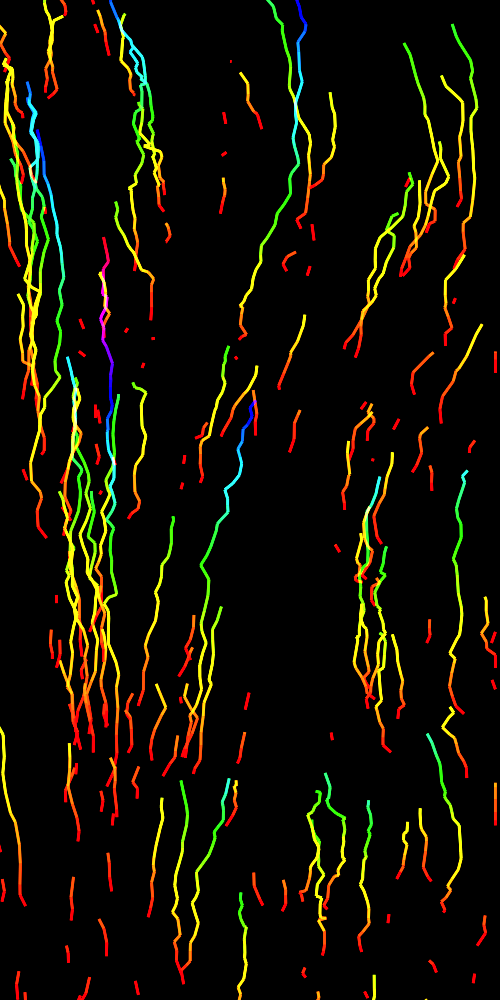

Supplement: Supplementary file 19 — Source Data Fig. 7 [file 44319_2024_66_MOESM19_ESM.zip › Source_Data_Figure_7/7C(EV4A)_mucociliary_clearance/7C_WT_mucociliary_clearance.tif]

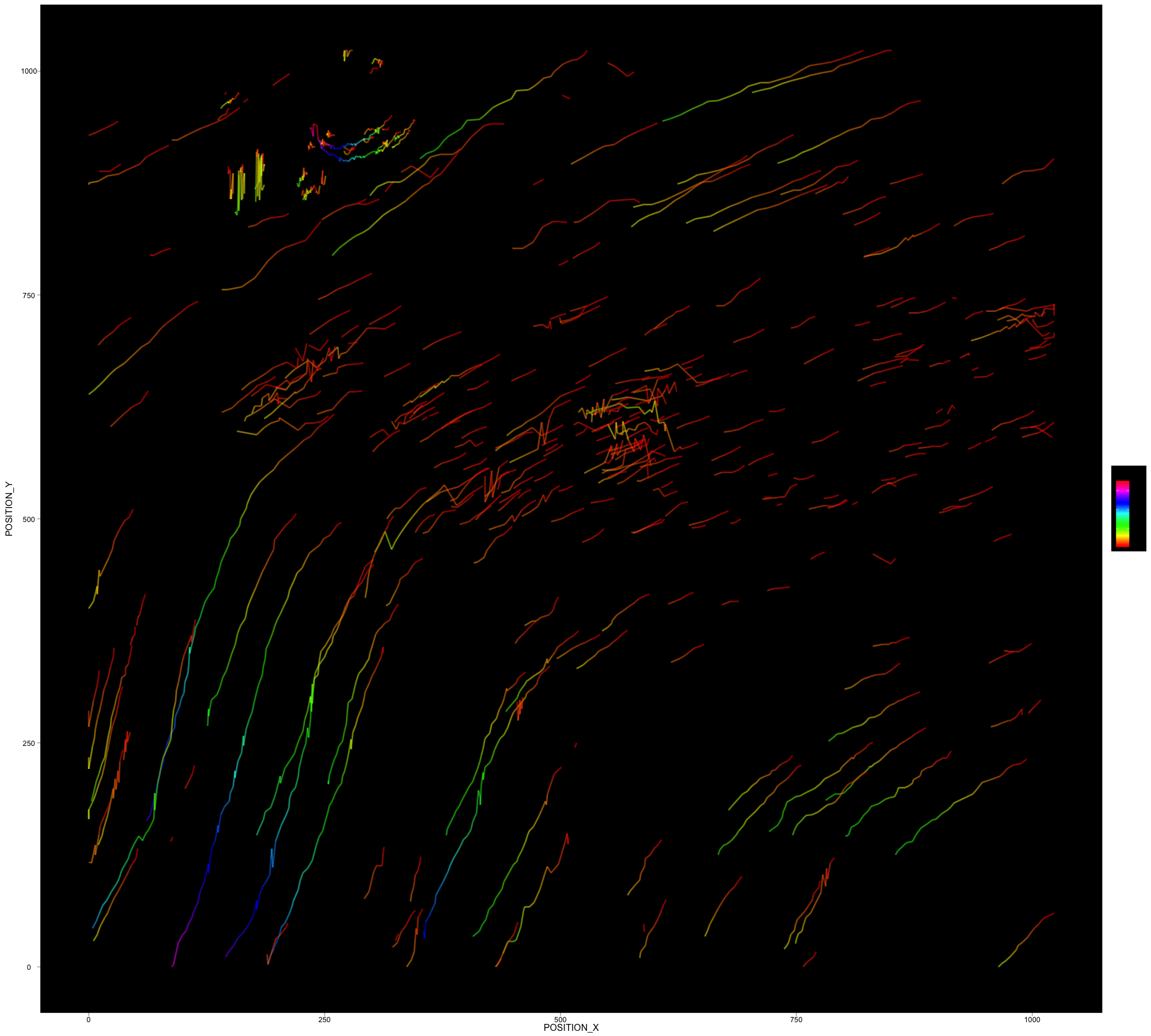

Supplement: Supplementary file 19 — Source Data Fig. 7 [file 44319_2024_66_MOESM19_ESM.zip › Source_Data_Figure_7/7C(EV4A)_mucociliary_clearance/EV4A_KRT8-KO_mucociliary_clearance_Large.tif]

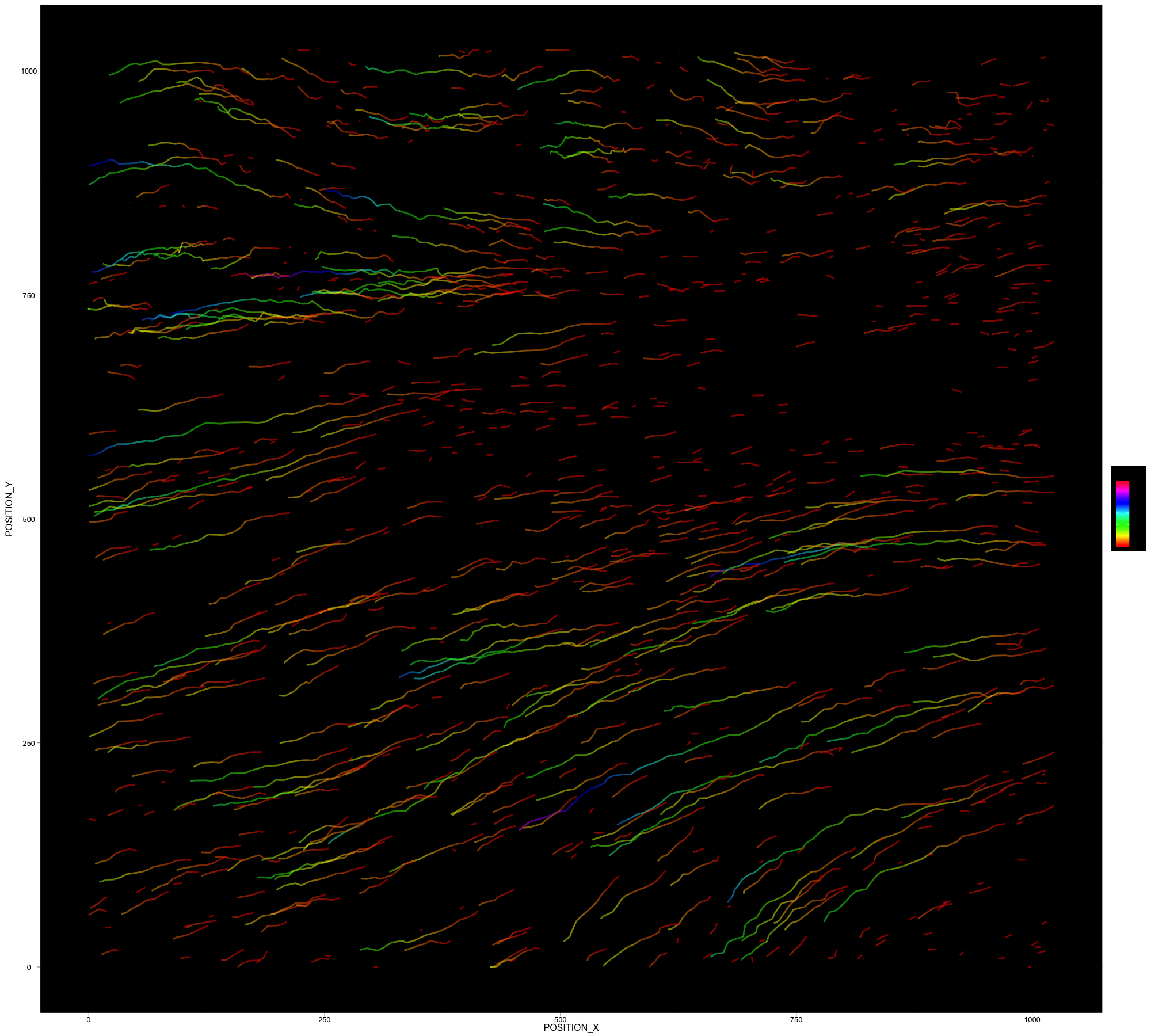

Supplement: Supplementary file 19 — Source Data Fig. 7 [file 44319_2024_66_MOESM19_ESM.zip › Source_Data_Figure_7/7C(EV4A)_mucociliary_clearance/EV4A_WT_mucociliary_clearance_Large.tif]
