# Supplementary figures and images for: Dual-color live imaging unveils stepwise organization of multiple basal body arrays by cytoskeletons (part 2 of 2)
Source: EMBO Rep. 2024 Feb 5;25(3):1176–207. doi: 10.1038/s44319-024-00066-0 (PMC10933483; doi:10.1038/s44319-024-00066-0)

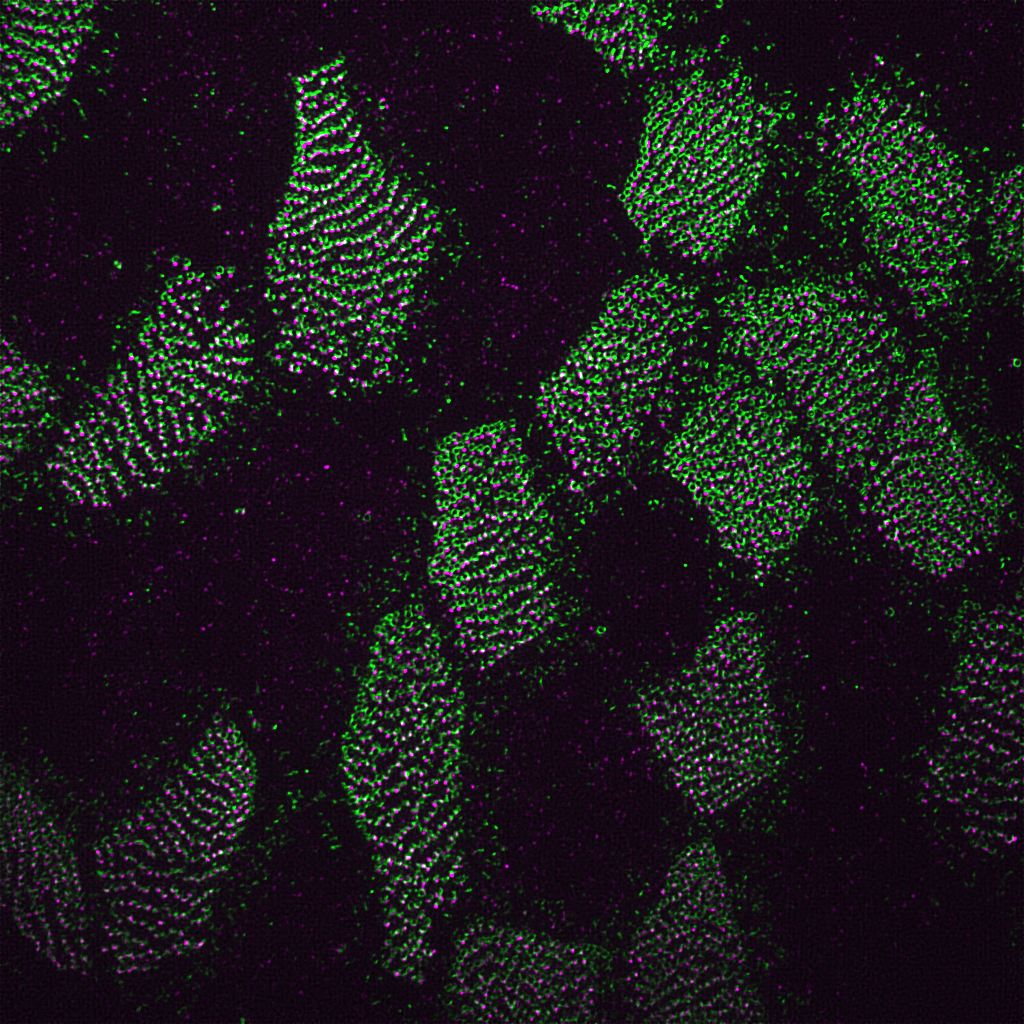

Supplement: Supplementary file 19 — Source Data Fig. 7 [file 44319_2024_66_MOESM19_ESM.zip › Source_Data_Figure_7/7E(F_and_G)_trachea/7E(F_and_G)_WT_trachea_Large.tif]

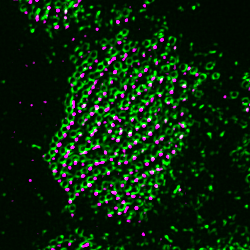

Supplement: Supplementary file 19 — Source Data Fig. 7 [file 44319_2024_66_MOESM19_ESM.zip › Source_Data_Figure_7/7E(F_and_G)_trachea/7E_WT_trachea.tif]

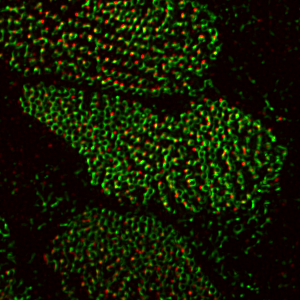

Supplement: Supplementary file 19 — Source Data Fig. 7 [file 44319_2024_66_MOESM19_ESM.zip › Source_Data_Figure_7/7E-I(EV4B-C)_trachea_Analysis/KRT8-KO_trachea_Analysis(Cell_1-45).tif]

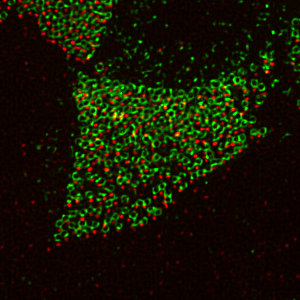

Supplement: Supplementary file 19 — Source Data Fig. 7 [file 44319_2024_66_MOESM19_ESM.zip › Source_Data_Figure_7/7E-I(EV4B-C)_trachea_Analysis/WT_trachea_Analysis(Cell_1-45).tif]

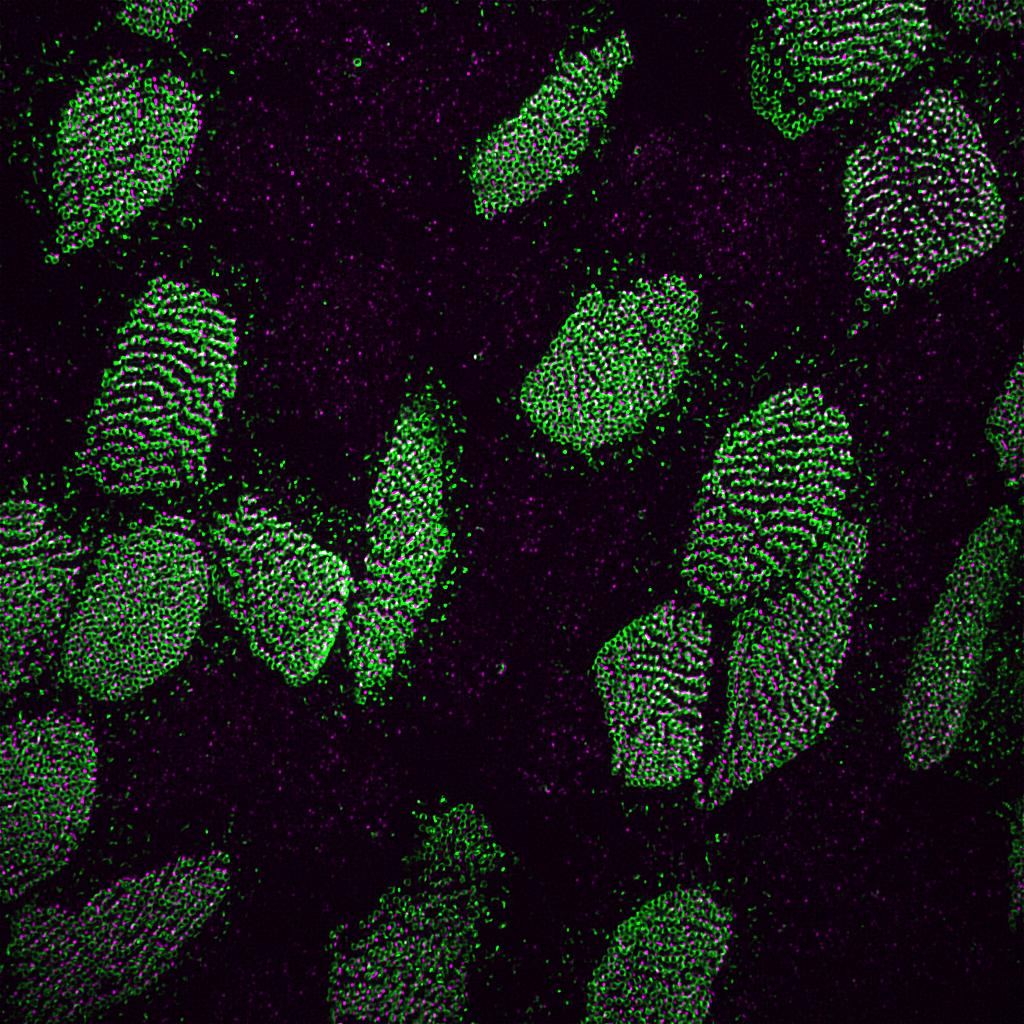

Supplement: Supplementary file 19 — Source Data Fig. 7 [file 44319_2024_66_MOESM19_ESM.zip › Source_Data_Figure_7/7F(E_and_G)_trachea/7F(G)_KRT8-KO_trachea_Large.tif]

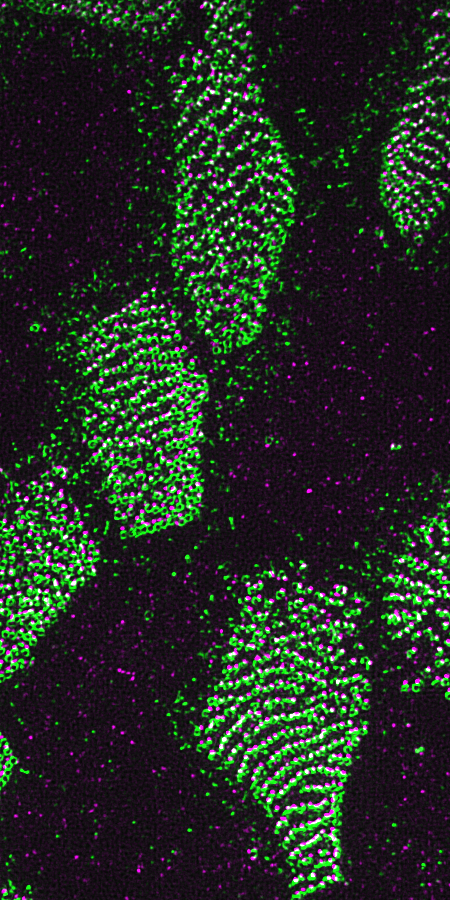

Supplement: Supplementary file 19 — Source Data Fig. 7 [file 44319_2024_66_MOESM19_ESM.zip › Source_Data_Figure_7/7F(E_and_G)_trachea/7F(G)_WT_trachea.tif]

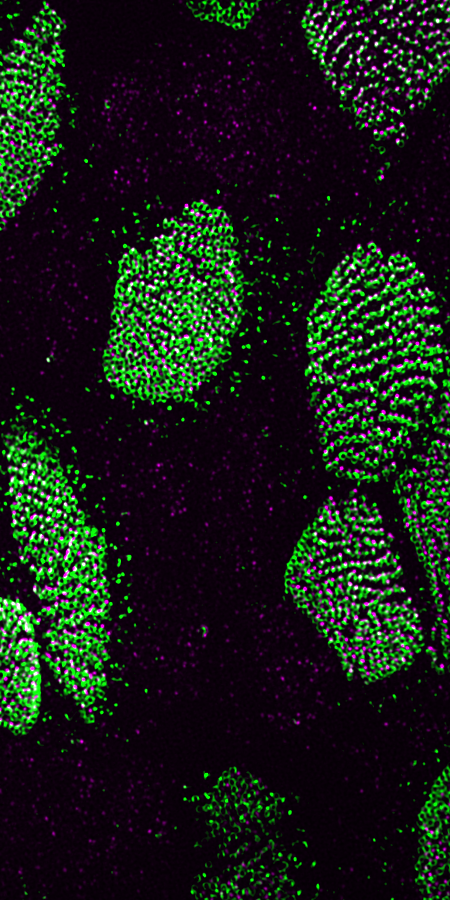

Supplement: Supplementary file 19 — Source Data Fig. 7 [file 44319_2024_66_MOESM19_ESM.zip › Source_Data_Figure_7/7F(E_and_G)_trachea/7F_KRT8-KO_trachea.tif]

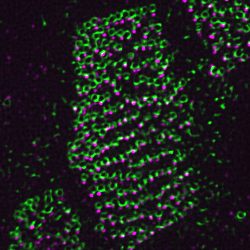

Supplement: Supplementary file 19 — Source Data Fig. 7 [file 44319_2024_66_MOESM19_ESM.zip › Source_Data_Figure_7/7G(E_and_F)_WT_trachea/7G(F)_WT_trachea_Stage5.tif]

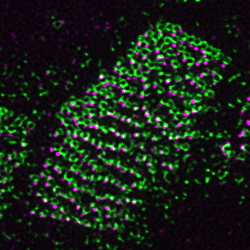

Supplement: Supplementary file 19 — Source Data Fig. 7 [file 44319_2024_66_MOESM19_ESM.zip › Source_Data_Figure_7/7G(E_and_F)_WT_trachea/7G(F)_WT_trachea_Stage5_rotated.tif]

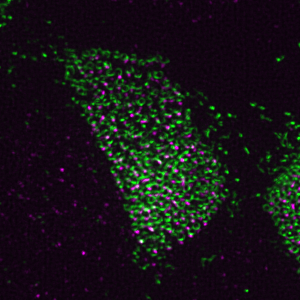

Supplement: Supplementary file 19 — Source Data Fig. 7 [file 44319_2024_66_MOESM19_ESM.zip › Source_Data_Figure_7/7G(E_and_F)_WT_trachea/7G_WT_trachea_Stage4.tif]

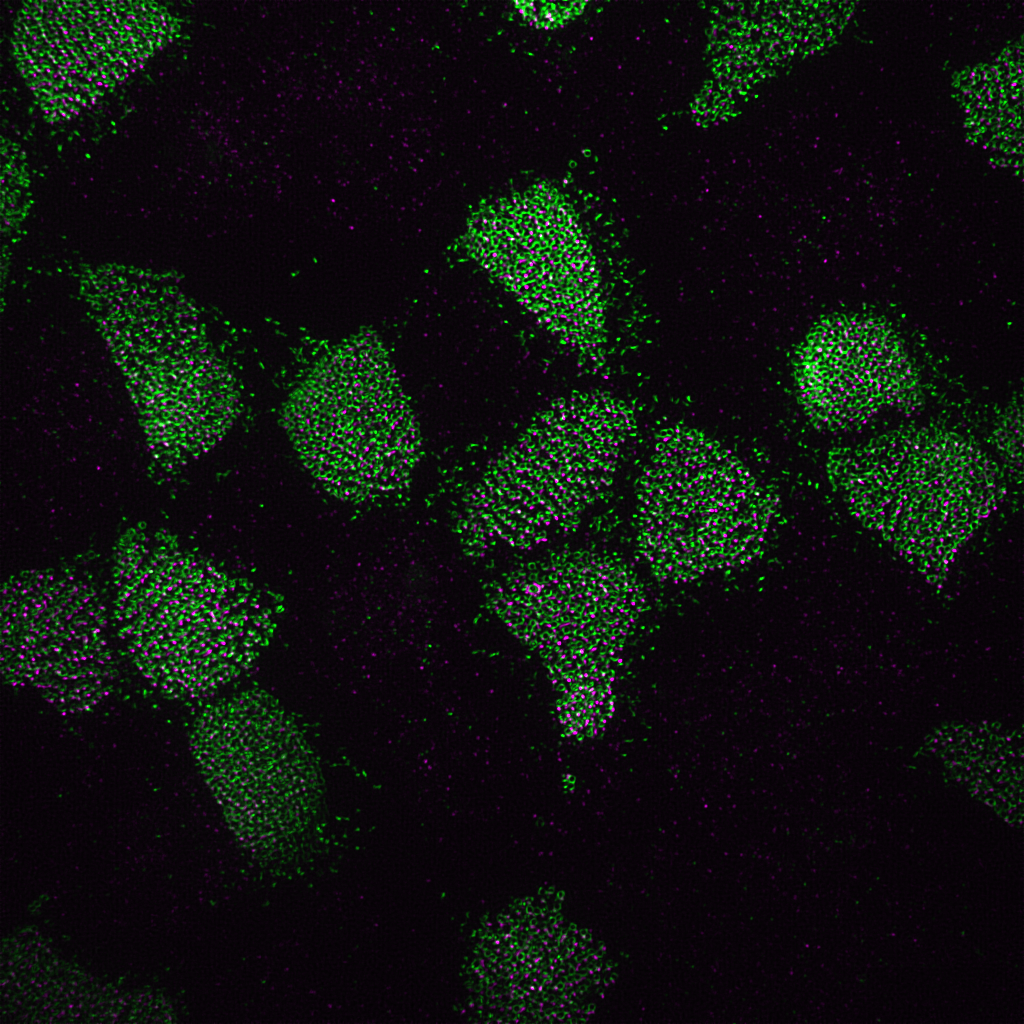

Supplement: Supplementary file 19 — Source Data Fig. 7 [file 44319_2024_66_MOESM19_ESM.zip › Source_Data_Figure_7/7G(E_and_F)_WT_trachea/7G_WT_trachea_Stage4_Large.tif]

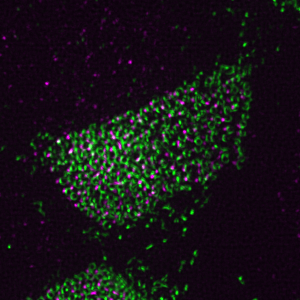

Supplement: Supplementary file 19 — Source Data Fig. 7 [file 44319_2024_66_MOESM19_ESM.zip › Source_Data_Figure_7/7G(E_and_F)_WT_trachea/7G_WT_trachea_Stage4_rotated.tif]

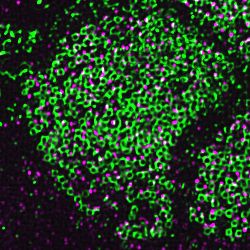

Supplement: Supplementary file 19 — Source Data Fig. 7 [file 44319_2024_66_MOESM19_ESM.zip › Source_Data_Figure_7/7G(F)_KRT8-KO_trachea/7G_KRT8-KO_trachea_Stage2-3.tif]

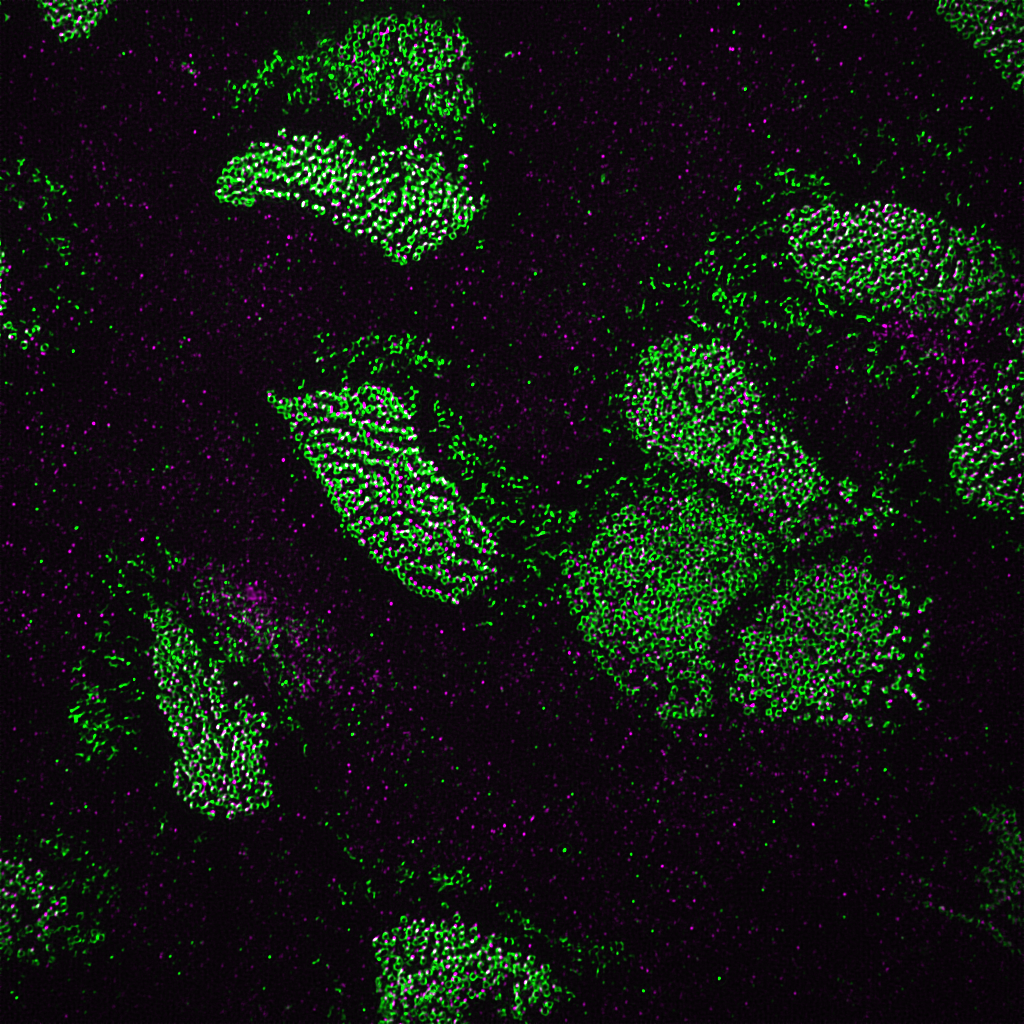

Supplement: Supplementary file 19 — Source Data Fig. 7 [file 44319_2024_66_MOESM19_ESM.zip › Source_Data_Figure_7/7G(F)_KRT8-KO_trachea/7G_KRT8-KO_trachea_Stage2-3_Large.tif]

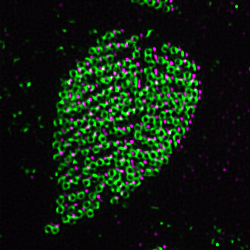

Supplement: Supplementary file 19 — Source Data Fig. 7 [file 44319_2024_66_MOESM19_ESM.zip › Source_Data_Figure_7/7G(F)_KRT8-KO_trachea/7G_KRT8-KO_trachea_Stage4.tif]

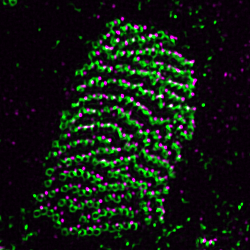

Supplement: Supplementary file 19 — Source Data Fig. 7 [file 44319_2024_66_MOESM19_ESM.zip › Source_Data_Figure_7/7G(F)_KRT8-KO_trachea/7G_KRT8-KO_trachea_Stage5.tif]

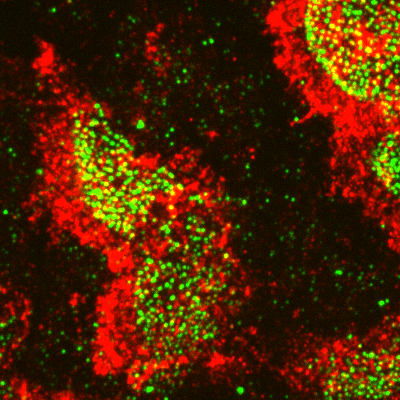

Supplement: Supplementary file 20 — Source Data Fig. 8 [file 44319_2024_66_MOESM20_ESM.zip › Source_Data_Figure_8/8B_trachea_tubulin/8B_KRT8-KO_trachea_DMSO_tub.tif]

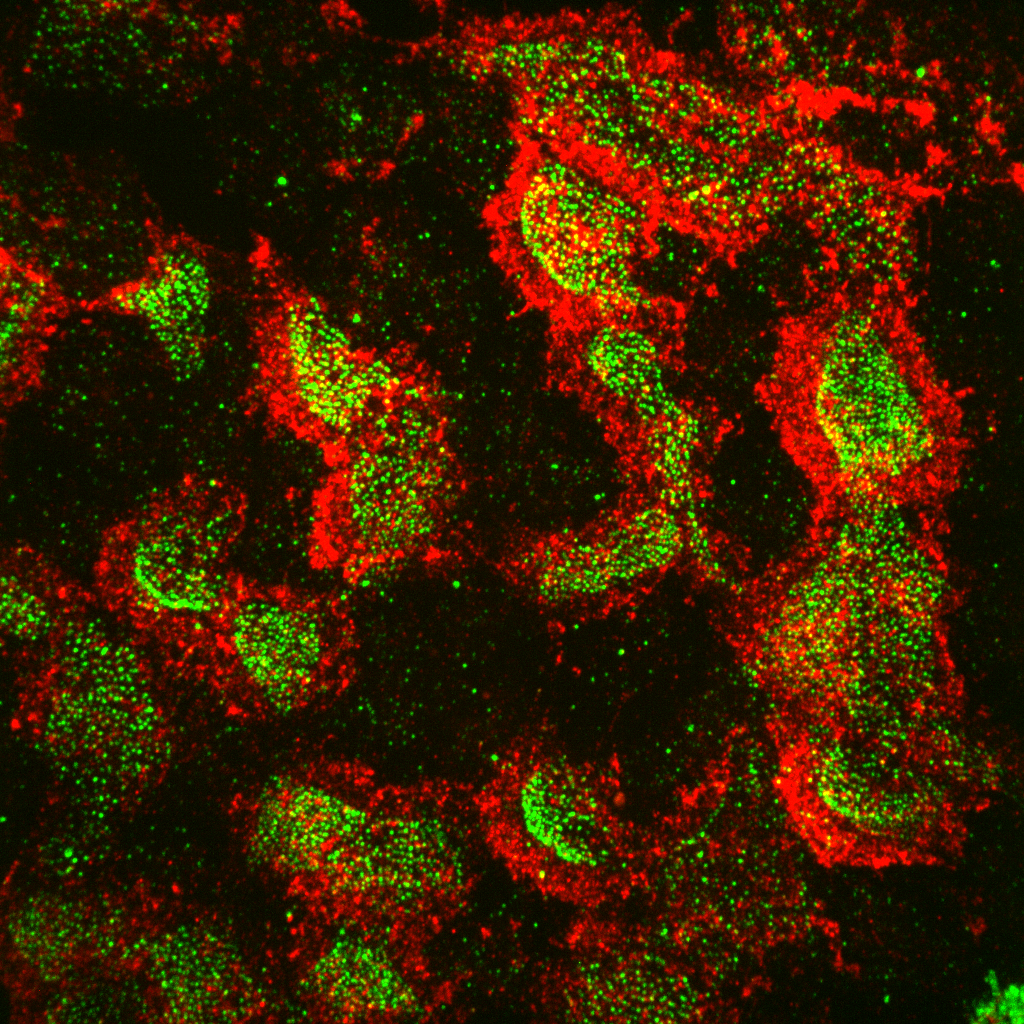

Supplement: Supplementary file 20 — Source Data Fig. 8 [file 44319_2024_66_MOESM20_ESM.zip › Source_Data_Figure_8/8B_trachea_tubulin/8B_KRT8-KO_trachea_DMSO_tub_Large.tif]

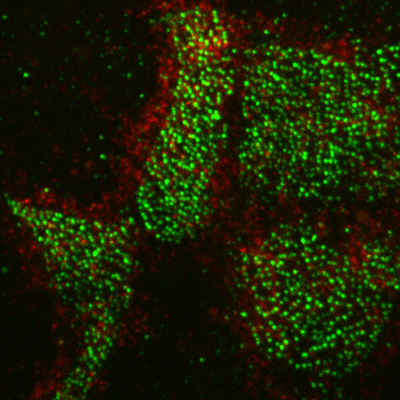

Supplement: Supplementary file 20 — Source Data Fig. 8 [file 44319_2024_66_MOESM20_ESM.zip › Source_Data_Figure_8/8B_trachea_tubulin/8B_KRT8-KO_trachea_Noc_tub.tif]

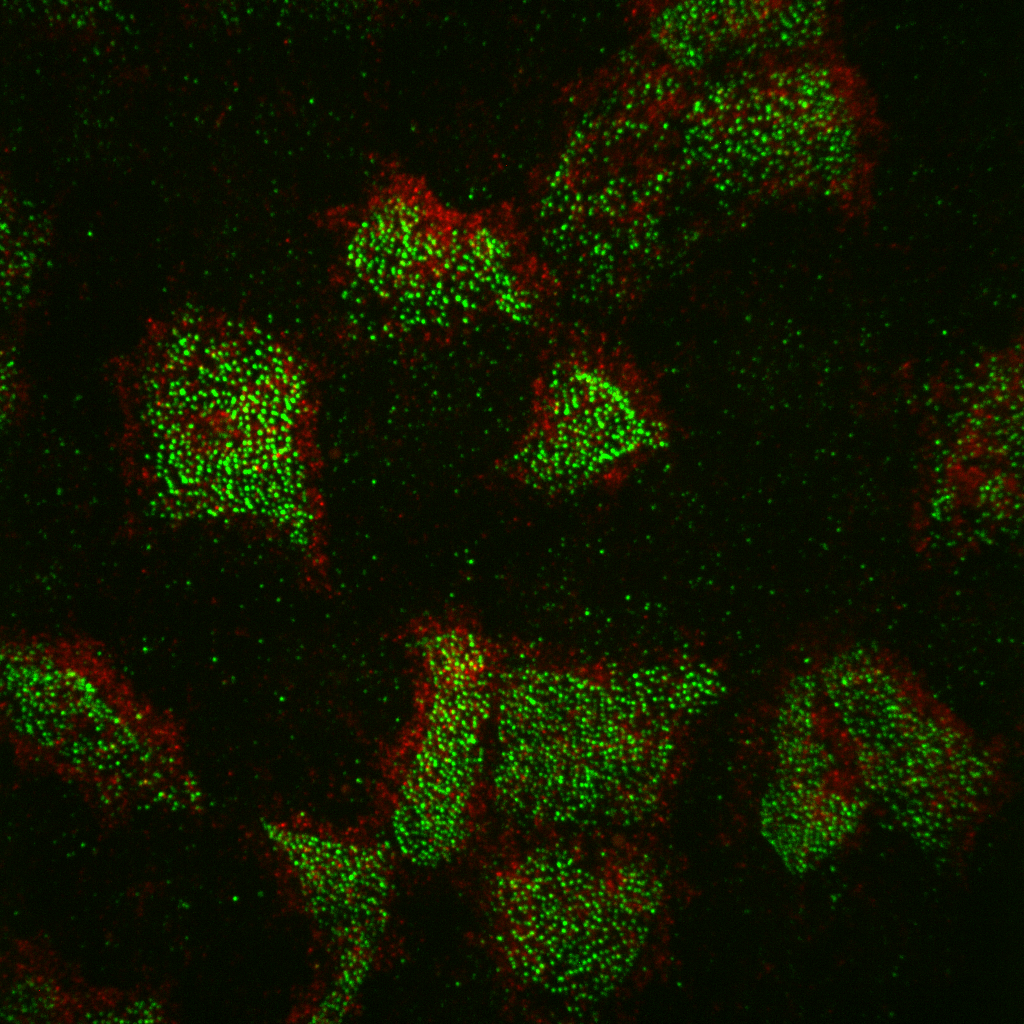

Supplement: Supplementary file 20 — Source Data Fig. 8 [file 44319_2024_66_MOESM20_ESM.zip › Source_Data_Figure_8/8B_trachea_tubulin/8B_KRT8-KO_trachea_Noc_tub_Large.tif]

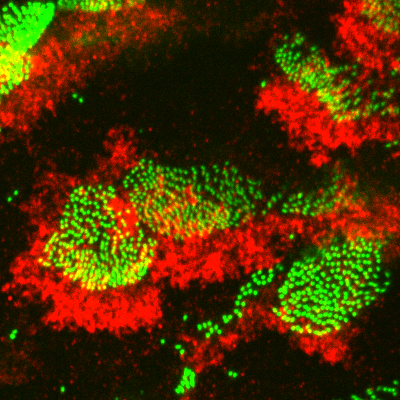

Supplement: Supplementary file 20 — Source Data Fig. 8 [file 44319_2024_66_MOESM20_ESM.zip › Source_Data_Figure_8/8B_trachea_tubulin/8B_WT_trachea_DMSO_tub.tif]

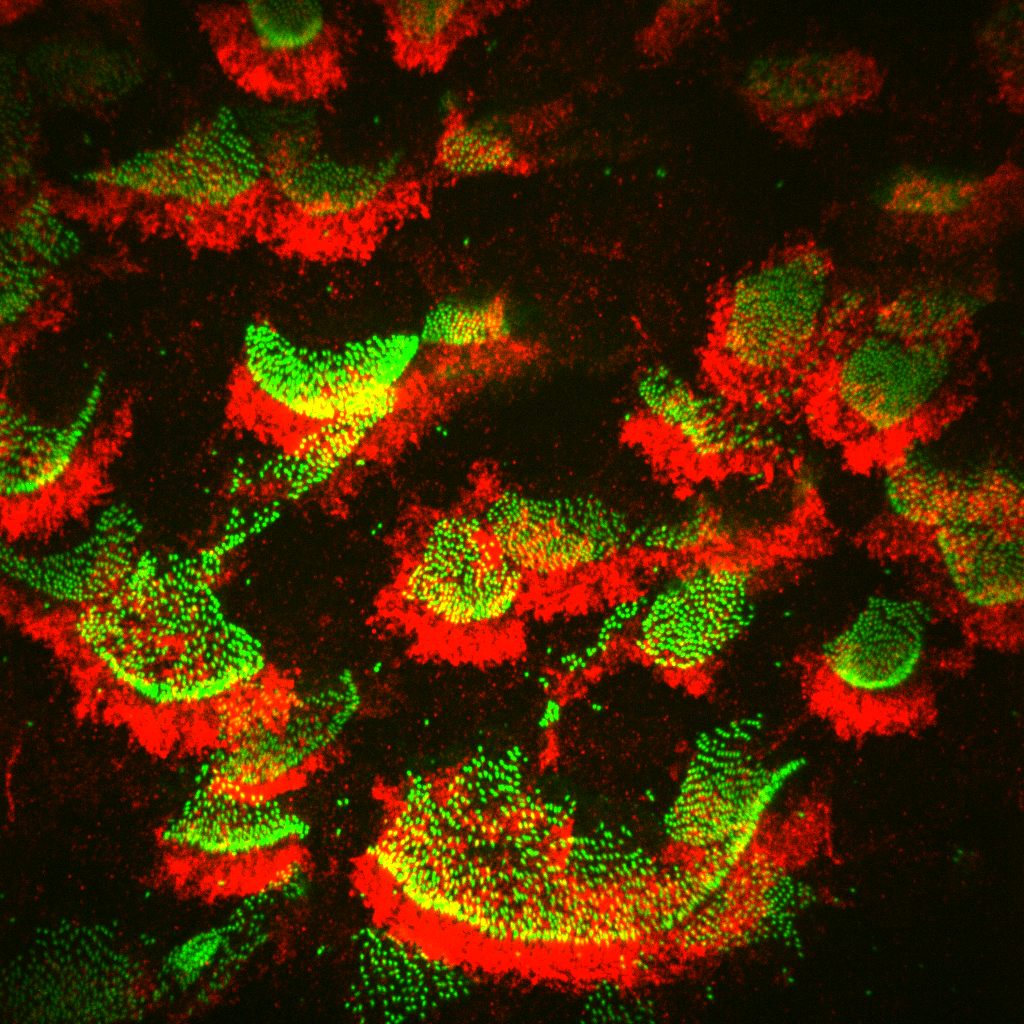

Supplement: Supplementary file 20 — Source Data Fig. 8 [file 44319_2024_66_MOESM20_ESM.zip › Source_Data_Figure_8/8B_trachea_tubulin/8B_WT_trachea_DMSO_tub_Large.tif]

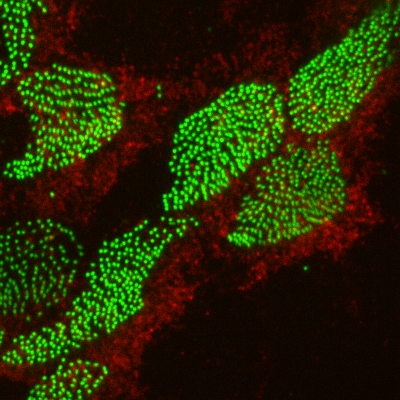

Supplement: Supplementary file 20 — Source Data Fig. 8 [file 44319_2024_66_MOESM20_ESM.zip › Source_Data_Figure_8/8B_trachea_tubulin/8B_WT_trachea_Noc_tub.tif]

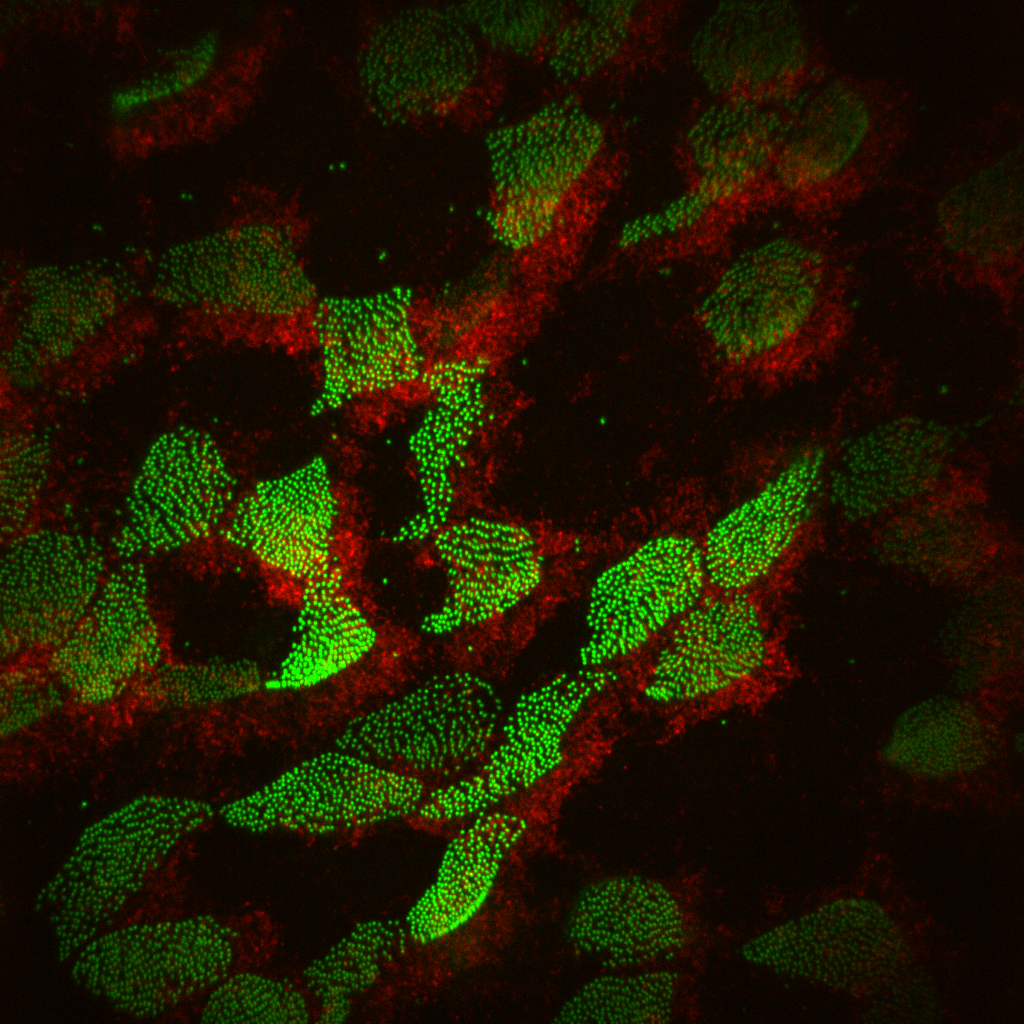

Supplement: Supplementary file 20 — Source Data Fig. 8 [file 44319_2024_66_MOESM20_ESM.zip › Source_Data_Figure_8/8B_trachea_tubulin/8B_WT_trachea_Noc_tub_Large.tif]

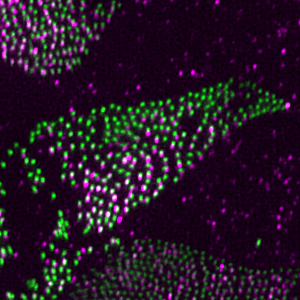

Supplement: Supplementary file 20 — Source Data Fig. 8 [file 44319_2024_66_MOESM20_ESM.zip › Source_Data_Figure_8/8C_KRT8-KO_trachea_DMSO/8C_KRT8-KO_trachea_DMSO_Stage1-3.tif]

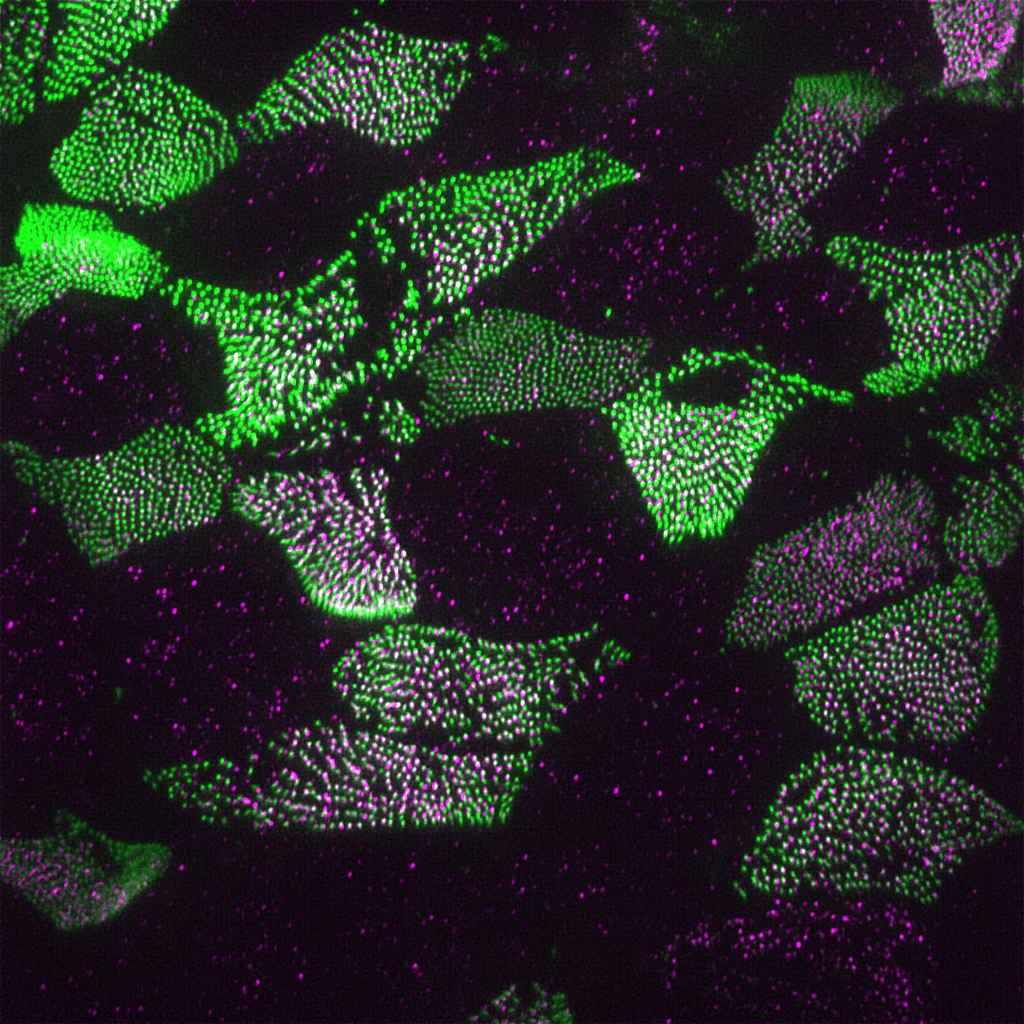

Supplement: Supplementary file 20 — Source Data Fig. 8 [file 44319_2024_66_MOESM20_ESM.zip › Source_Data_Figure_8/8C_KRT8-KO_trachea_DMSO/8C_KRT8-KO_trachea_DMSO_Stage1-3_Large.tif]

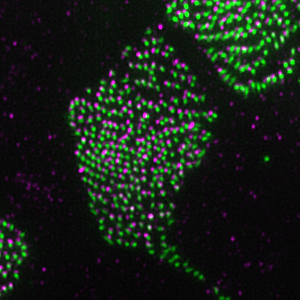

Supplement: Supplementary file 20 — Source Data Fig. 8 [file 44319_2024_66_MOESM20_ESM.zip › Source_Data_Figure_8/8C_KRT8-KO_trachea_DMSO/8C_KRT8-KO_trachea_DMSO_Stage4.tif]

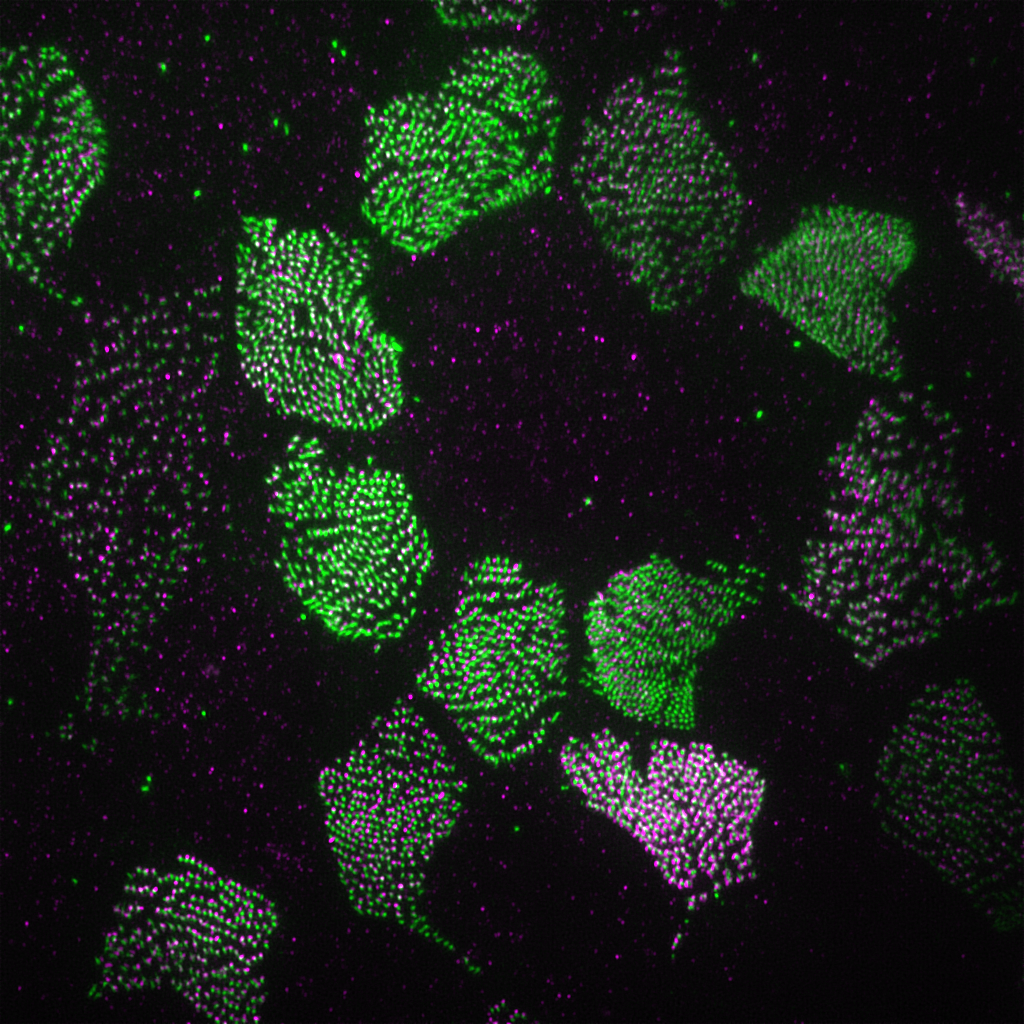

Supplement: Supplementary file 20 — Source Data Fig. 8 [file 44319_2024_66_MOESM20_ESM.zip › Source_Data_Figure_8/8C_KRT8-KO_trachea_DMSO/8C_KRT8-KO_trachea_DMSO_Stage4_Large.tif]

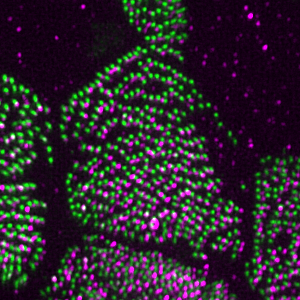

Supplement: Supplementary file 20 — Source Data Fig. 8 [file 44319_2024_66_MOESM20_ESM.zip › Source_Data_Figure_8/8C_KRT8-KO_trachea_DMSO/8C_KRT8-KO_trachea_DMSO_Stage5.tif]

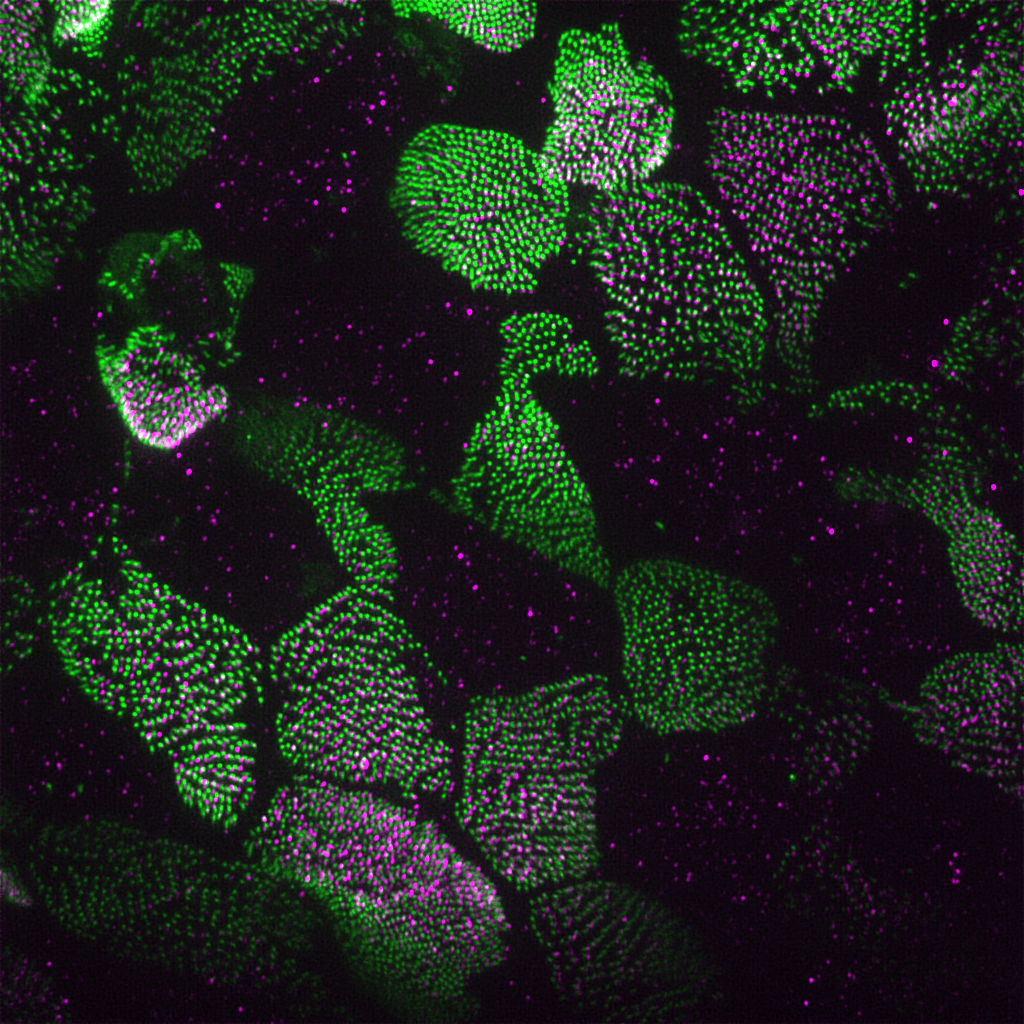

Supplement: Supplementary file 20 — Source Data Fig. 8 [file 44319_2024_66_MOESM20_ESM.zip › Source_Data_Figure_8/8C_KRT8-KO_trachea_DMSO/8C_KRT8-KO_trachea_DMSO_Stage5_Large.tif]

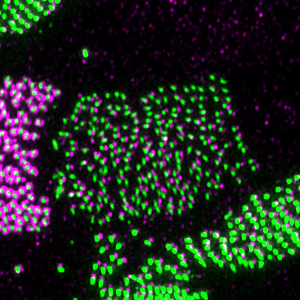

Supplement: Supplementary file 20 — Source Data Fig. 8 [file 44319_2024_66_MOESM20_ESM.zip › Source_Data_Figure_8/8C_WT_trachea_DMSO/8C_WT_trachea_DMSO_Stage4.tif]

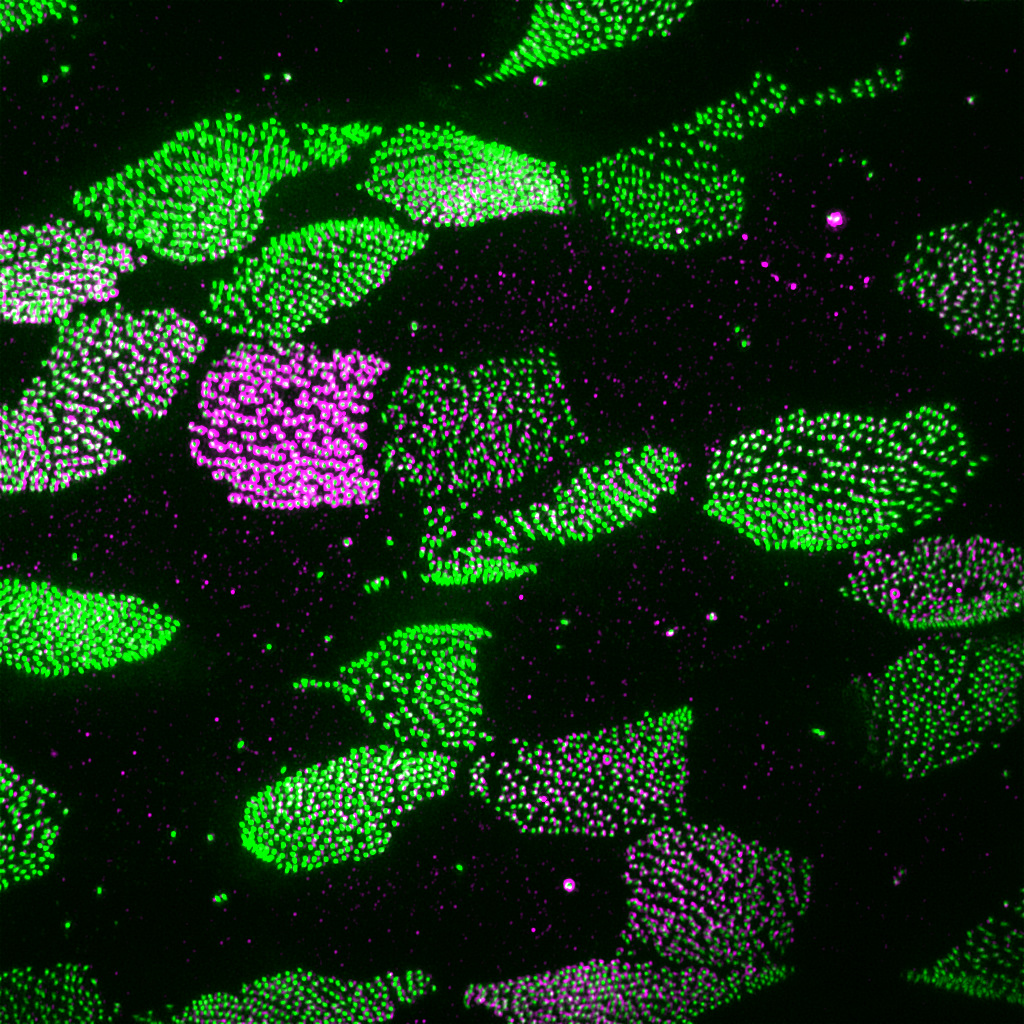

Supplement: Supplementary file 20 — Source Data Fig. 8 [file 44319_2024_66_MOESM20_ESM.zip › Source_Data_Figure_8/8C_WT_trachea_DMSO/8C_WT_trachea_DMSO_Stage4_Large.tif]

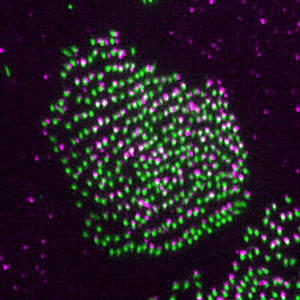

Supplement: Supplementary file 20 — Source Data Fig. 8 [file 44319_2024_66_MOESM20_ESM.zip › Source_Data_Figure_8/8C_WT_trachea_DMSO/8C_WT_trachea_DMSO_Stage5.tif]

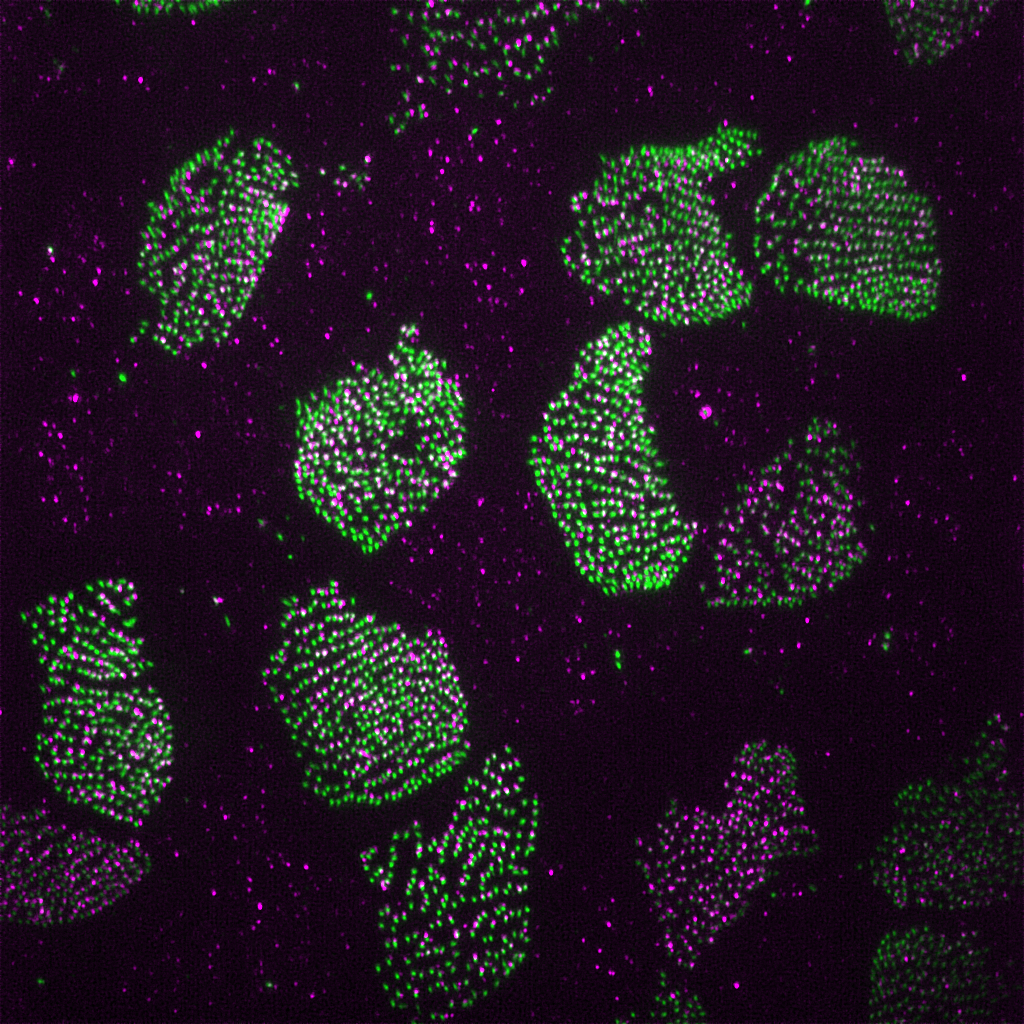

Supplement: Supplementary file 20 — Source Data Fig. 8 [file 44319_2024_66_MOESM20_ESM.zip › Source_Data_Figure_8/8C_WT_trachea_DMSO/8C_WT_trachea_DMSO_Stage5_Large.tif]

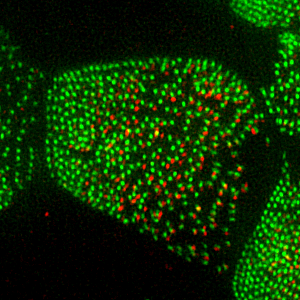

Supplement: Supplementary file 20 — Source Data Fig. 8 [file 44319_2024_66_MOESM20_ESM.zip › Source_Data_Figure_8/8C-F(EV5B-D)_trachea_Analysis/KRT8-KO_trachea_DMSO_Analysis(Cell_1-30).tif]

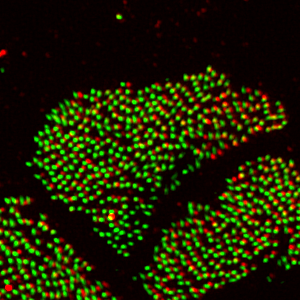

Supplement: Supplementary file 20 — Source Data Fig. 8 [file 44319_2024_66_MOESM20_ESM.zip › Source_Data_Figure_8/8C-F(EV5B-D)_trachea_Analysis/KRT8-KO_trachea_Noc_Analysis(Cell_1-28).tif]

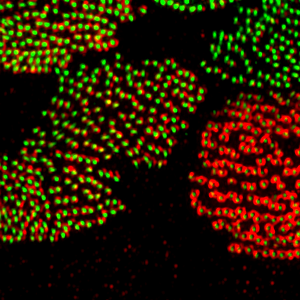

Supplement: Supplementary file 20 — Source Data Fig. 8 [file 44319_2024_66_MOESM20_ESM.zip › Source_Data_Figure_8/8C-F(EV5B-D)_trachea_Analysis/WT_trachea_DMSO_Analysis(Cell_1-28).tif]

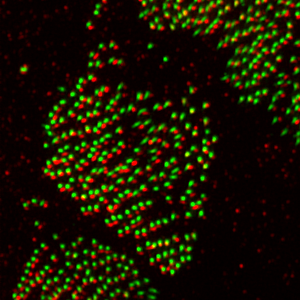

Supplement: Supplementary file 20 — Source Data Fig. 8 [file 44319_2024_66_MOESM20_ESM.zip › Source_Data_Figure_8/8C-F(EV5B-D)_trachea_Analysis/WT_trachea_Noc_Analysis(Cell_1-30).tif]

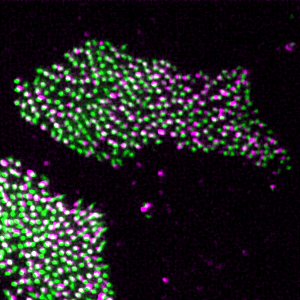

Supplement: Supplementary file 20 — Source Data Fig. 8 [file 44319_2024_66_MOESM20_ESM.zip › Source_Data_Figure_8/8D_KRT8-KO_trachea_Noc/8D_KRT8-KO_trachea_Noc_Stage1-3.tif]

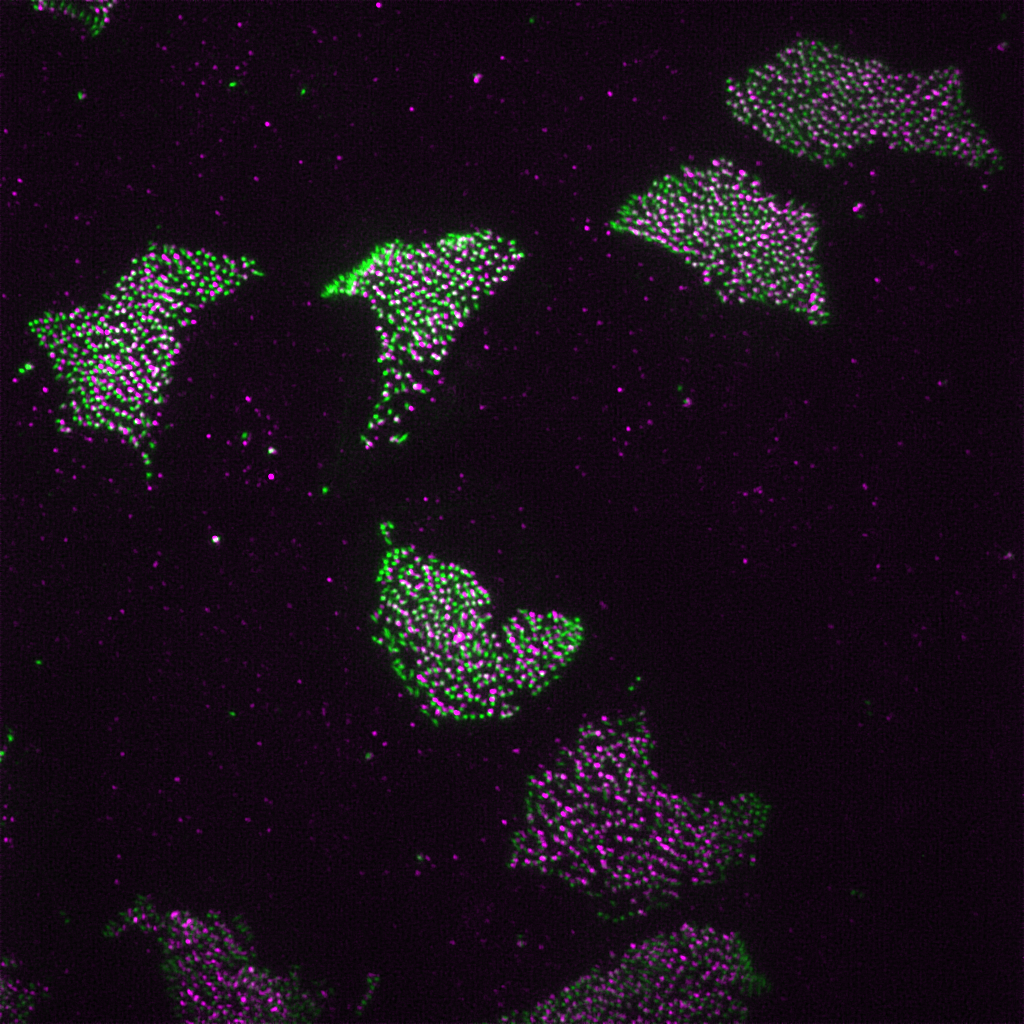

Supplement: Supplementary file 20 — Source Data Fig. 8 [file 44319_2024_66_MOESM20_ESM.zip › Source_Data_Figure_8/8D_KRT8-KO_trachea_Noc/8D_KRT8-KO_trachea_Noc_Stage1-3_Large.tif]

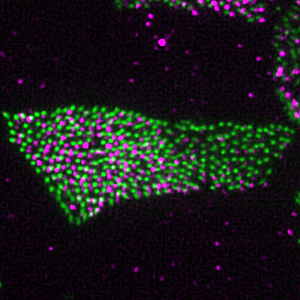

Supplement: Supplementary file 20 — Source Data Fig. 8 [file 44319_2024_66_MOESM20_ESM.zip › Source_Data_Figure_8/8D_KRT8-KO_trachea_Noc/8D_KRT8-KO_trachea_Noc_Stage4.tif]

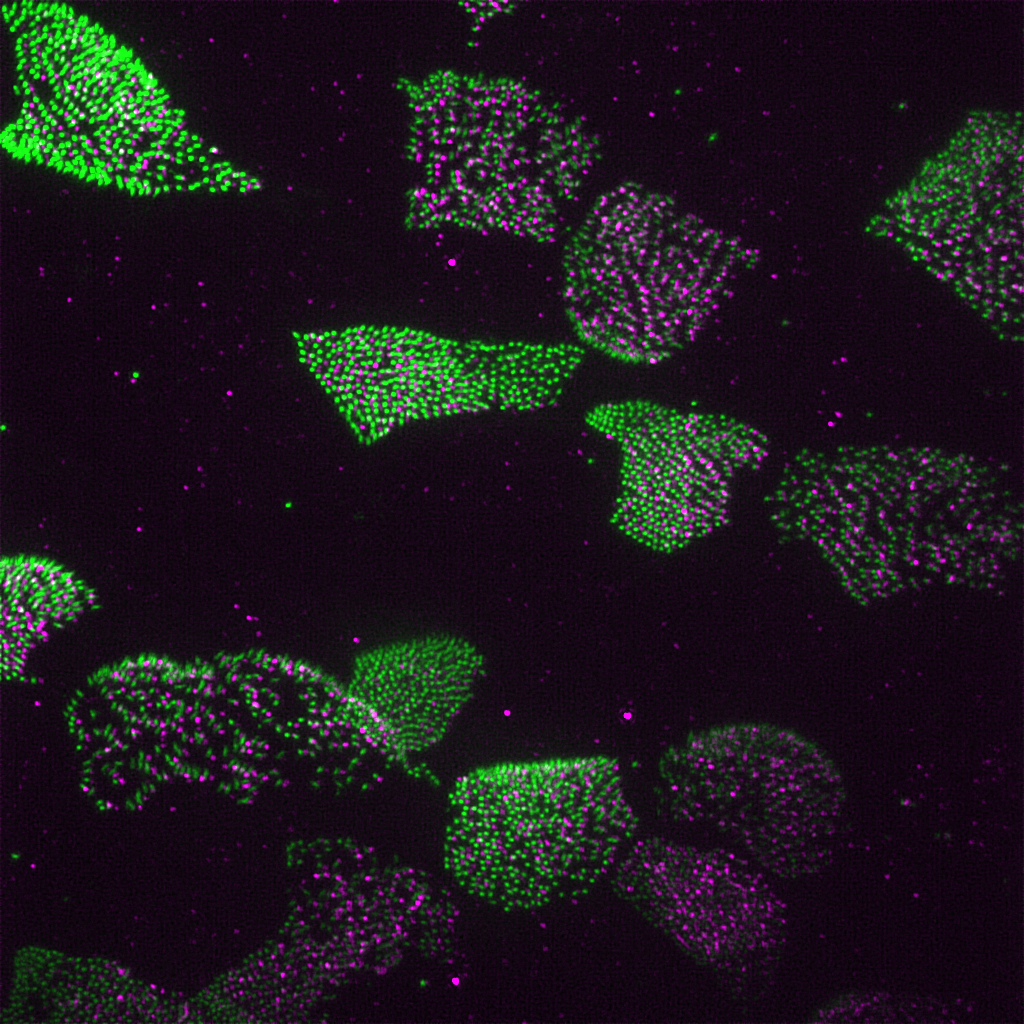

Supplement: Supplementary file 20 — Source Data Fig. 8 [file 44319_2024_66_MOESM20_ESM.zip › Source_Data_Figure_8/8D_KRT8-KO_trachea_Noc/8D_KRT8-KO_trachea_Noc_Stage4_Large.tif]

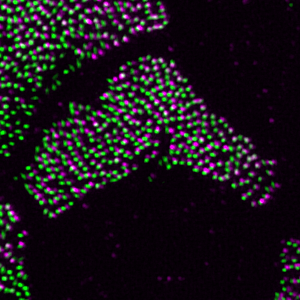

Supplement: Supplementary file 20 — Source Data Fig. 8 [file 44319_2024_66_MOESM20_ESM.zip › Source_Data_Figure_8/8D_KRT8-KO_trachea_Noc/8D_KRT8-KO_trachea_Noc_Stage5.tif]

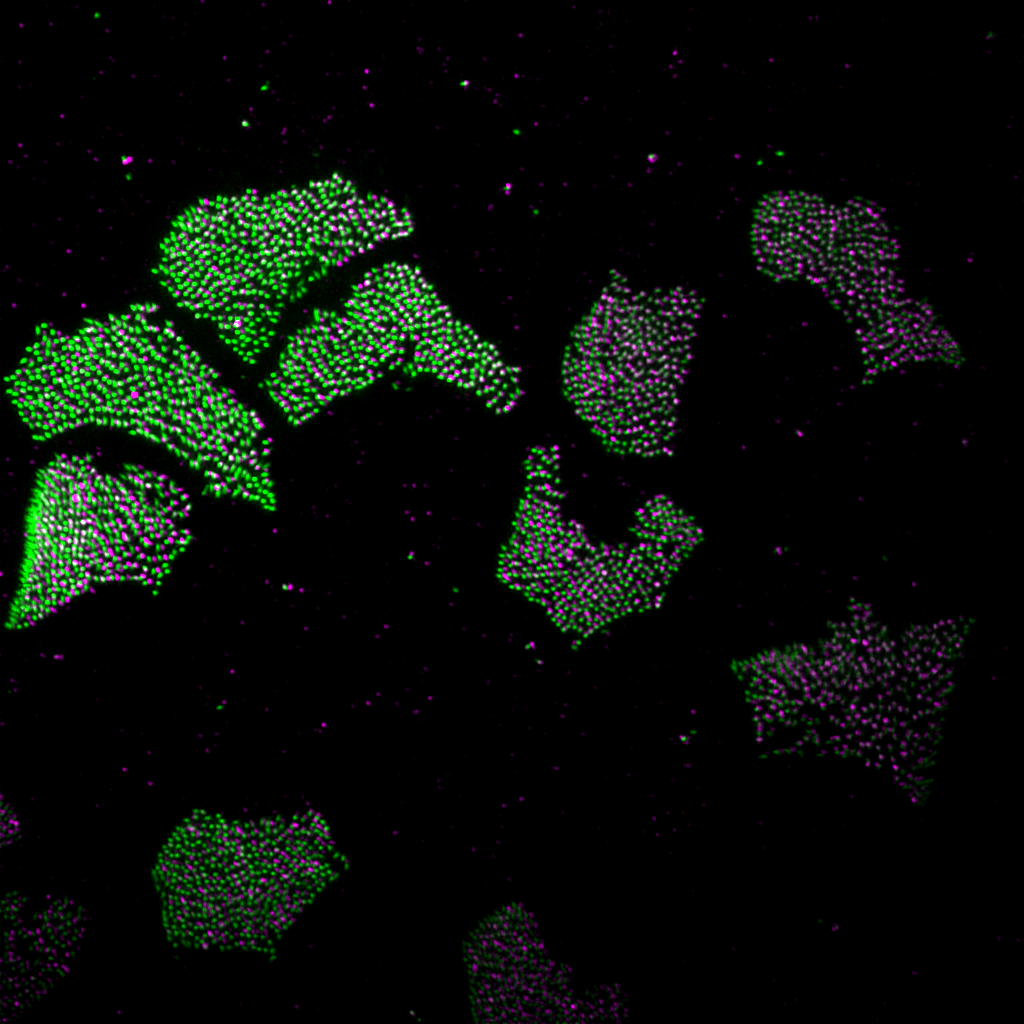

Supplement: Supplementary file 20 — Source Data Fig. 8 [file 44319_2024_66_MOESM20_ESM.zip › Source_Data_Figure_8/8D_KRT8-KO_trachea_Noc/8D_KRT8-KO_trachea_Noc_Stage5_Large.tif]

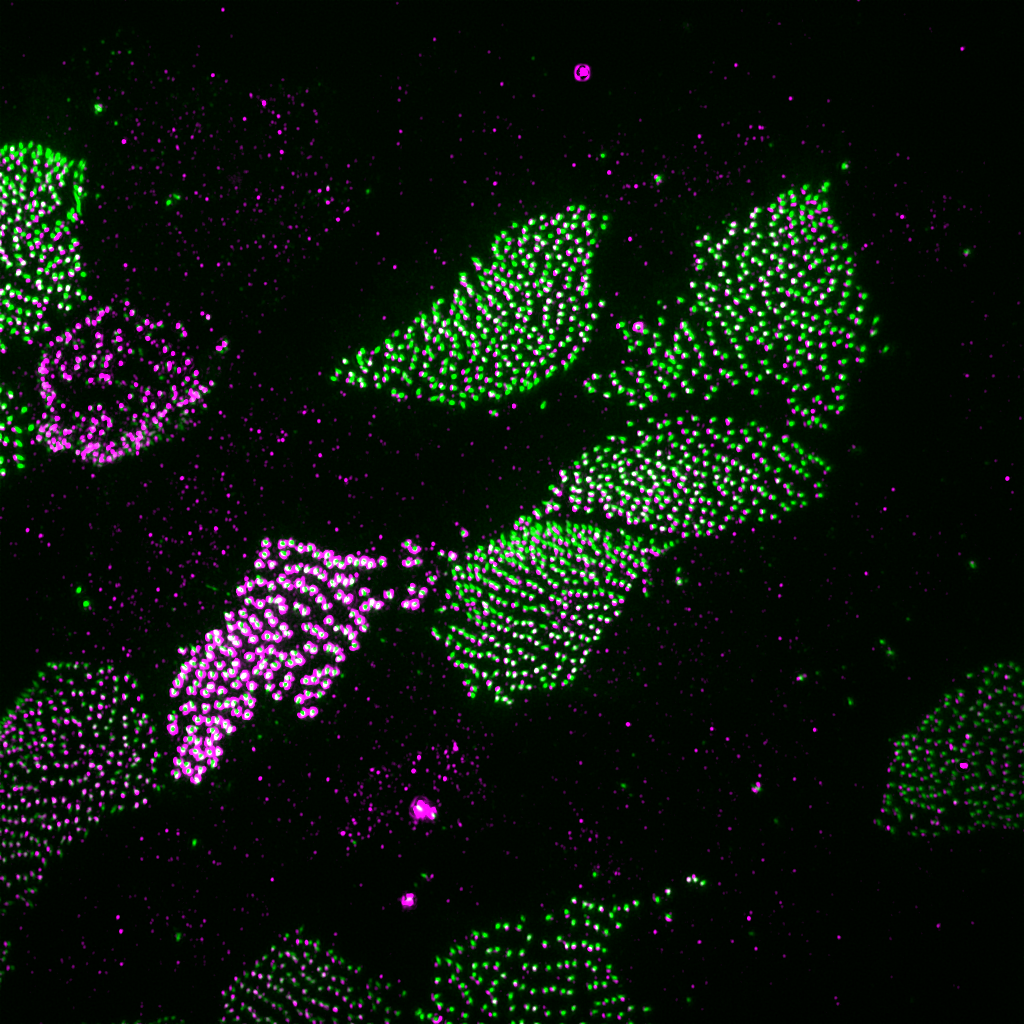

Supplement: Supplementary file 20 — Source Data Fig. 8 [file 44319_2024_66_MOESM20_ESM.zip › Source_Data_Figure_8/8D_WT_trachea_Noc/8D_WT_trachea_Noc_Stage4(Stage5)_Large.tif]

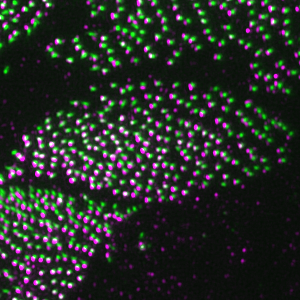

Supplement: Supplementary file 20 — Source Data Fig. 8 [file 44319_2024_66_MOESM20_ESM.zip › Source_Data_Figure_8/8D_WT_trachea_Noc/8D_WT_trachea_Noc_Stage4.tif]

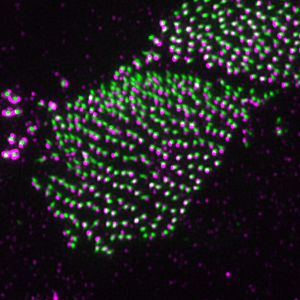

Supplement: Supplementary file 20 — Source Data Fig. 8 [file 44319_2024_66_MOESM20_ESM.zip › Source_Data_Figure_8/8D_WT_trachea_Noc/8D_WT_trachea_Noc_Stage5.tif]

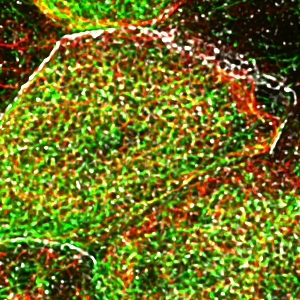

Supplement: Supplementary file 21 — Figure Source Data - EV and Appendix Figures [file 44319_2024_66_MOESM21_ESM.zip › Appendix_Source_Data/Appendix_Figure_S4/S4A_MTEC_Early_TP/S4A_Early_TP_DMSO.tif]

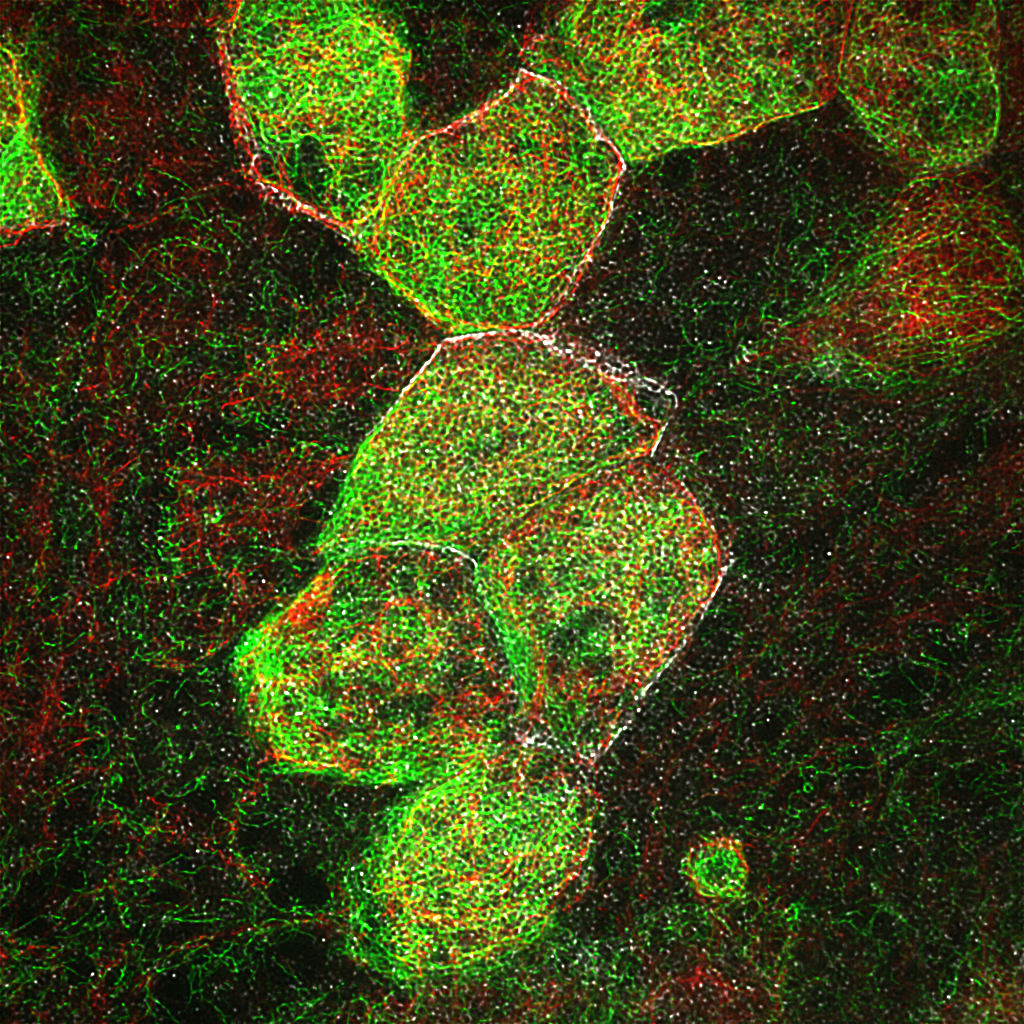

Supplement: Supplementary file 21 — Figure Source Data - EV and Appendix Figures [file 44319_2024_66_MOESM21_ESM.zip › Appendix_Source_Data/Appendix_Figure_S4/S4A_MTEC_Early_TP/S4A_Early_TP_DMSO_Large.tif]

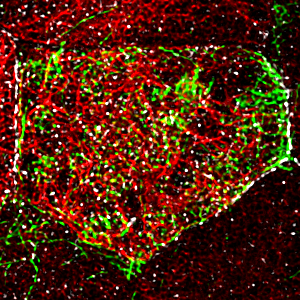

Supplement: Supplementary file 21 — Figure Source Data - EV and Appendix Figures [file 44319_2024_66_MOESM21_ESM.zip › Appendix_Source_Data/Appendix_Figure_S4/S4A_MTEC_Early_TP/S4A_Early_TP_Noc.tif]

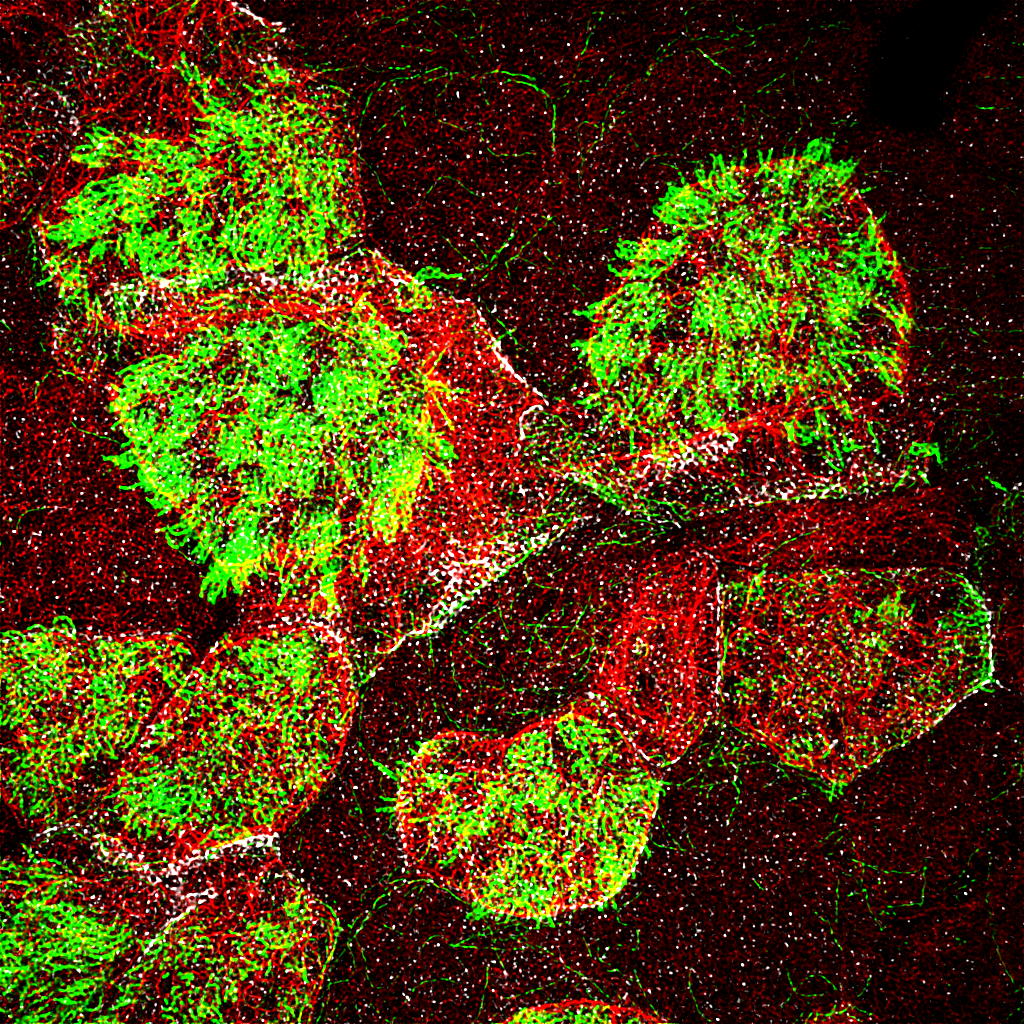

Supplement: Supplementary file 21 — Figure Source Data - EV and Appendix Figures [file 44319_2024_66_MOESM21_ESM.zip › Appendix_Source_Data/Appendix_Figure_S4/S4A_MTEC_Early_TP/S4A_Early_TP_Noc_Large.tif]

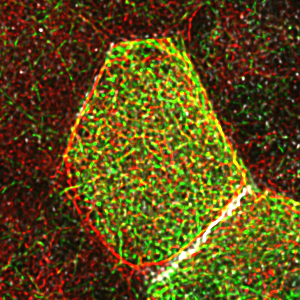

Supplement: Supplementary file 21 — Figure Source Data - EV and Appendix Figures [file 44319_2024_66_MOESM21_ESM.zip › Appendix_Source_Data/Appendix_Figure_S4/S4B_MTEC_Late_TP/S4B_Late_TP_DMSO.tif]

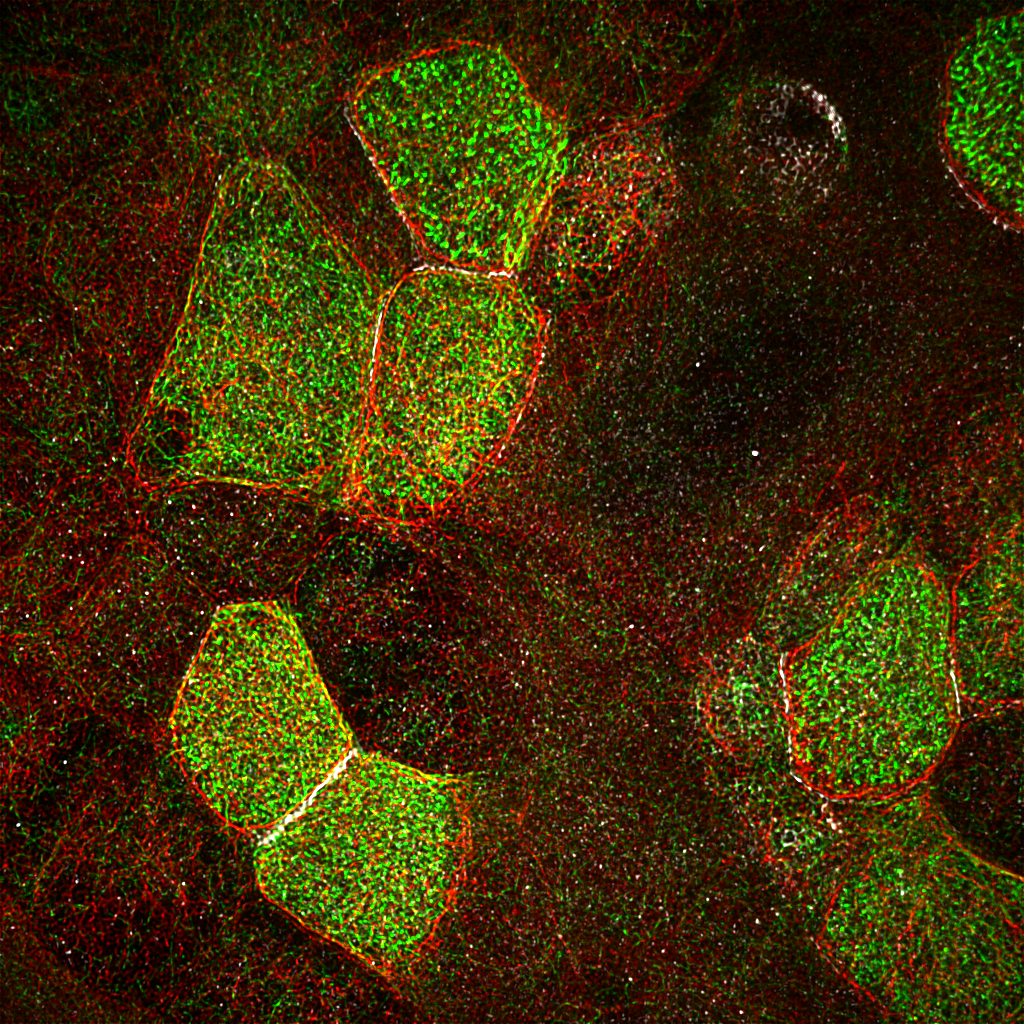

Supplement: Supplementary file 21 — Figure Source Data - EV and Appendix Figures [file 44319_2024_66_MOESM21_ESM.zip › Appendix_Source_Data/Appendix_Figure_S4/S4B_MTEC_Late_TP/S4B_Late_TP_DMSO_Large.tif]

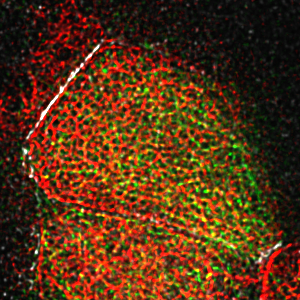

Supplement: Supplementary file 21 — Figure Source Data - EV and Appendix Figures [file 44319_2024_66_MOESM21_ESM.zip › Appendix_Source_Data/Appendix_Figure_S4/S4B_MTEC_Late_TP/S4B_Late_TP_Noc.tif]

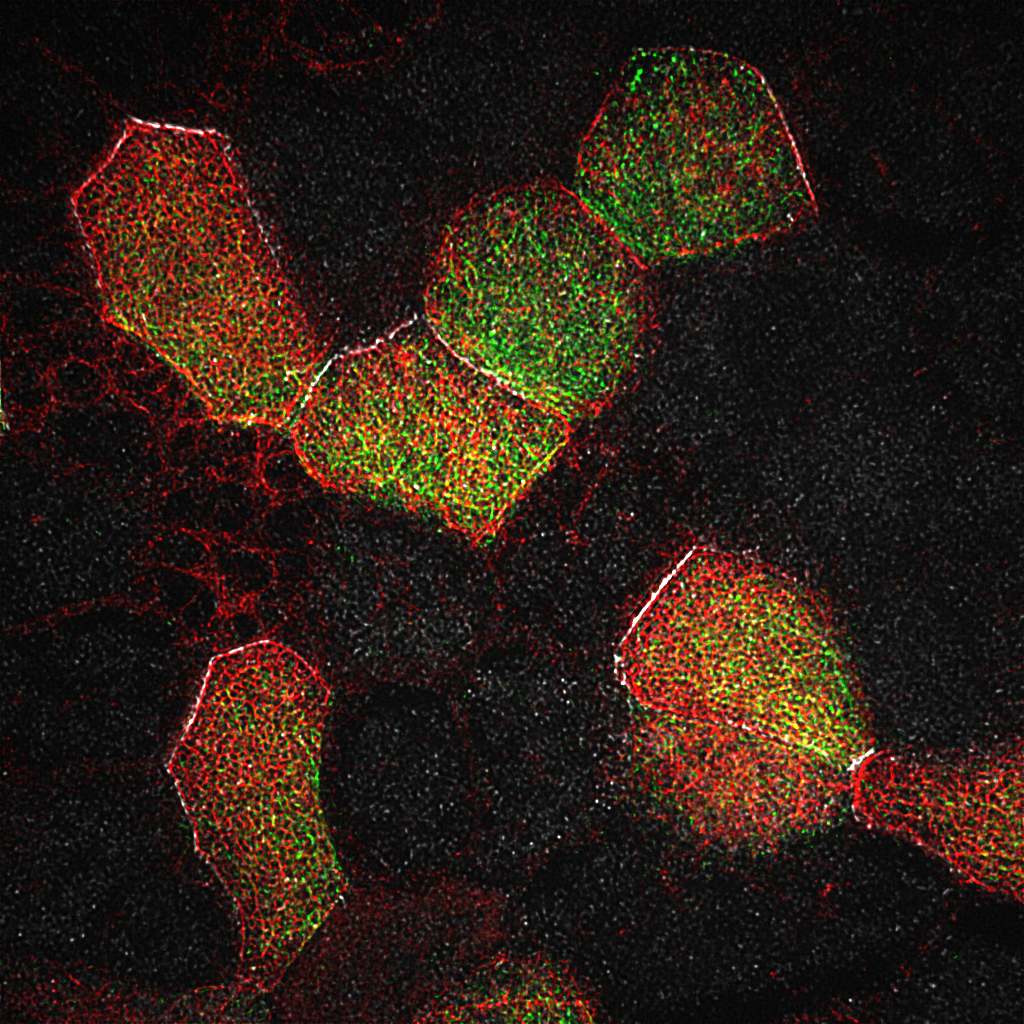

Supplement: Supplementary file 21 — Figure Source Data - EV and Appendix Figures [file 44319_2024_66_MOESM21_ESM.zip › Appendix_Source_Data/Appendix_Figure_S4/S4B_MTEC_Late_TP/S4B_Late_TP_Noc_Large.tif]

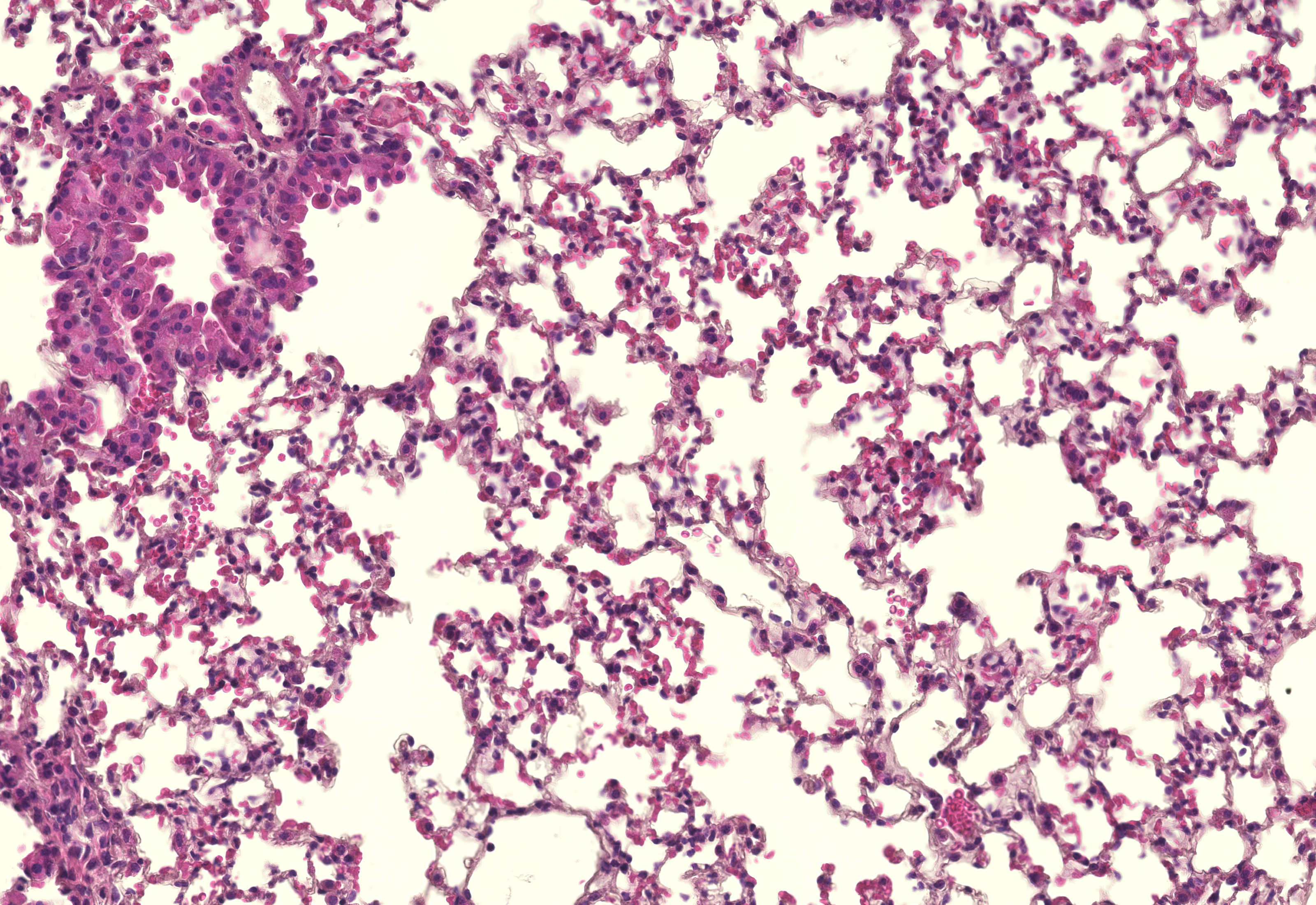

Supplement: Supplementary file 21 — Figure Source Data - EV and Appendix Figures [file 44319_2024_66_MOESM21_ESM.zip › Appendix_Source_Data/Appendix_Figure_S5/S5A_HE/S5A_KRT8KO_HE.tif]

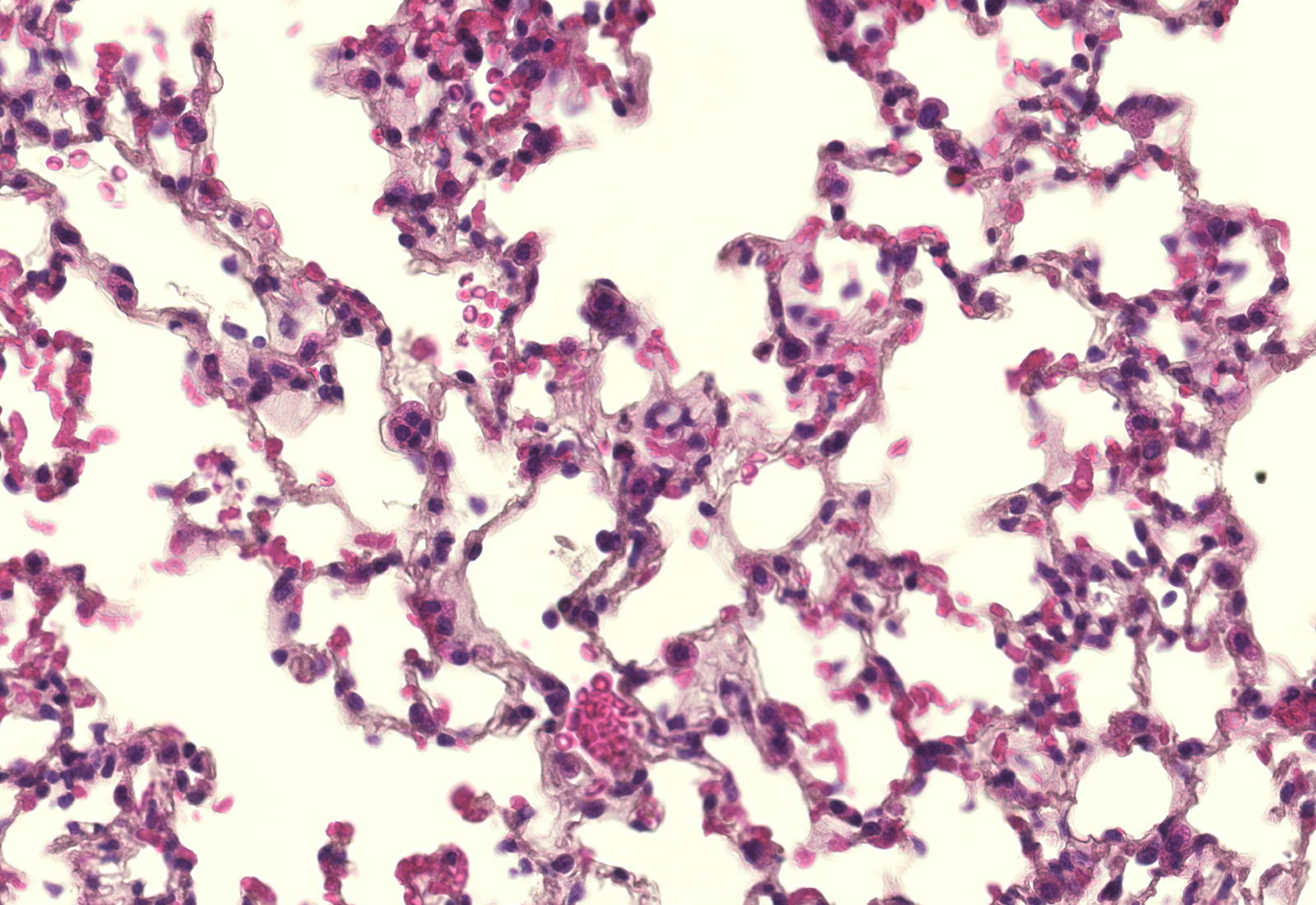

Supplement: Supplementary file 21 — Figure Source Data - EV and Appendix Figures [file 44319_2024_66_MOESM21_ESM.zip › Appendix_Source_Data/Appendix_Figure_S5/S5A_HE/S5A_KRT8KO_HE_Alveoli.tif]

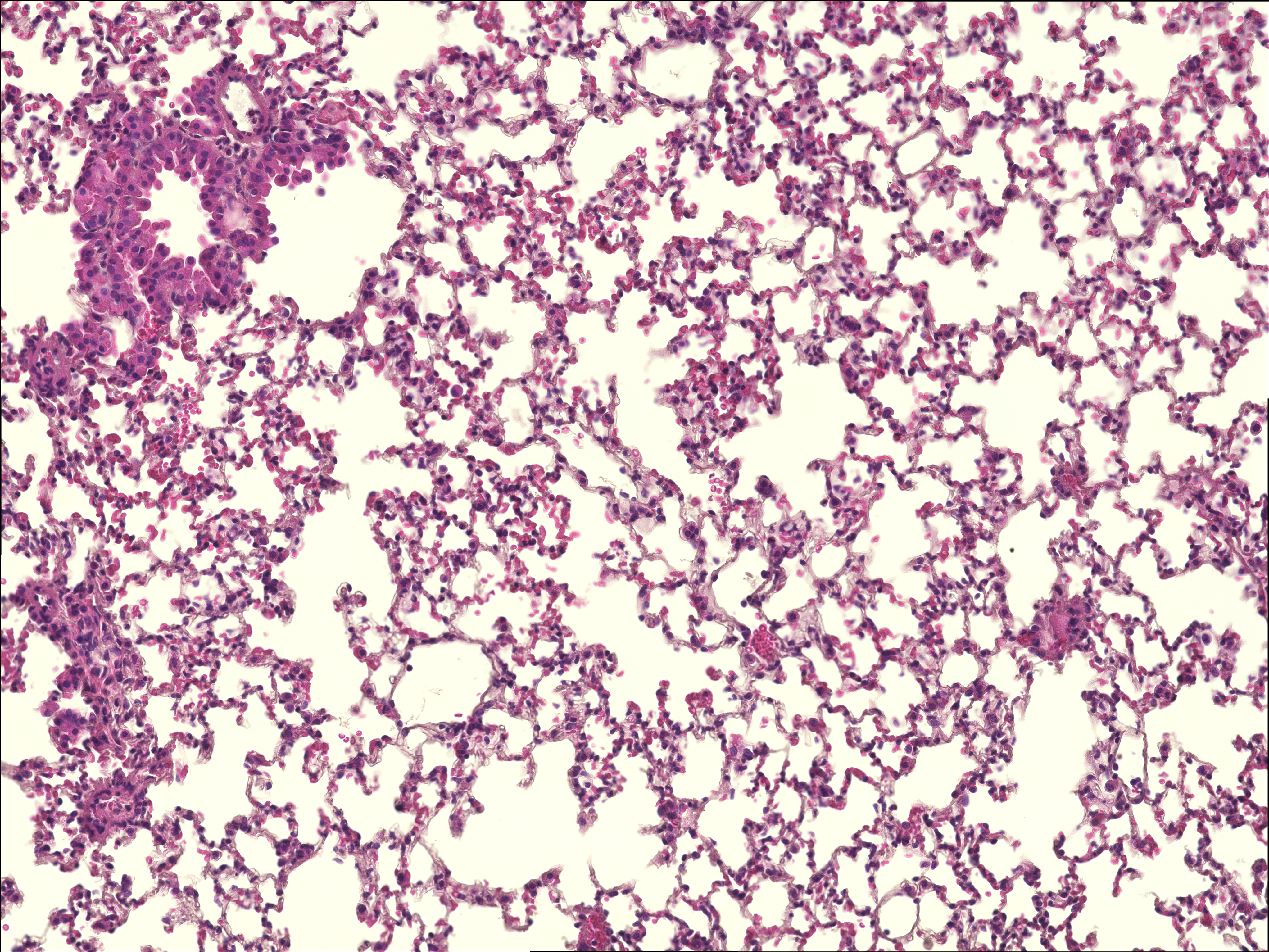

Supplement: Supplementary file 21 — Figure Source Data - EV and Appendix Figures [file 44319_2024_66_MOESM21_ESM.zip › Appendix_Source_Data/Appendix_Figure_S5/S5A_HE/S5A_KRT8KO_HE_Large.tif]

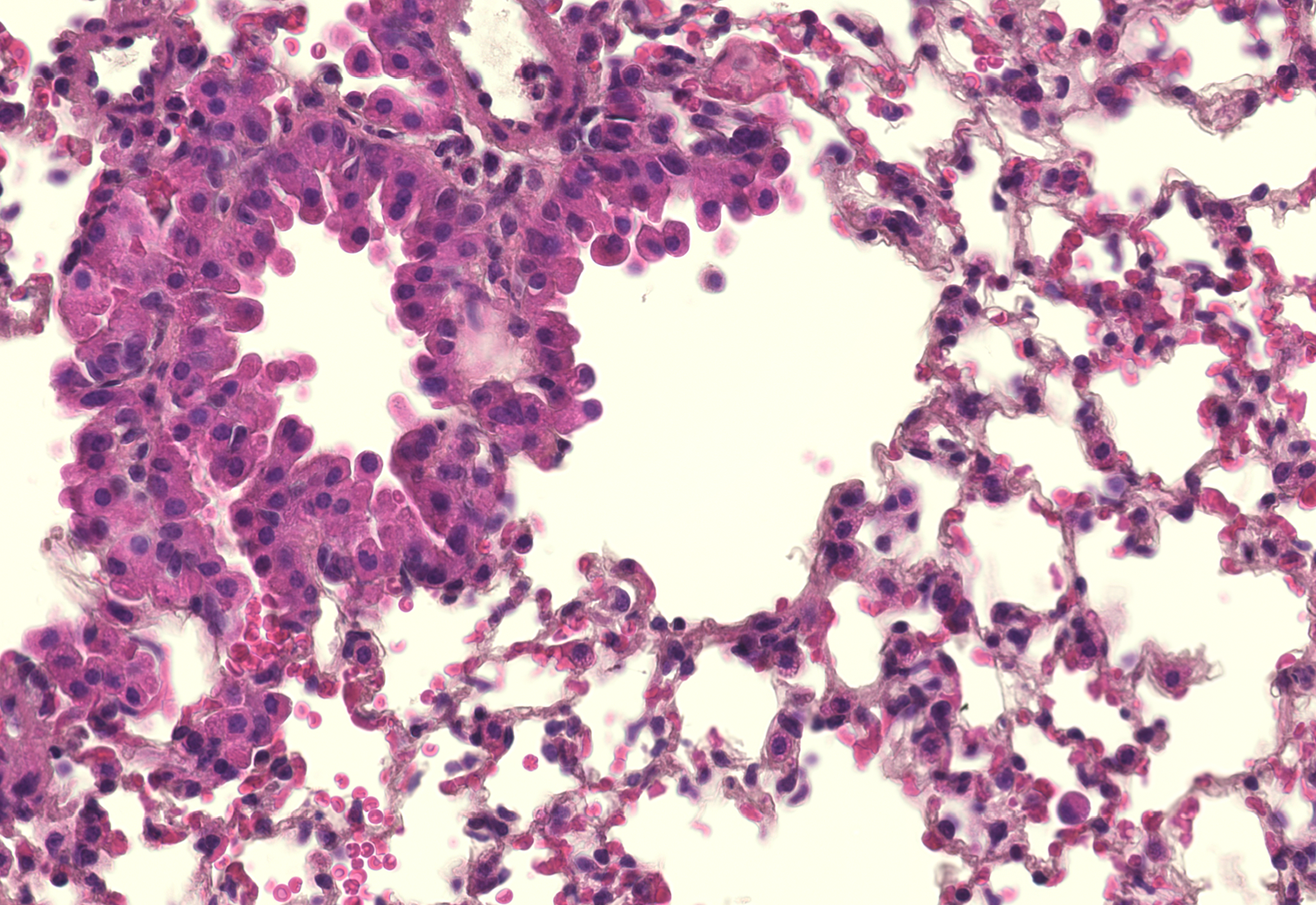

Supplement: Supplementary file 21 — Figure Source Data - EV and Appendix Figures [file 44319_2024_66_MOESM21_ESM.zip › Appendix_Source_Data/Appendix_Figure_S5/S5A_HE/S5A_KRT8KO_HE_Tracgea.tif]

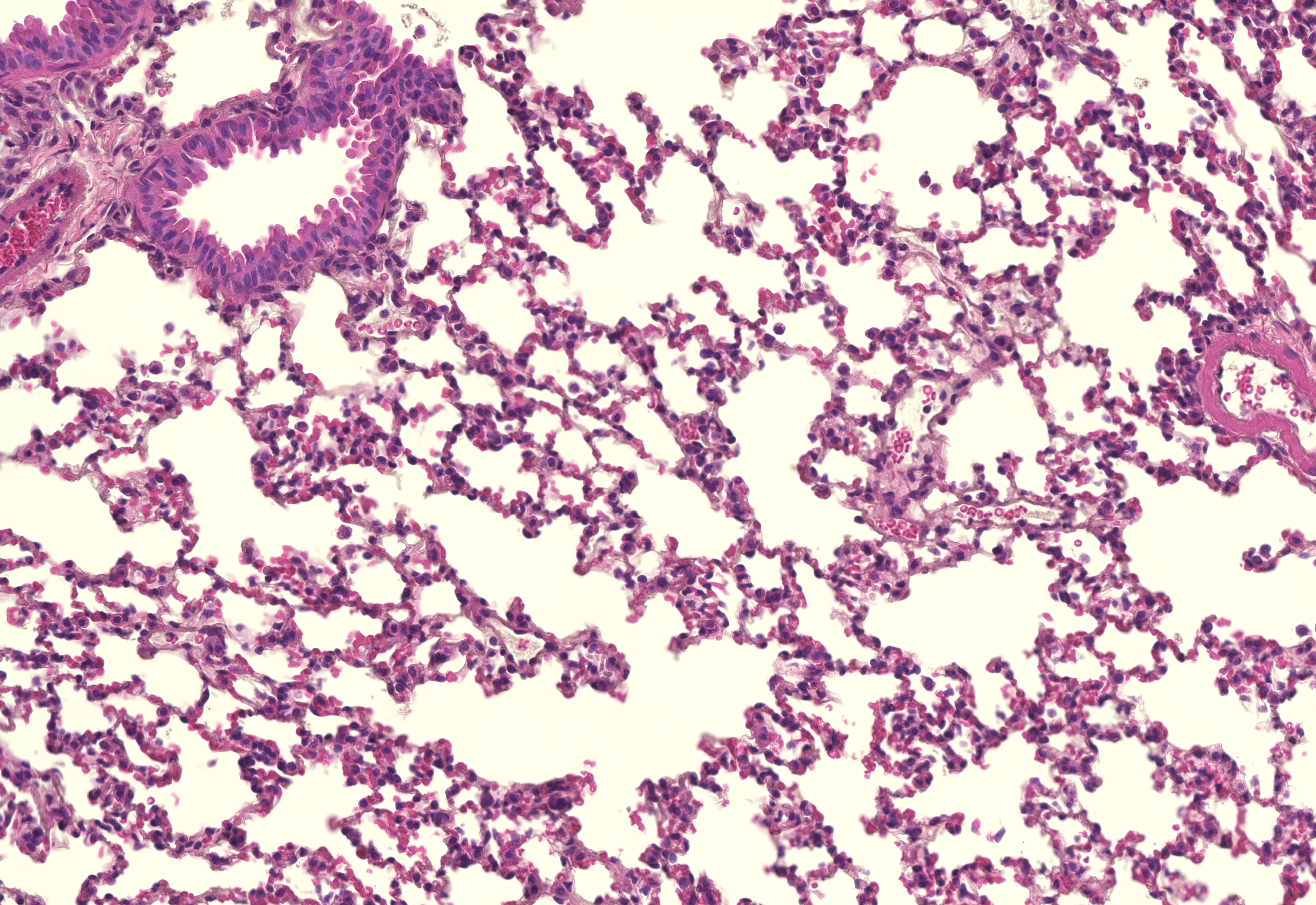

Supplement: Supplementary file 21 — Figure Source Data - EV and Appendix Figures [file 44319_2024_66_MOESM21_ESM.zip › Appendix_Source_Data/Appendix_Figure_S5/S5A_HE/S5A_WT_HE.tif]

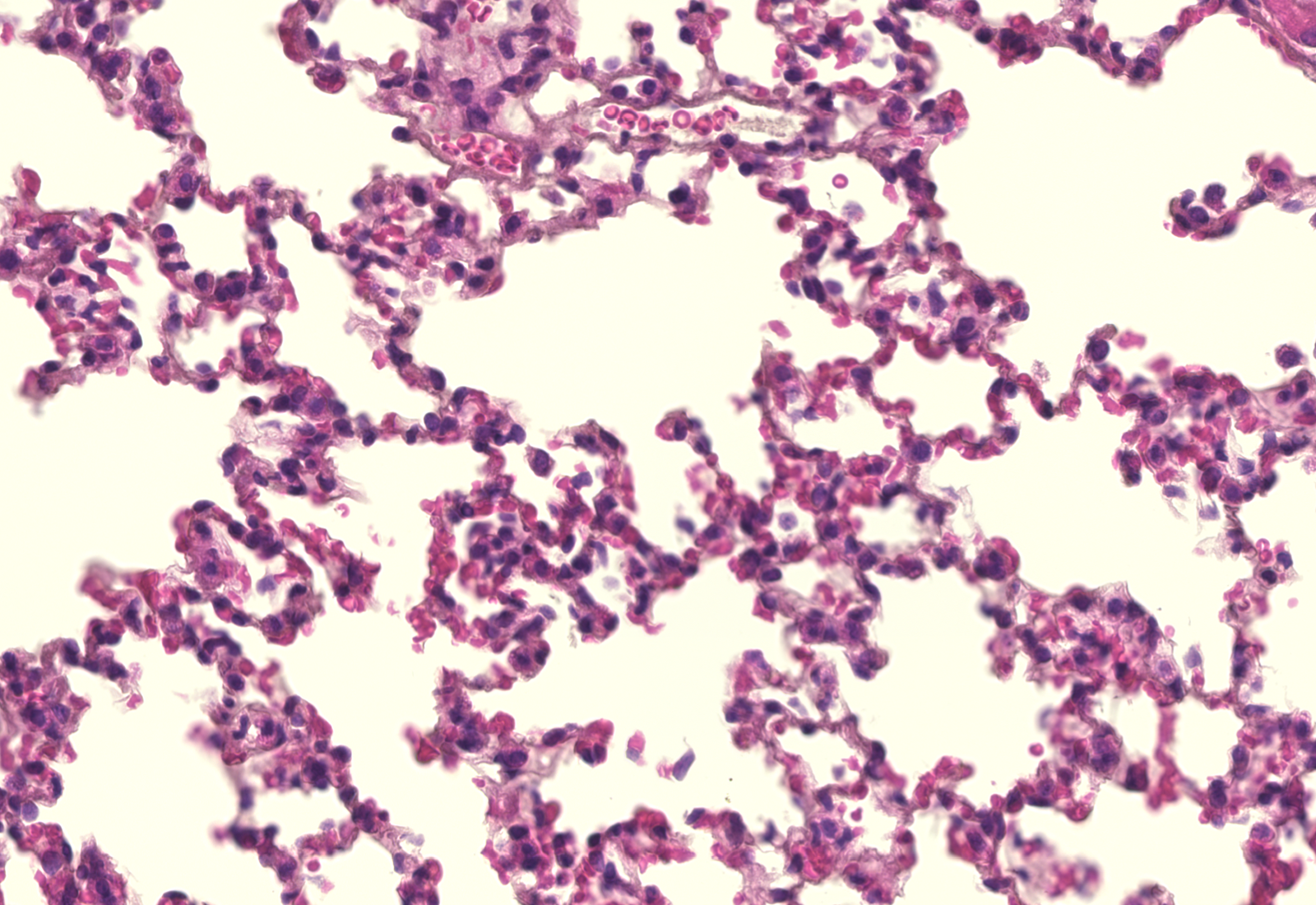

Supplement: Supplementary file 21 — Figure Source Data - EV and Appendix Figures [file 44319_2024_66_MOESM21_ESM.zip › Appendix_Source_Data/Appendix_Figure_S5/S5A_HE/S5A_WT_HE_Alveoli.tif]

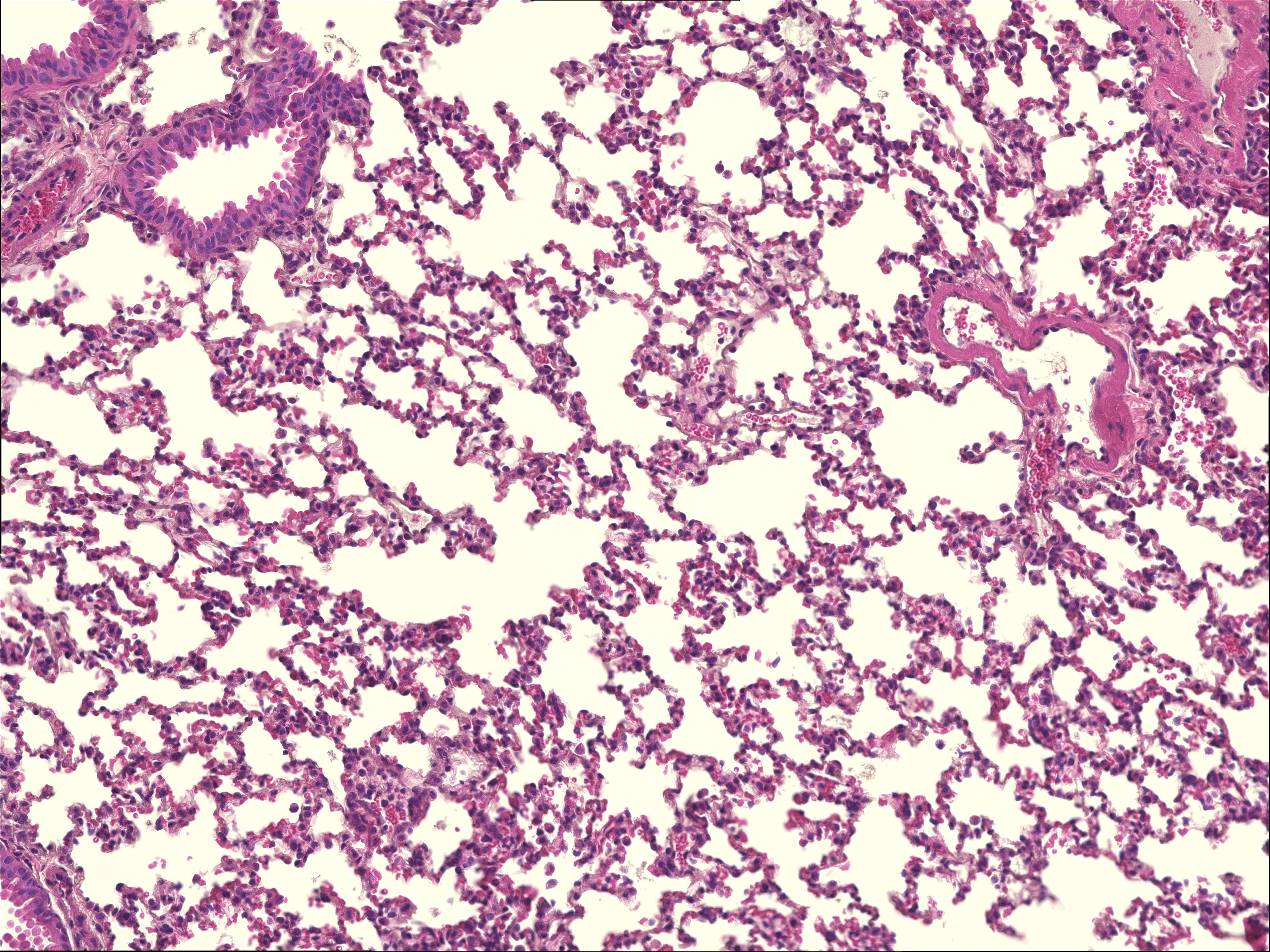

Supplement: Supplementary file 21 — Figure Source Data - EV and Appendix Figures [file 44319_2024_66_MOESM21_ESM.zip › Appendix_Source_Data/Appendix_Figure_S5/S5A_HE/S5A_WT_HE_Large.tif]

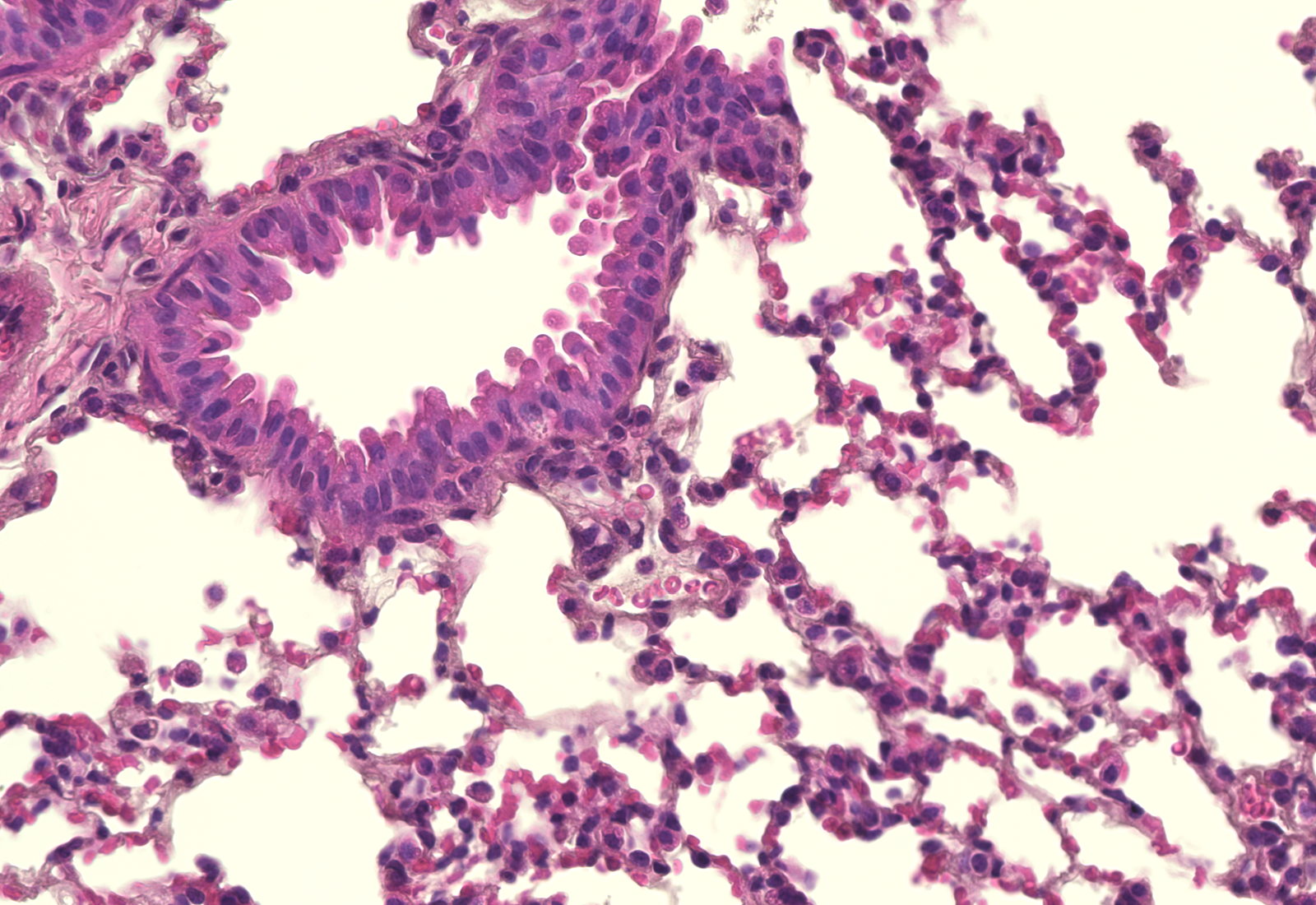

Supplement: Supplementary file 21 — Figure Source Data - EV and Appendix Figures [file 44319_2024_66_MOESM21_ESM.zip › Appendix_Source_Data/Appendix_Figure_S5/S5A_HE/S5A_WT_HE_Trachea.tif]

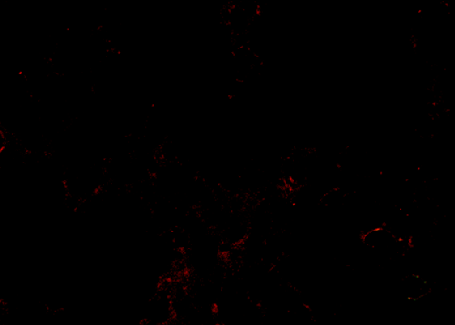

Supplement: Supplementary file 21 — Figure Source Data - EV and Appendix Figures [file 44319_2024_66_MOESM21_ESM.zip › Appendix_Source_Data/Appendix_Figure_S5/S5B_keratin18/S5B_KRT8KO_K18_High_Mag.tif]

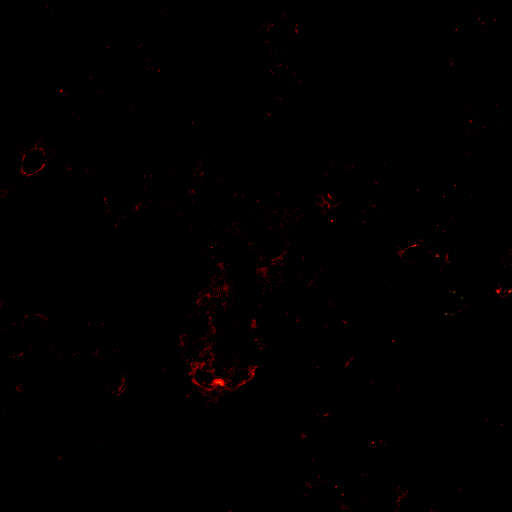

Supplement: Supplementary file 21 — Figure Source Data - EV and Appendix Figures [file 44319_2024_66_MOESM21_ESM.zip › Appendix_Source_Data/Appendix_Figure_S5/S5B_keratin18/S5B_KRT8KO_K18_High_Mag_Large.tif]

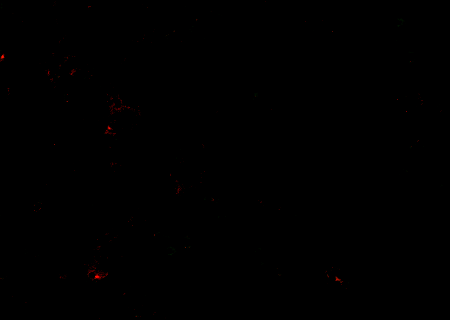

Supplement: Supplementary file 21 — Figure Source Data - EV and Appendix Figures [file 44319_2024_66_MOESM21_ESM.zip › Appendix_Source_Data/Appendix_Figure_S5/S5B_keratin18/S5B_KRT8KO_K18_Low_Mag.tif]

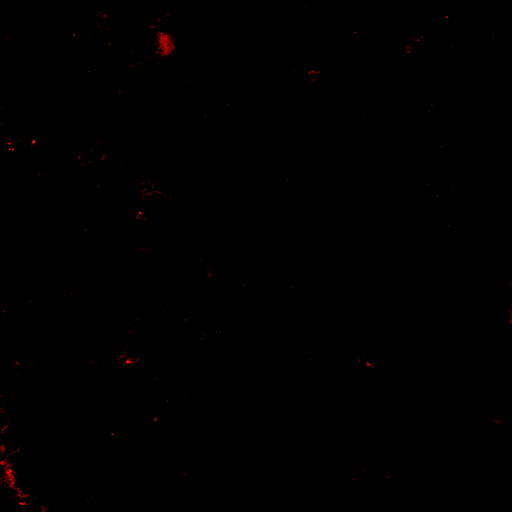

Supplement: Supplementary file 21 — Figure Source Data - EV and Appendix Figures [file 44319_2024_66_MOESM21_ESM.zip › Appendix_Source_Data/Appendix_Figure_S5/S5B_keratin18/S5B_KRT8KO_K18_Low_Mag_Large.tif]

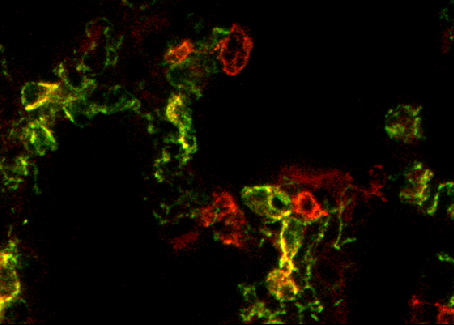

Supplement: Supplementary file 21 — Figure Source Data - EV and Appendix Figures [file 44319_2024_66_MOESM21_ESM.zip › Appendix_Source_Data/Appendix_Figure_S5/S5B_keratin18/S5B_WT_K18_High_Mag.tif]

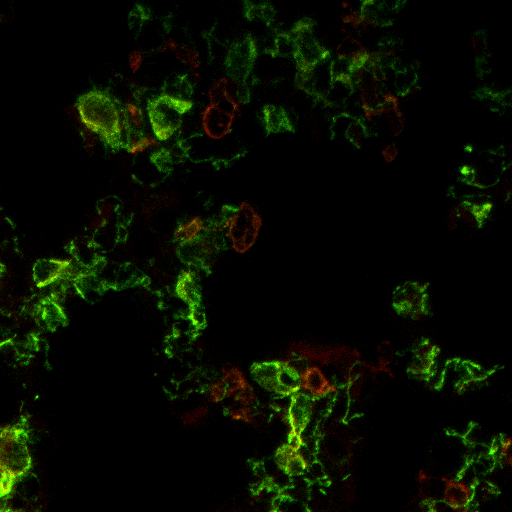

Supplement: Supplementary file 21 — Figure Source Data - EV and Appendix Figures [file 44319_2024_66_MOESM21_ESM.zip › Appendix_Source_Data/Appendix_Figure_S5/S5B_keratin18/S5B_WT_K18_High_Mag_Large.tif]

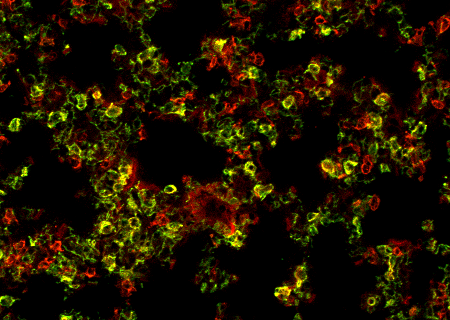

Supplement: Supplementary file 21 — Figure Source Data - EV and Appendix Figures [file 44319_2024_66_MOESM21_ESM.zip › Appendix_Source_Data/Appendix_Figure_S5/S5B_keratin18/S5B_WT_K18_Low_Mag.tif]

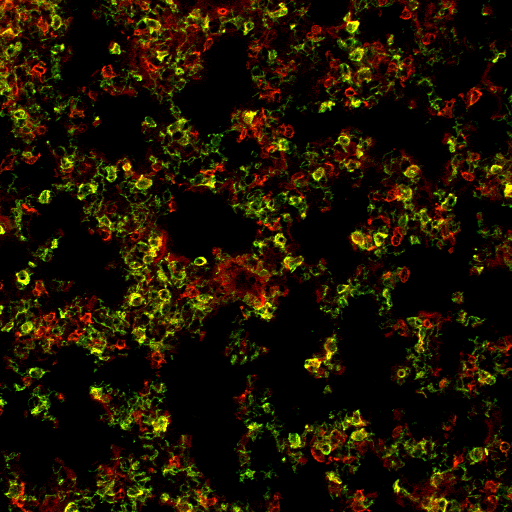

Supplement: Supplementary file 21 — Figure Source Data - EV and Appendix Figures [file 44319_2024_66_MOESM21_ESM.zip › Appendix_Source_Data/Appendix_Figure_S5/S5B_keratin18/S5B_WT_K18_Low_Mag_Large.tif]

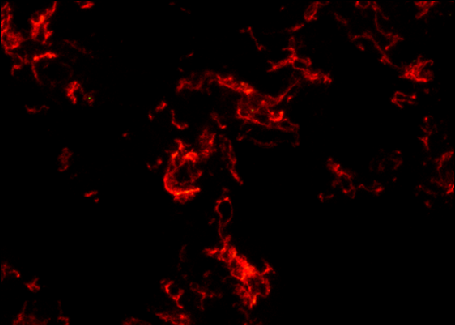

Supplement: Supplementary file 21 — Figure Source Data - EV and Appendix Figures [file 44319_2024_66_MOESM21_ESM.zip › Appendix_Source_Data/Appendix_Figure_S5/S5C_keratin19/S5C_KRT8KO_K19_High_Mag.tif]

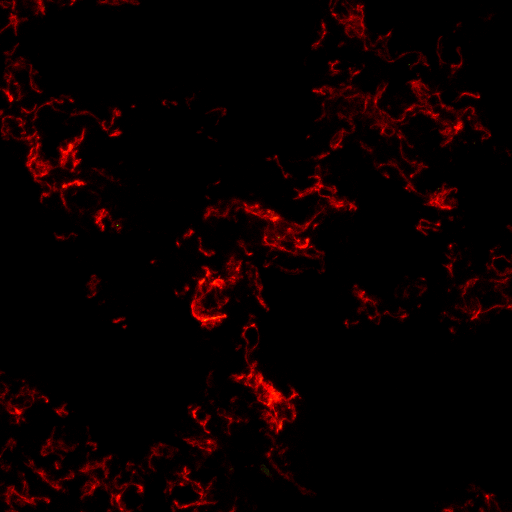

Supplement: Supplementary file 21 — Figure Source Data - EV and Appendix Figures [file 44319_2024_66_MOESM21_ESM.zip › Appendix_Source_Data/Appendix_Figure_S5/S5C_keratin19/S5C_KRT8KO_K19_High_Mag_Large.tif]

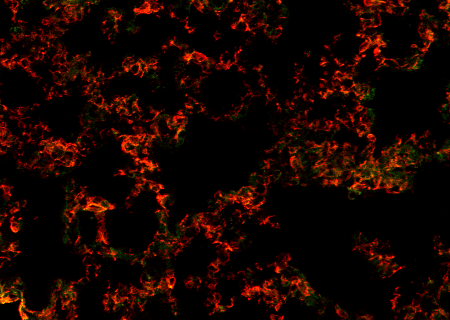

Supplement: Supplementary file 21 — Figure Source Data - EV and Appendix Figures [file 44319_2024_66_MOESM21_ESM.zip › Appendix_Source_Data/Appendix_Figure_S5/S5C_keratin19/S5C_KRT8KO_K19_Low_Mag.tif]

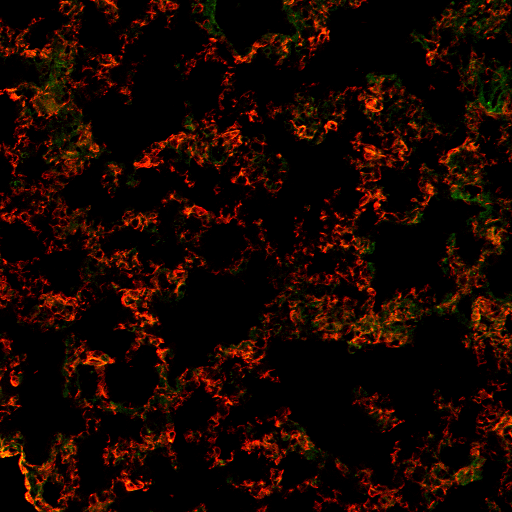

Supplement: Supplementary file 21 — Figure Source Data - EV and Appendix Figures [file 44319_2024_66_MOESM21_ESM.zip › Appendix_Source_Data/Appendix_Figure_S5/S5C_keratin19/S5C_KRT8KO_K19_Low_Mag_Large.tif]

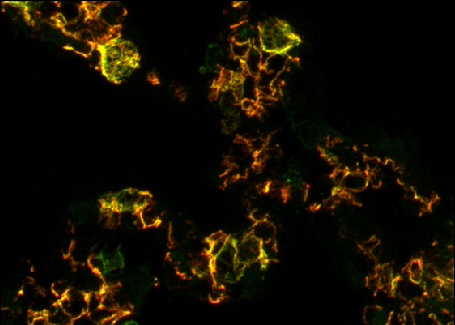

Supplement: Supplementary file 21 — Figure Source Data - EV and Appendix Figures [file 44319_2024_66_MOESM21_ESM.zip › Appendix_Source_Data/Appendix_Figure_S5/S5C_keratin19/S5C_WT_K19_High_Mag.tif]

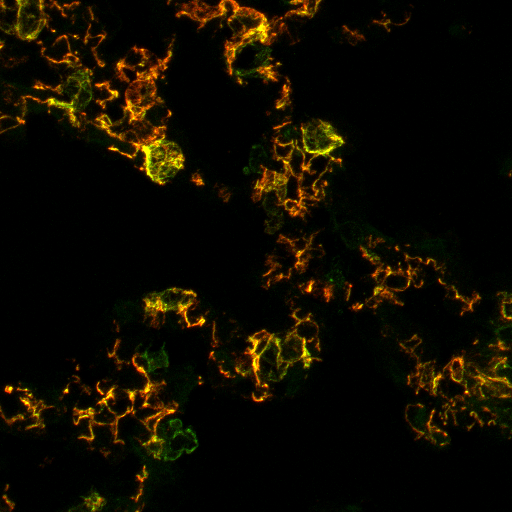

Supplement: Supplementary file 21 — Figure Source Data - EV and Appendix Figures [file 44319_2024_66_MOESM21_ESM.zip › Appendix_Source_Data/Appendix_Figure_S5/S5C_keratin19/S5C_WT_K19_High_Mag_Large.tif]

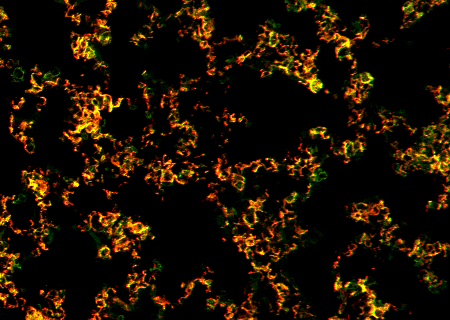

Supplement: Supplementary file 21 — Figure Source Data - EV and Appendix Figures [file 44319_2024_66_MOESM21_ESM.zip › Appendix_Source_Data/Appendix_Figure_S5/S5C_keratin19/S5C_WT_K19_Low_Mag.tif]

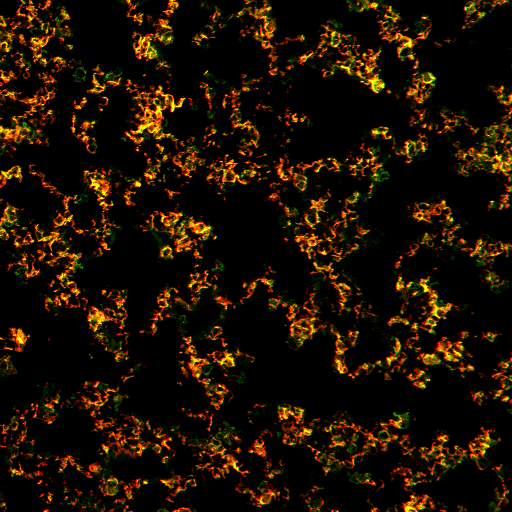

Supplement: Supplementary file 21 — Figure Source Data - EV and Appendix Figures [file 44319_2024_66_MOESM21_ESM.zip › Appendix_Source_Data/Appendix_Figure_S5/S5C_keratin19/S5C_WT_K19_Low_Mag_Large.tif]

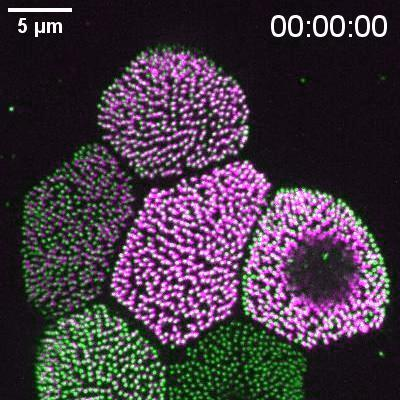

Supplement: Supplementary file 21 — Figure Source Data - EV and Appendix Figures [file 44319_2024_66_MOESM21_ESM.zip › Expanded_View_Source_Data/Figure_EV1/EV1A_MTEC_Live/EV1A_Movie_EV1(97_images).tif]

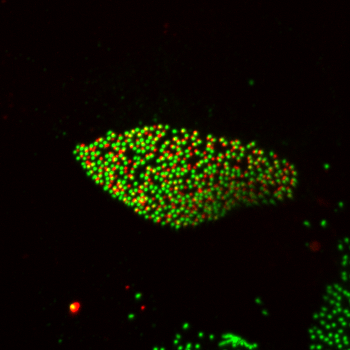

Supplement: Supplementary file 21 — Figure Source Data - EV and Appendix Figures [file 44319_2024_66_MOESM21_ESM.zip › Expanded_View_Source_Data/Figure_EV3/EV3A-D_Initial_TP_Analysis/Initial_TP_DMSO_Analysis(Cell_1-15).tif]

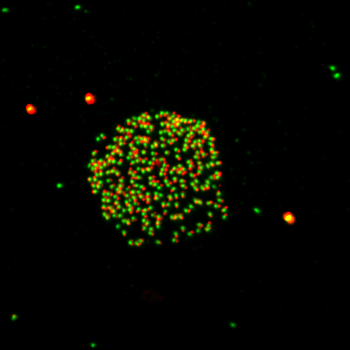

Supplement: Supplementary file 21 — Figure Source Data - EV and Appendix Figures [file 44319_2024_66_MOESM21_ESM.zip › Expanded_View_Source_Data/Figure_EV3/EV3A-D_Initial_TP_Analysis/Initial_TP_Noc_Analysis(Cell_1-18).tif]

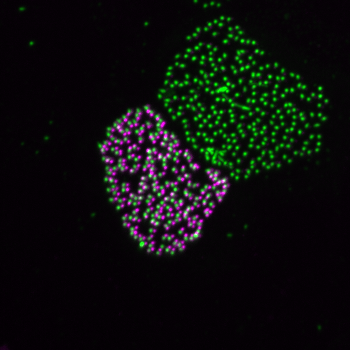

Supplement: Supplementary file 21 — Figure Source Data - EV and Appendix Figures [file 44319_2024_66_MOESM21_ESM.zip › Expanded_View_Source_Data/Figure_EV3/EV3A_MTEC_BBBF/EV3A_BBBF_Initial_TP_DMSO.tif]

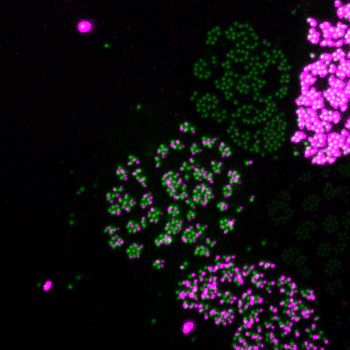

Supplement: Supplementary file 21 — Figure Source Data - EV and Appendix Figures [file 44319_2024_66_MOESM21_ESM.zip › Expanded_View_Source_Data/Figure_EV3/EV3A_MTEC_BBBF/EV3A_BBBF_Initial_TP_Noc.tif]

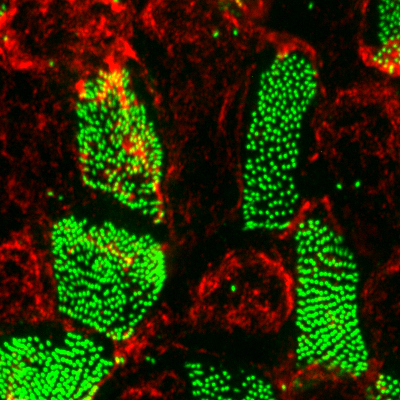

Supplement: Supplementary file 21 — Figure Source Data - EV and Appendix Figures [file 44319_2024_66_MOESM21_ESM.zip › Expanded_View_Source_Data/Figure_EV5/EV5A_trachea_keratin19/EV5A_KRT8-KO_trachea_DMSO_K19.tif]

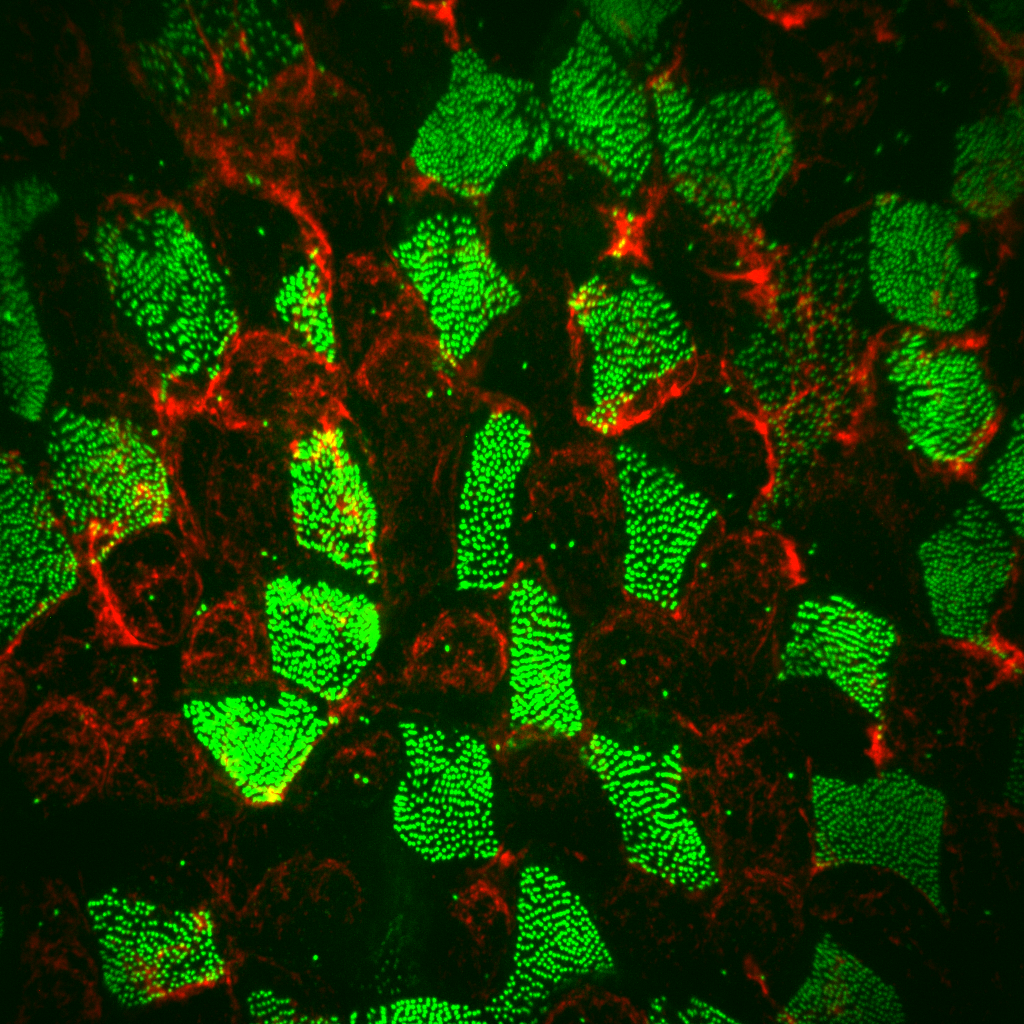

Supplement: Supplementary file 21 — Figure Source Data - EV and Appendix Figures [file 44319_2024_66_MOESM21_ESM.zip › Expanded_View_Source_Data/Figure_EV5/EV5A_trachea_keratin19/EV5A_KRT8-KO_trachea_DMSO_K19_Large.tif]

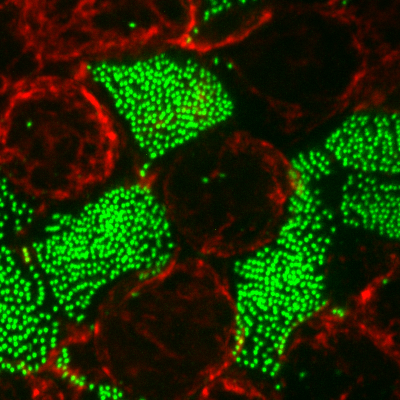

Supplement: Supplementary file 21 — Figure Source Data - EV and Appendix Figures [file 44319_2024_66_MOESM21_ESM.zip › Expanded_View_Source_Data/Figure_EV5/EV5A_trachea_keratin19/EV5A_KRT8-KO_trachea_Noc_K19.tif]

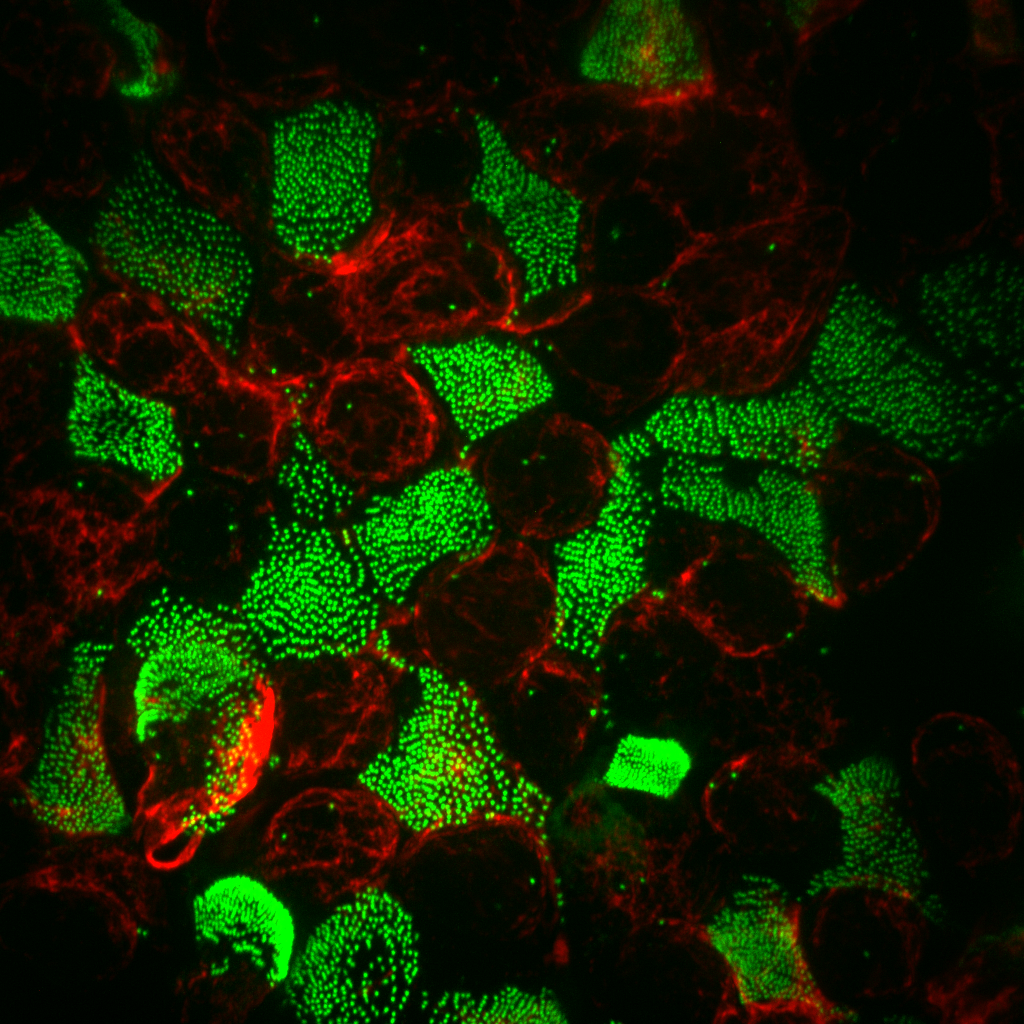

Supplement: Supplementary file 21 — Figure Source Data - EV and Appendix Figures [file 44319_2024_66_MOESM21_ESM.zip › Expanded_View_Source_Data/Figure_EV5/EV5A_trachea_keratin19/EV5A_KRT8-KO_trachea_Noc_K19_Large.tif]

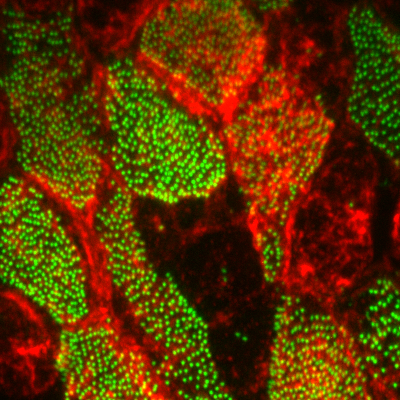

Supplement: Supplementary file 21 — Figure Source Data - EV and Appendix Figures [file 44319_2024_66_MOESM21_ESM.zip › Expanded_View_Source_Data/Figure_EV5/EV5A_trachea_keratin19/EV5A_WT_trachea_DMSO_K19.tif]

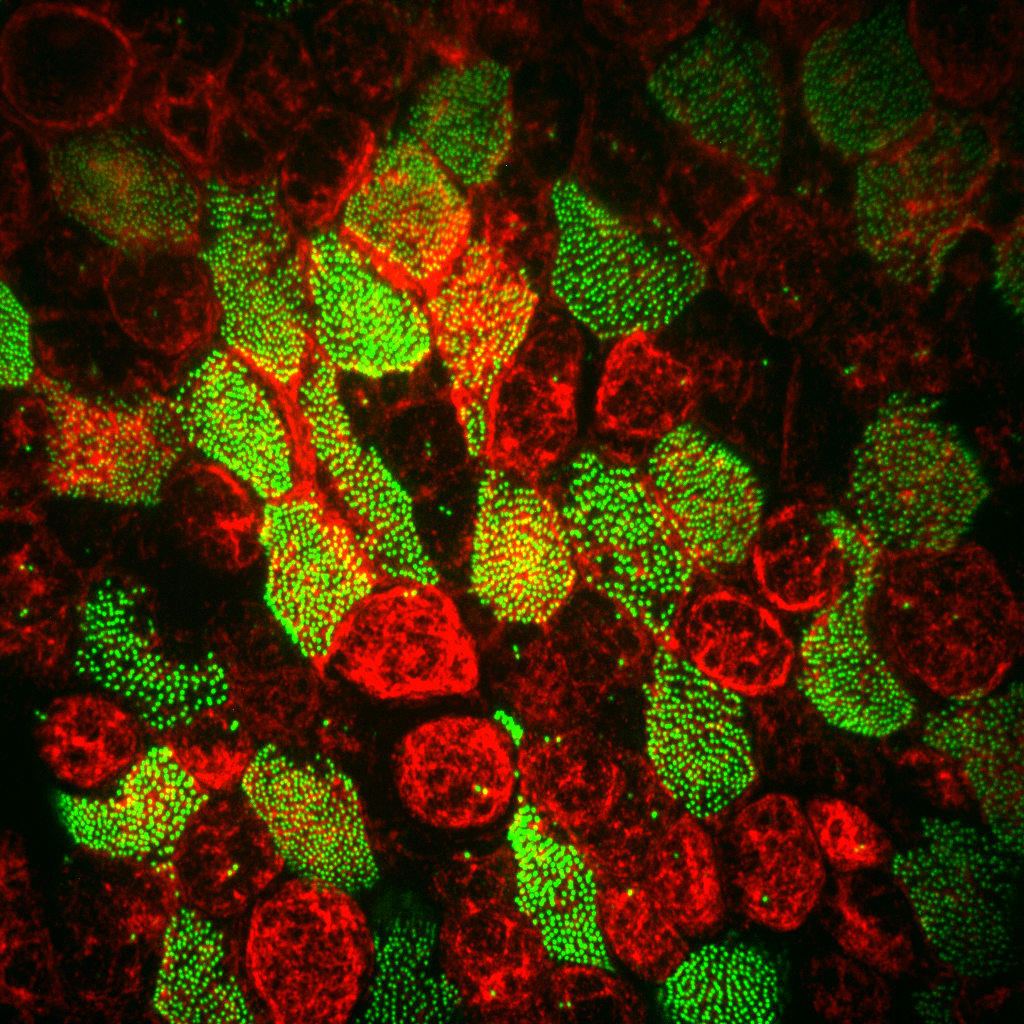

Supplement: Supplementary file 21 — Figure Source Data - EV and Appendix Figures [file 44319_2024_66_MOESM21_ESM.zip › Expanded_View_Source_Data/Figure_EV5/EV5A_trachea_keratin19/EV5A_WT_trachea_DMSO_K19_Large.tif]

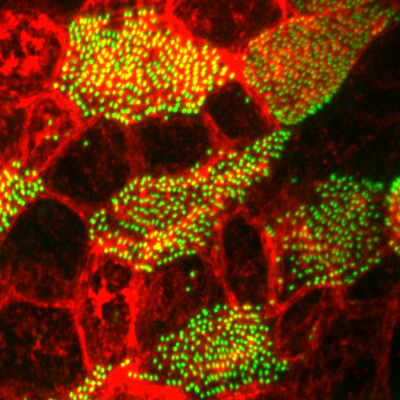

Supplement: Supplementary file 21 — Figure Source Data - EV and Appendix Figures [file 44319_2024_66_MOESM21_ESM.zip › Expanded_View_Source_Data/Figure_EV5/EV5A_trachea_keratin19/EV5A_WT_trachea_Noc_K19.tif]

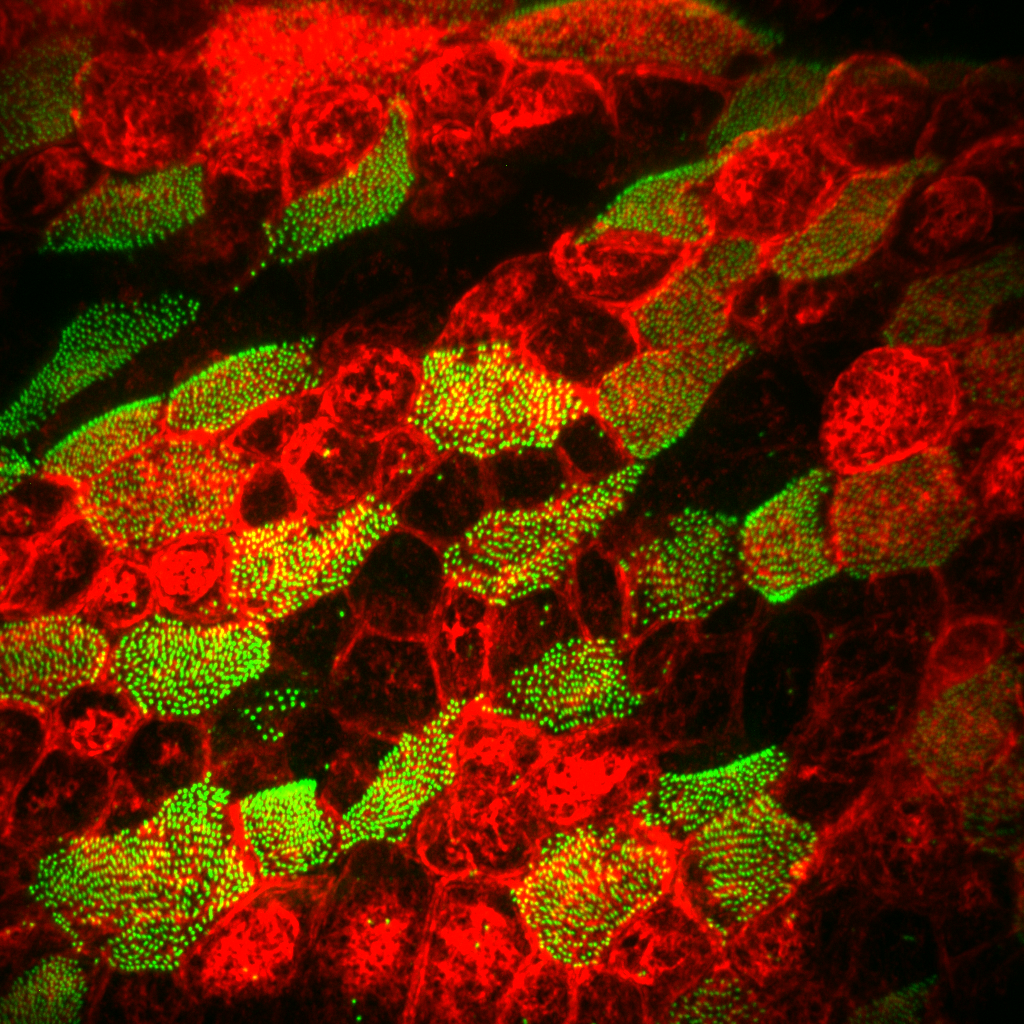

Supplement: Supplementary file 21 — Figure Source Data - EV and Appendix Figures [file 44319_2024_66_MOESM21_ESM.zip › Expanded_View_Source_Data/Figure_EV5/EV5A_trachea_keratin19/EV5A_WT_trachea_Noc_K19_Large.tif]
