# Supplementary material for: Dual-color live imaging unveils stepwise organization of multiple basal body arrays by cytoskeletons
Source: EMBO Rep. 2024 Feb 5;25(3):1176–207. doi: 10.1038/s44319-024-00066-0 (PMC10933483; doi:10.1038/s44319-024-00066-0)
Supplement: Supplementary file 23 — Expanded View Figures [file 44319_2024_66_MOESM23_ESM.pdf]

Expanded View Figures

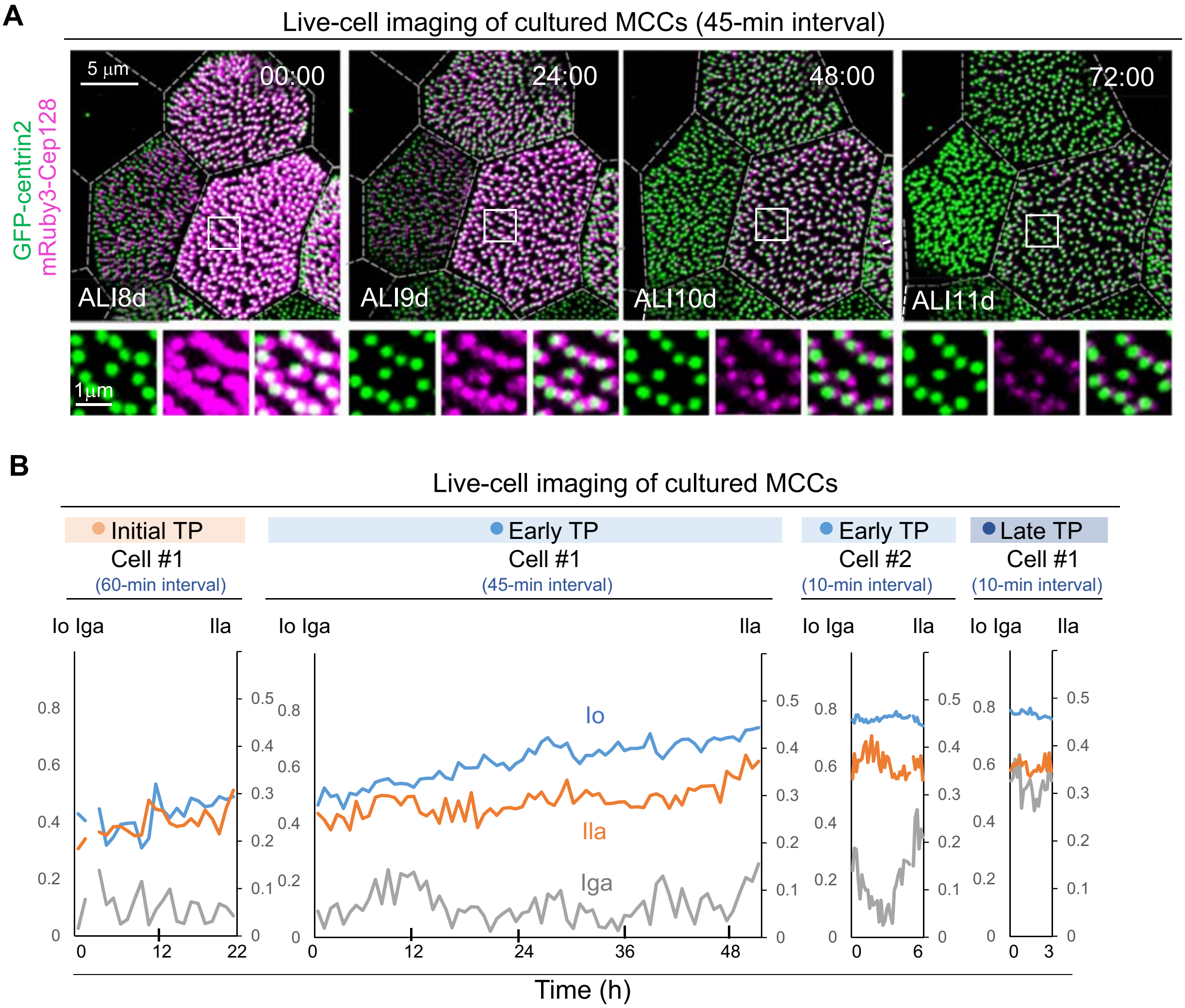

Figure EV1. Live-cell imaging of BB-orientation and BB-alignment in MCCs of MTECs.

(**A**) Long-term dual-color live-cell imaging of MTECs prepared from transgenic mice expressing GFP-centrin2 (green) and mRuby3-Cep128 (magenta). Images were captured every 45 min. We found that mRuby3-Cep128 signals were relatively unstable compared with GFP-centrin2 signals when using long-term live-cell imaging for 3 days. Insets show 3.2-fold magnified images of fluorescent foci. Gray dotted lines represent cell shapes estimated from the background signals. Time is denoted in hours: minutes. Bars, 5 μm. See also Movie EV1. (**B**) Line graphs of Io (blue)/IIa (orange)/Iga (gray) calculated from the MCCs shown in Fig. 2B–E. Specifically, Initial TP Cell #1 is based on data relevant to Fig. 2B; Early TP Cell #1 on data relevant to Fig. 2C; Early TP Cell#2 on data relevant to Fig. 2D; Late TP Cell #1 on data relevant to Fig. 2E. Note that increases in Io and IIa values occurred before the increase in the Iga value. Values were plotted in the scatter plot graphs shown in Fig. 2F, G. See also Movies EV2–5. Source data are available online for this figure.

© The Author(s)

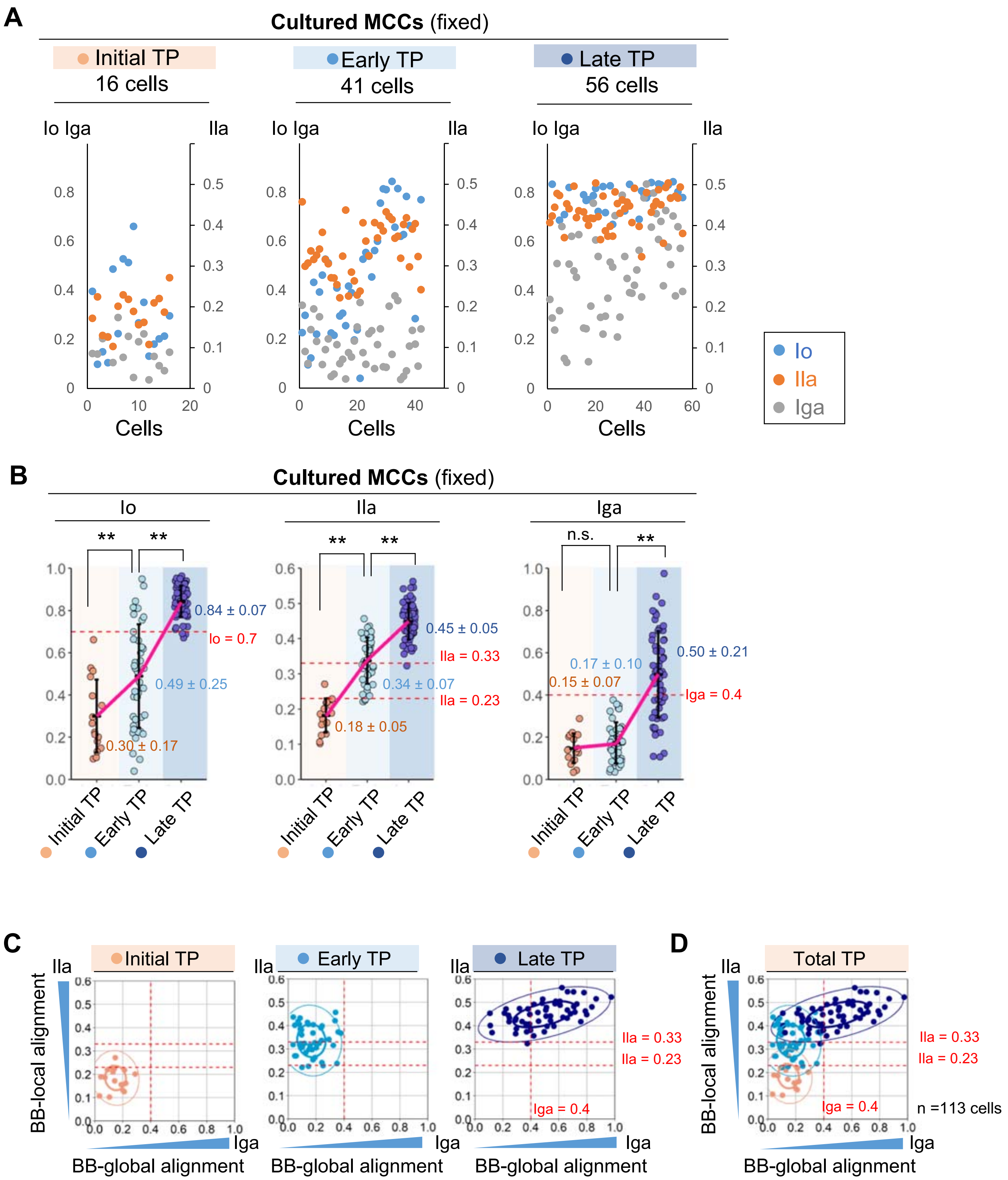
A
Cultured MCCs (fixed)
Initial TP
16 cells
Early TP
41 cells
Late TP
56 cells
Io Iga
IIa
Cells
Io
IIa
Iga
B
Cultured MCCs (fixed)
Io
IIa
Iga
**
n.s.
0.84 ± 0.07
Io = 0.7
0.49 ± 0.25
0.30 ± 0.17
0.45 ± 0.05
IIa = 0.33
0.34 ± 0.07
IIa = 0.23
0.18 ± 0.05
0.17 ± 0.10
0.15 ± 0.07
0.50 ± 0.21
Iga = 0.4
Initial TP
Early TP
Late TP
C
D
Total TP
BB-local alignment
BB-global alignment
n =113 cells

Figure EV2. Quantification of BB-orientation and BB-alignment in MCCs of fixed MTECs at different TPs.

(**A**) Dot plots of Io (blue)/IIa (orange)/Iga (gray) in fixed MCCs of MTECs prepared from transgenic mice expressing GFP-centrin2 and mRuby3-Cep128 at initial ($n = 16$ cells), early ($n = 41$ cells), and late ($n = 56$ cells) timepoints (TPs). (**B**) Dot plots of the Io/IIa/Iga in fixed MCCs of MTECs prepared from transgenic mice expressing GFP-centrin2 and mRuby3-Cep128 at the initial (pink, $n = 16$ cells), early (sky blue, $n = 41$ cells), and late (navy blue, $n = 56$ cells) TPs. Red dotted lines represent Io = 0.7, IIa = 0.23/0.33, and Iga = 0.4. Values are means ± standard deviations of each index. **$P < 0.01$, n.s., not significant (Kruskal–Wallis test with pairwise-comparison test). (**C**,**D**) Scatter plots of IIa/Iga in MCCs of MTECs prepared from transgenic mice expressing GFP-centrin2 and mRuby3-Cep128 at initial (pink, $n = 16$ cells), early (blue, $n = 41$ cells), and late (navy blue, $n = 56$ cells) TPs (**C**). Scatter plots from total TPs are also shown in (**D**). Red dotted lines represent IIa = 0.23/0.33, and Iga = 0.4. In addition, 50% (thick line) and 95% (thin line) probability ellipses are shown. Source data are available online for this figure.

 © The Author(s)

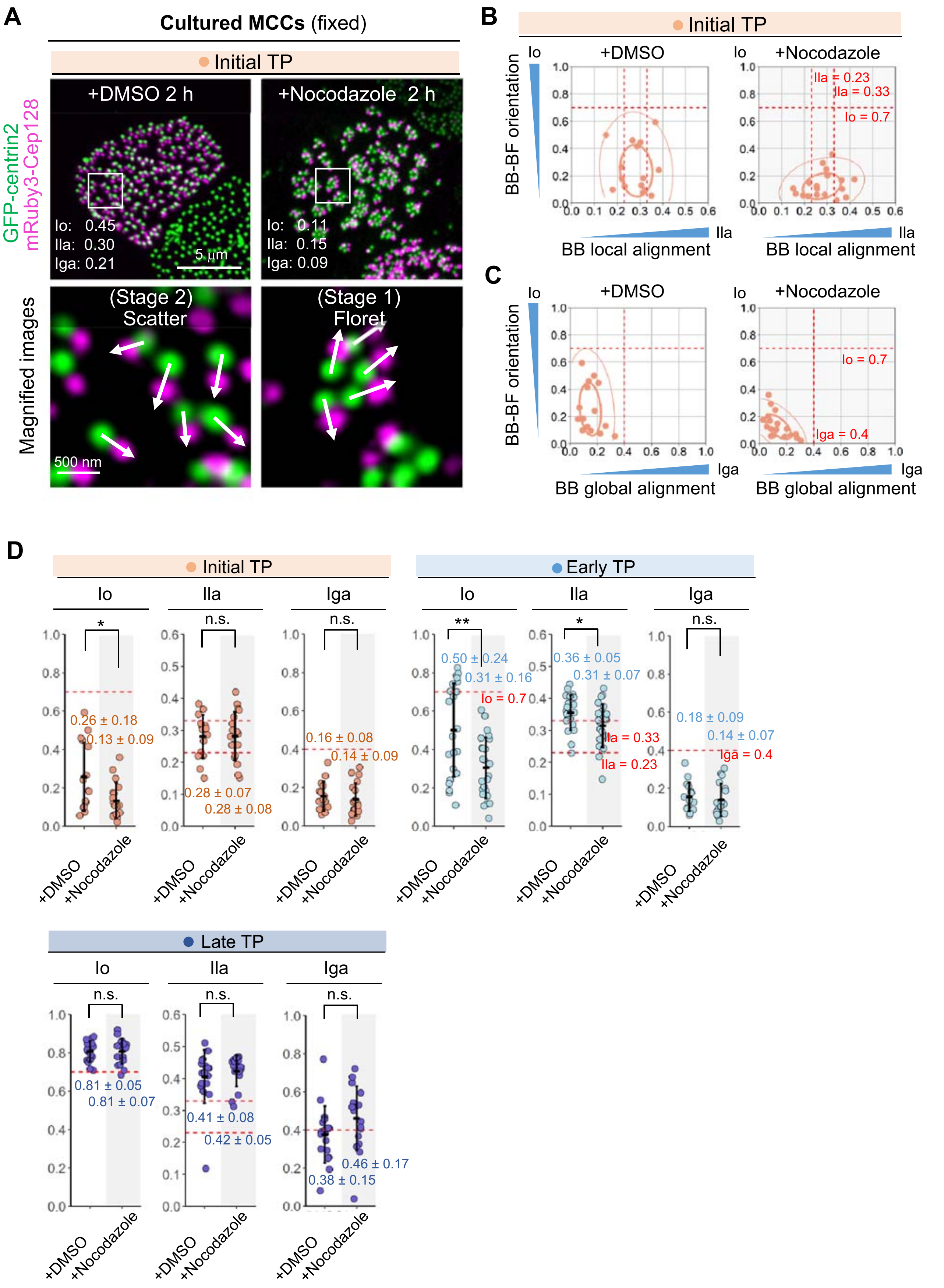
A
Cultured MCCs (fixed)
Initial TP
+DMSO 2 h
+Nocodazole 2 h
GFP-centrin2
mRuby3-Cep128
Io: 0.45
IIa: 0.30
Iga: 0.21
5 μm
Io: 0.11
IIa: 0.15
Iga: 0.09
Magnified images
(Stage 2) Scatter
(Stage 1) Floret
500 nm
B
Initial TP
+DMSO
+Nocodazole
BB-BF orientation
BB local alignment
IIa = 0.23
IIa = 0.33
Io = 0.7
C
BB global alignment
Iga = 0.4
D
Initial TP
Early TP
Late TP
Io
IIa
Iga
n.s.
0.26 ± 0.18
0.13 ± 0.09
0.28 ± 0.07
0.28 ± 0.08
0.16 ± 0.08
0.14 ± 0.09
0.50 ± 0.24
0.31 ± 0.16
0.36 ± 0.05
0.31 ± 0.07
0.18 ± 0.09
0.14 ± 0.07
0.81 ± 0.05
0.81 ± 0.07
0.41 ± 0.08
0.42 ± 0.05
0.38 ± 0.15
0.46 ± 0.17
+DMSO
+Nocodazole

Figure EV3. Quantitative analysis of the effects of nocodazole treatment on MCCs of MTECs at different TPs.

(**A**) Spinning disk confocal microscopy the BB-array in MCCs of fixed MTECs prepared from transgenic mice expressing GFP-centrin2 (green) and mRuby3-Cep128 (magenta) at the initial TP after treatment with DMSO or 6.6 μM nocodazole for 2 h. Insets show 6.7-fold magnified images of fluorescent foci. Bars, 5 μm and 500 nm. (**B**,**C**) Scatter plots of Io/Ila (**B**) and Io/Iga (**C**) in fixed MTECs prepared from transgenic mice expressing GFP-centrin2 and mRuby3-Cep128 at the initial TP after treatment with DMSO or 6.6 μM nocodazole for 2 h (DMSO, $n = 15$ cells; nocodazole, $n = 18$ cells). Red dotted lines represent Io =0.7, Ila = 0.23/0.33, and Iga = 0.4. In addition, 50% (thick line) and 95% (thin line) probability ellipses are shown. (**D**) Dot plots of Io/Ila/Iga in MCCs of fixed MTECs prepared from transgenic mice expressing GFP-centrin2 and mRuby3-Cep128 at the at initial (pink, $n = 15$ cells and $n = 18$ cells), early (sky blue, $n = 22$ cells and 24 cells), and late (navy blue, $n = 18$ cells and $n = 17$ cells) TPs after treatment with DMSO or 6.6 μM nocodazole for 2 h. Red dotted lines represent Io = 0.7, Ila = 0.23/0.33, and Iga = 0.4. Values are means ± standard deviations of each index. *$P < 0.05$, **$P < 0.01$, n.s., not significant (Brunner-Munzel test). Source data are available online for this figure.

© The Author(s)

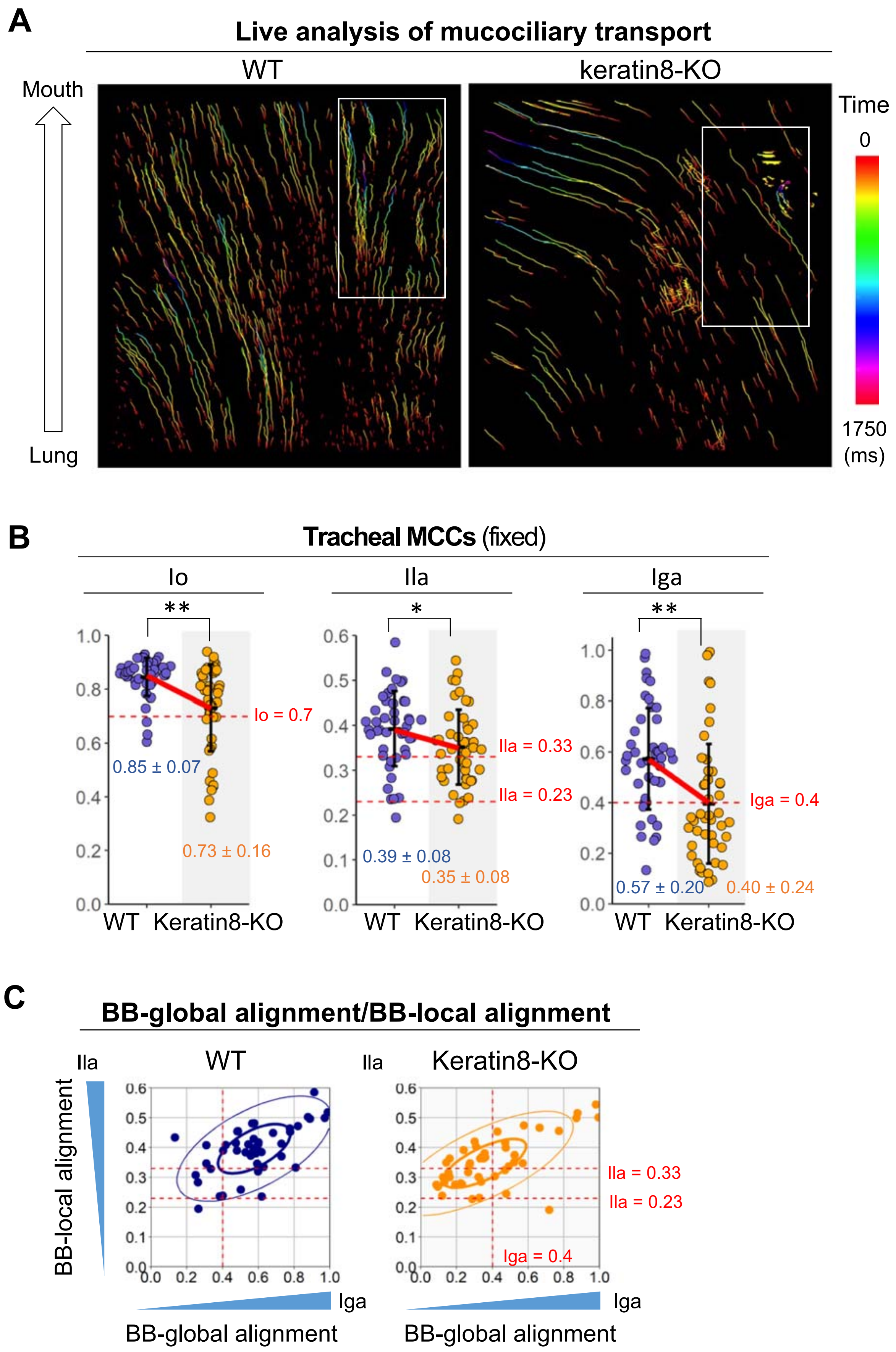
A
Live analysis of mucociliary transport
WT
keratin8-KO
Mouth
Lung
Time
0
1750
(ms)
B
Tracheal MCCs (fixed)
Io
IIa
Iga
**
*
**
Io = 0.7
0.85 ± 0.07
0.73 ± 0.16
IIa = 0.33
IIa = 0.23
0.39 ± 0.08
0.35 ± 0.08
Iga = 0.4
0.57 ± 0.20
0.40 ± 0.24
WT Keratin8-KO
C
BB-global alignment/BB-local alignment
WT
Keratin8-KO
IIa
BB-local alignment
Iga
BB-global alignment
IIa = 0.33
IIa = 0.23
Iga = 0.4

Figure EV4. Analysis of mucociliary clearance and the BB-array using keratin8-KO mice.

(**A**) Analysis of mucociliary clearance in the tracheas of adult wild-type and keratin8-KO mice using live imaging of fluorescent beads. High-magnification images of the boxed regions are shown in Fig. 7C. (**B**) Dot plots of the Io/IIa/Iga in fixed MCCs of tracheal cells prepared from wild-type or keratin8-KO mice shown in Fig. 7 (wild-type, navy blue, $n = 45$ cells; keratin8-KO, orange, $n = 45$ cells). Values are means ± standard deviations of each index. *$P < 0.05$, **$P < 0.01$ (Brunner-Munzel test). Red dotted lines represent Io = 0.7, IIa = 0.23/0.33, and Iga = 0.4. (**C**) Scatter plots of IIa/Iga in MCCs of tracheal cells prepared from wild-type or keratin8-KO mice shown in Fig. 7 (wild-type, navy blue, $n = 45$ cells; keratin8-KO, orange, $n = 45$ cells). Red dotted lines represent IIa = 0.23/0.33, and Iga = 0.4. In addition, 50% (thick line) and 95% (thin line) probability ellipses are shown. SSource data are available online for this figure.

© The Author(s)

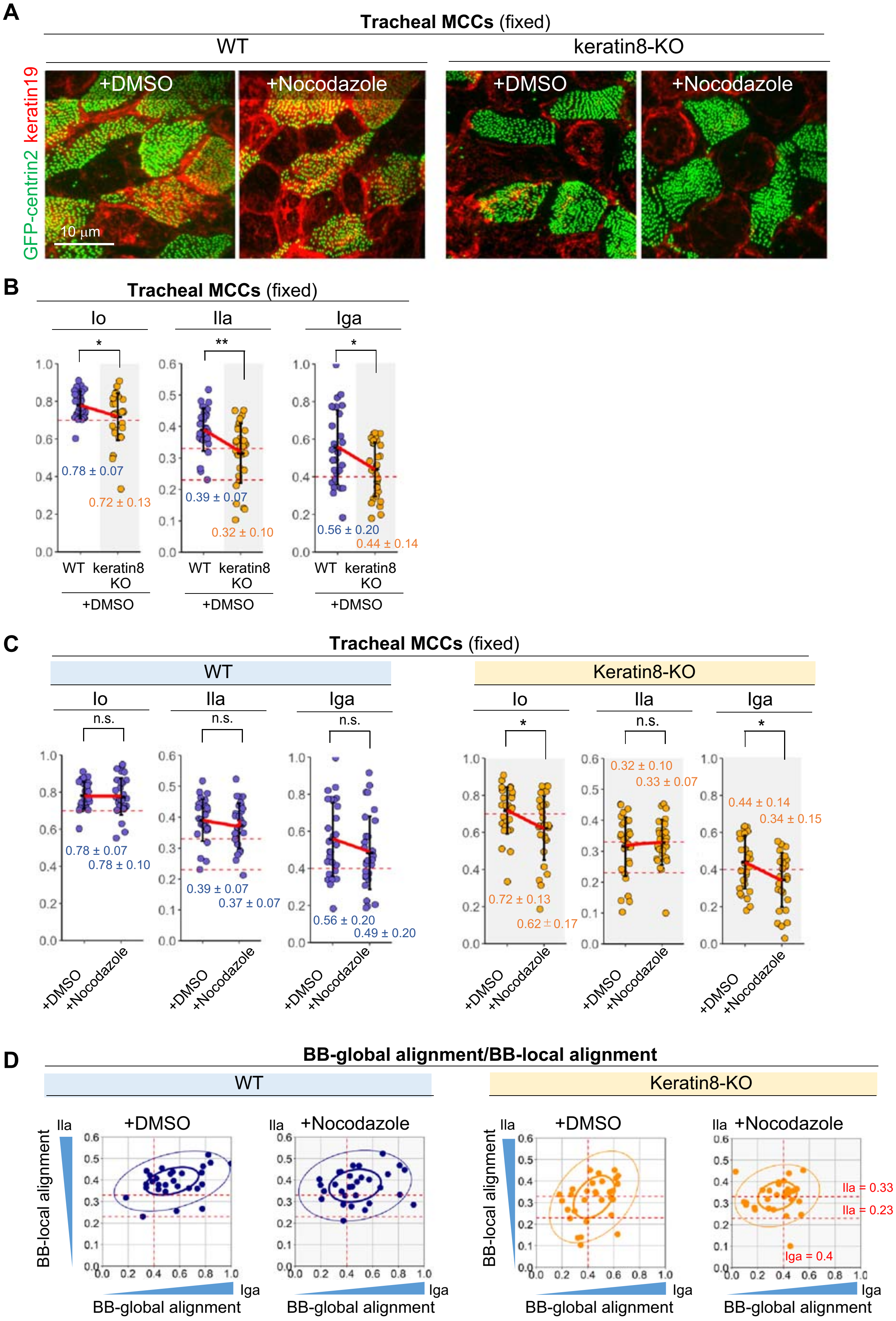
A
Tracheal MCCs (fixed)
WT
keratin8-KO
+DMSO
+Nocodazole
GFP-centrin2 keratin19
10 µm
B
Tracheal MCCs (fixed)
Io
IIa
Iga
0.78 ± 0.07
0.72 ± 0.13
0.39 ± 0.07
0.32 ± 0.10
0.56 ± 0.20
0.44 ± 0.14
WT keratin8 KO
+DMSO
C
Tracheal MCCs (fixed)
WT
Keratin8-KO
n.s.
0.78 ± 0.07
0.78 ± 0.10
0.39 ± 0.07
0.37 ± 0.07
0.56 ± 0.20
0.49 ± 0.20
0.72 ± 0.13
0.62 ± 0.17
0.32 ± 0.10
0.33 ± 0.07
0.44 ± 0.14
0.34 ± 0.15
+DMSO
+Nocodazole
D
BB-global alignment/BB-local alignment
BB-local alignment
BB-global alignment
IIa = 0.33
IIa = 0.23
Iga = 0.4

Figure EV5. Quantitative analysis of the effects of nocodazole treatment on MCCs of tracheal MTECs.

(**A**) Spinning disk confocal microscopy of MCCs of fixed tracheal cells prepared from wild-type and keratin8-KO mice expressing GFP-centrin2 (green) and mRuby3-Cep128. MCCs of tracheal cells were fixed and stained using anti-α-keratin19 antibody (red) after treatment with DMSO or 9.9 μM nocodazole for 2 h. Bar, 10 μm. (**B**) Dot plots of the Io/IIa/Iga in fixed MCCs of tracheal cells prepared from wild-type or keratin8-KO mice after treatment with DMSO for 2 h as shown in Fig. 8 (wild-type, navy blue, $n = 28$ cells; keratin8-KO, orange, $n = 30$ cells). Values are means ± standard deviations of each index. *$P < 0.05$, **$P < 0.01$ (Brunner-Munzel test). Red dotted lines represent Io = 0.7, IIa = 0.23/0.33, and Iga = 0.4. (**C**) Dot plots of the Io/IIa/Iga in fixed MCCs of tracheal cells prepared from wild-type or keratin8-KO mice after treatment with DMSO or 9.9 μM nocodazole for 2 h as shown in Fig. 8 (wild-type, navy blue, $n = 28$ cells and $n = 30$ cells; keratin8-KO, orange, $n = 30$ cells and $n = 28$ cells). Values are means ± standard deviations of each index. *$P < 0.05$, **$P < 0.01$, n.s., not significant (Brunner-Munzel test). Red dotted lines represent Io = 0.7, IIa = 0.23/0.33, and Iga = 0.4. (**D**) Scatter plots of IIa/Iga in MCCs of tracheal cells prepared from wild-type (navy blue, $n = 28$ cells and $n = 30$ cells) or keratin8-KO (orange, $n = 30$ cells and $n = 28$ cells) mice after treatment with DMSO or 9.9 μM nocodazole for 2 h. Red dotted lines represent IIa = 0.23/0.33 and Iga = 0.4. In addition, 50% (thick line) and 95% (thin line) probability ellipses are shown. Source data are available online for this figure.

 © The Author(s)
